# Randomized phase I trial HIV-CORE 003: Depletion of serum amyloid P component and immunogenicity of DNA vaccination against HIV-1

**DOI:** 10.1371/journal.pone.0197299

**Published:** 2018-05-17

**Authors:** Nicola J. Borthwick, Thirusha Lane, Nathifa Moyo, Alison Crook, Jung Min Shim, Ian Baines, Edmund G. Wee, Philip N. Hawkins, Julian D. Gillmore, Tomáš Hanke, Mark B. Pepys

**Affiliations:** 1 The Jenner Institute, Nuffield Department of Medicine, University of Oxford, Oxford, United Kingdom; 2 Centre for Amyloidosis and Acute Phase Proteins, University College London, London, United Kingdom; 3 International Research Center for Medical Sciences, Kumamoto University, Kumamoto, Japan; 4 Wolfson Drug Discovery Unit, Centre for Amyloidosis and Acute Phase Proteins, UCL, London, United Kingdom; Public Health England, UNITED KINGDOM

## Abstract

**Background:**

The failure of DNA vaccination in humans, in contrast to its efficacy in some species, is unexplained. Observational and interventional experimental evidence suggests that DNA immunogenicity may be prevented by binding of human serum amyloid P component (SAP). SAP is the single normal DNA binding protein in human plasma. The drug (R)-1-[6-[(R)-2-carboxypyrrolidin-1-yl]-6-oxo-hexanoyl]pyrrolidine-2-carboxylic acid (CPHPC, miridesap), developed for treatment of systemic amyloidosis and Alzheimer’s disease, depletes circulating SAP by 95–99%. The proof-of-concept HIV-CORE 003 clinical trial tested whether SAP depletion by CPHPC would enhance the immune response in human volunteers to DNA vaccination delivering the HIVconsv immunogen derived from conserved sub-protein regions of HIV-1.

**Methods:**

Human volunteers received 3 intramuscular immunizations with an experimental DNA vaccine (DDD) expressing HIV-1-derived immunogen HIVconsv, with or without prior depletion of SAP by CPHPC. All subjects were subsequently boosted by simian (chimpanzee) adenovirus (C)- and poxvirus MVA (M)-vectored vaccines delivering the same immunogen. After administration of each vaccine modality, the peak total magnitudes, kinetics, functionality and memory subsets of the T-cell responses to HIVconsv were thoroughly characterized.

**Results:**

No differences were observed between the CPHPC treated and control groups in any of the multiple quantitative and qualitative parameters of the T-cell responses to HIVconsv, except that after SAP depletion, there was a statistically significantly greater breadth of T-cell specificities, that is the number of recognized epitopes, following the DDDC vaccination.

**Conclusions:**

The protocol used here for SAP depletion by CPHPC prior to DNA vaccination produced only a very modest suggestion of enhanced immunogenicity. Further studies will be required to determine whether SAP depletion might have a practical value in DNA vaccination for other plasmid backbones and/or immunogens.

**Trial registration:**

Clinicaltrials.gov NCT02425241

## Introduction

Parenteral injection of naked DNA encoding pathogen or cancer-derived immunogens is a highly desirable approach to vaccination but, for unknown reasons, it is not efficacious in some species including humans. Serum amyloid P component (SAP) is the single normal human plasma protein that binds avidly to DNA under physiological conditions [1]. Studying different animal species, we found a complete concordance between the efficacy of DNA vaccination and either the complete absence of SAP or the presence of SAP that does not bind strongly to DNA. For example, mice respond well to DNA vaccination and have SAP that binds DNA very weakly [2]. However, transgenic mice expressing human SAP lost potent murine immune responses to DNA vaccination and this inhibition was completely abrogated when the transgenic mice were treated with (R)-1-[6-[(R)-2-carboxy-pyrrolidin-1-yl]-6-oxo-hexanoyl]pyrrolidine-2-carboxylic acid (CPHPC, miridesap) [3, 4], the SAP-depleting drug [5]. In order to investigate whether human SAP might interfere with the immunogenicity of DNA vaccination in humans, we conducted a phase I/IIa clinical trial of DNA vaccination in subjects receiving infusion of CPHPC to deplete their circulating SAP at the time of each injection of DNA.

To tackle the enormous genetic plasticity of the human immunodeficiency virus type 1 (HIV-1), we pioneered a strategy, which focuses vaccine-elicited responses on the most conserved regions of the HIV-1 proteome [6]. The first generation HIV-1 conserved immunogen, HIVconsv [7], was presented to the immune system in the form of genetic vaccines using plasmid DNA, engineered non-replicating simian adenovirus ChAdV-63 and non-replicating poxvirus modified vaccinia virus Ankara (MVA) [8]. The pSG2.HIVconsv DNA, ChAdV63.HIVconsv and MVA.HIVconsv vaccines have been tested in a series of clinical trials in human volunteers and shown to be safe and the last two highly immunogenic [8–12]. Clinical lots of these vaccines were readily available for administration to human volunteers in the present study, which aimed to improve the immunogenicity of the plasmid DNA vaccine component.

## Materials and methods

### The HIV-CORE 003 trial

The phase I/IIa HIV-CORE 003 trial was conducted in London, UK between October 2013 and February 2016. The trial was approved by the West London National Research Ethics Service (NRES) Committee (Ref: 13/LO/0090) and by the UK Medicines and Healthcare products Regulatory Agency (Ref: 2012-004052-11). The study was conducted according to the principles of the Declaration of Helsinki (2008) and complied with the International Conference on Harmonization Good Clinical Practice guidelines. All volunteers gave written informed consent before participation. Tissue samples were stored and used in compliance with the UK Human Tissue Act 2004 and with approval from local NRES.

### Study population, vaccines, regimens and randomization

**Fig 1:**
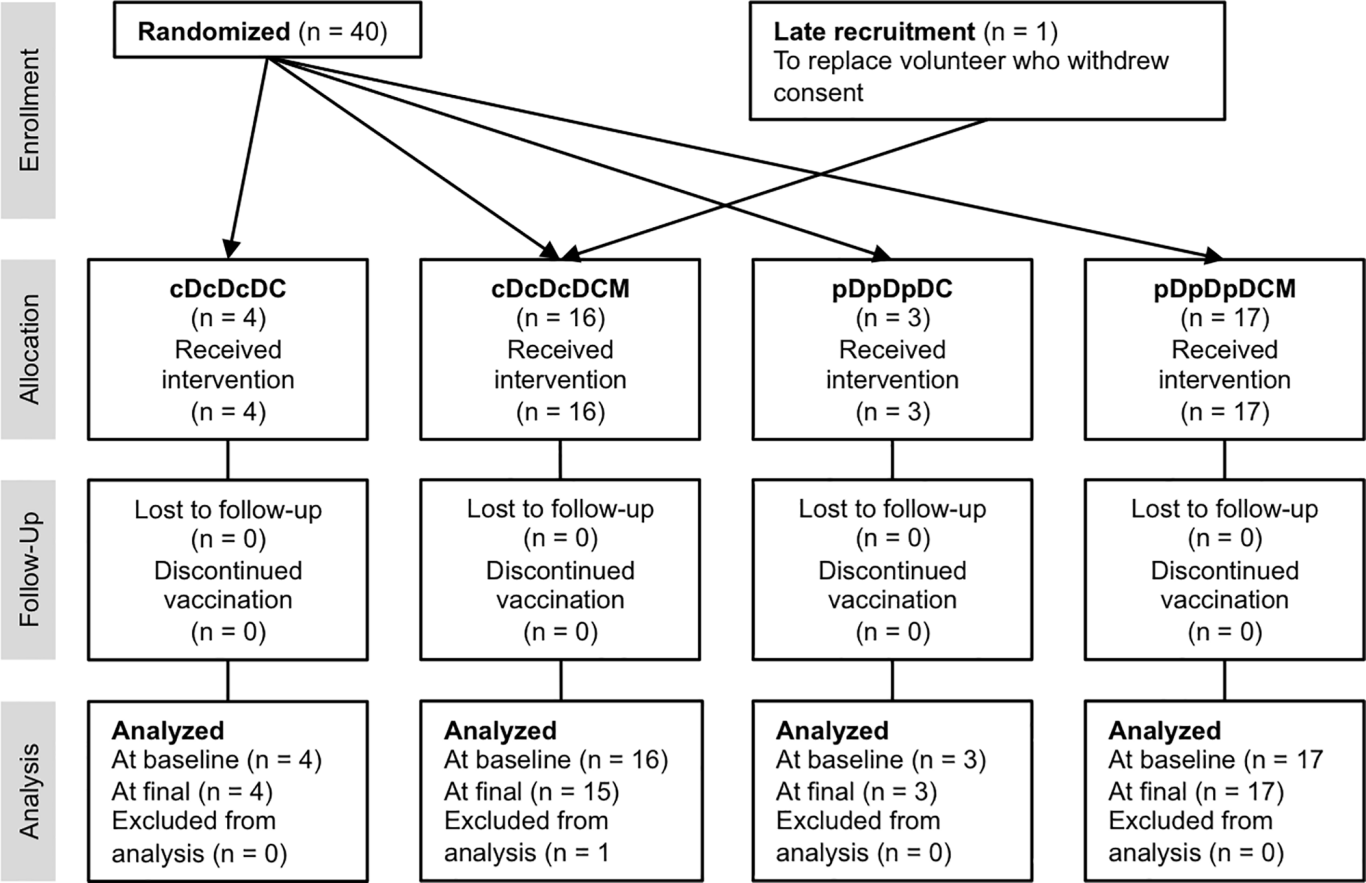
CONSORT flow diagram of the HIV-CORE 003 trial. All CPHPC-treated volunteers completed the study protocol. Among the 20 volunteers allocated to the placebo Group, 2 volunteers were lost to follow-up of whom 1 discontinued vaccinations.

**Fig 2:**
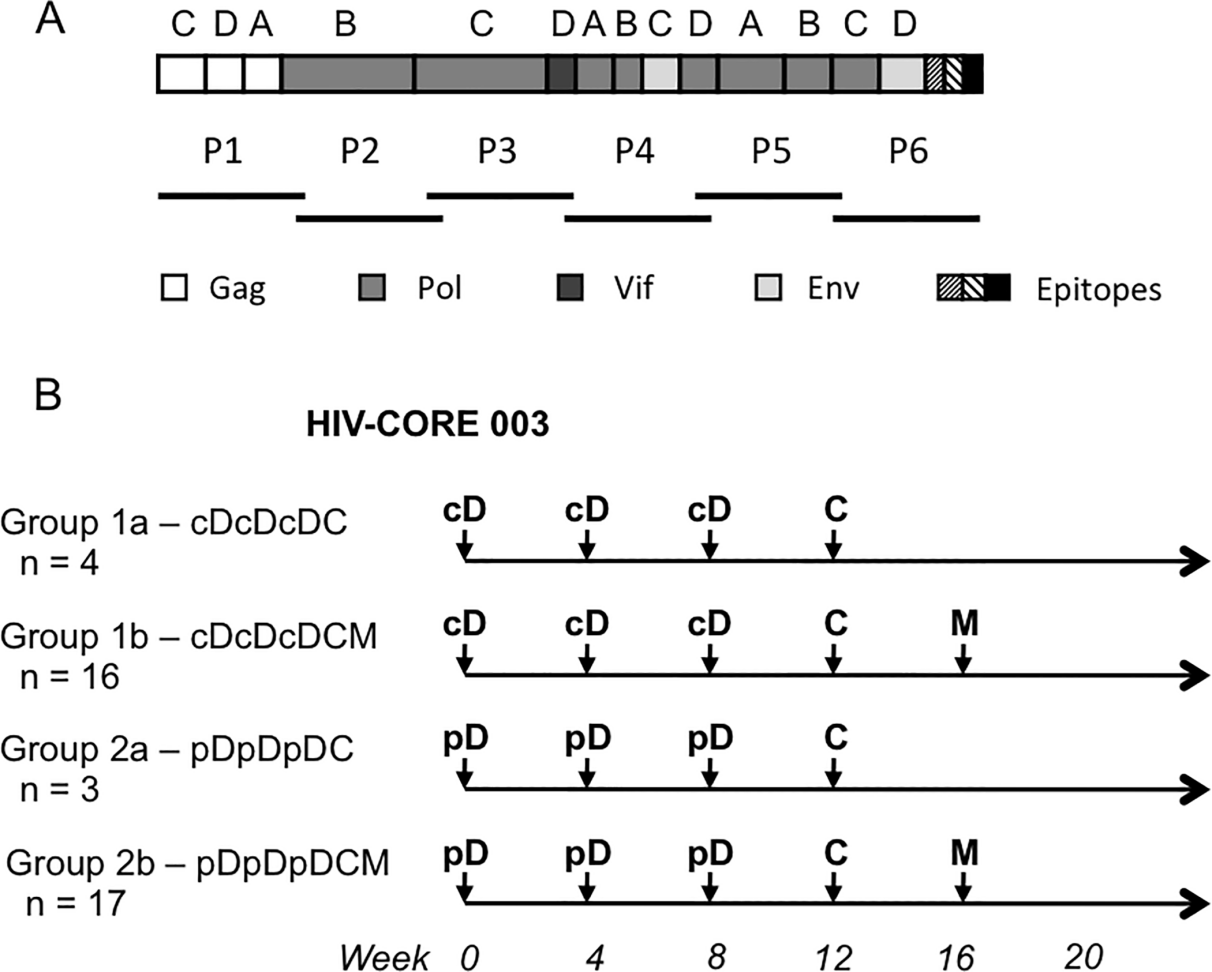
Vaccine immunogen HIVconsv and the design of the HIV-CORE 003 trial. (A) Schematic representations of the HIVconsv immunogen and six pools P1-P6 of a total of 199 overlapping peptides used for the detection of the human vaccine-elicited T-cell responses. HIVconsv is a chimaeric protein assembled from 14 highly conserved regions of the HIV-1 proteome, the HIV-1 protein origins of which are colour-coded below. (B) Volunteers in the phase I/IIa HIV-CORE 003 trial were randomized into Group 1 for depletion of serum amyloid P component (SAP) by a 26-hour infusion of drug CPHPC, or Group 2 receiving saline infusion alone as placebo, whereby the DNA was injected after 24 hours of infusion. All volunteers were boosted with non-replicating recombinant virus vaccines expressing the same HIVconsv immunogen as indicated. cD–pSG2.HIVconsv DNA with CPHPC infusion prior to DNA administration; pD–pSG2.HIVconsv with placebo infusion prior to DNA; C–non-replicating simian (chimpanzee) adenovirus-vectored vaccine ChAdV63.HIVconsv; and M–non-replicating poxvirus-vectored vaccine MVA.HIVconsv. The number of volunteers recruited into each study group is indicated.

Forty healthy, HIV-1-negative adult male volunteers at low risk of HIV-1 infection were enrolled (Fig 1) with mean (SD, range) age in years of 26 (6, 19–44) and including the following ethnicities: 24 white British, 6 white other, 3 white Irish, 2 Indian, 1 Pakistani, 1 other Asian, 1 mixed background. They each received experimental vaccines expressing the first-generation HIV-1 conserved region-derived immunogen, HIVconsv (Fig 2A) [7], which was delivered using plasmid DNA as pSG2.HIVconsv (D), engineered non-replicating simian adenovirus as ChAdV63.HIVconsv (C) and non-replicating poxvirus modified vaccinia virus Ankara as MVA.HIVconsv (M) combined into heterologous regimens (Fig 2B). Construction and manufacture of the vaccines were previously described [8, 10, 13]. We confirmed that human SAP binds avidly to the vaccine plasmid DNA with K_d_ estimated from the binding isotherm as ~150 nM. All vaccine doses were administered by intramuscular needle injection. At the outset, subjects were randomized to one of two groups cD or pD to receive an intravenous infusion of CPHPC at 40 mg/hour in the experimental group or saline in the placebo control group, respectively, before each dose of 4 mg of vaccine DNA. The DNA was injected 24 hours after the infusion started and the infusion was terminated 2 hours later. The trial was initially designed to compare the cDcDcDC (Groups 1a; n = 4) and pDpDpDC (Group 2a; n = 3) regimens, however, following recruitment of the first 7 volunteers, it was modified to the cDcDcDCM (Group 1b; n = 16) and pDpDpDCM (Group 2b; n = 17) regimens seeking to further amplify, with the additional MVA.HIVconsv boost, any differences in immunogenicity between the two groups. This Protocol modification was approved by the ethics committee,

### Power calculations for the group size

Power calculations were based on immunogenicity data obtained in trial HIV-CORE 002, group DDDCM [8], and calculated for 20 participants in each group in trial HIV-CORE 003. Thus, at 0–12 weeks (after DDD), whereby the mean ± SD peak response in HIV-CORE 002 was 73 ± 67 spot-forming units (SFU)/10^6^ PBMC, we had 96% power to detect a 10-fold increase. At 12–20 weeks (after DDDC), whereby the mean ± SD peak response was 1644 ± 1287 SFU/10^6^ PBMC, we had 86% power to detect a 2.3-fold increase. Finally, at 21–22 weeks (after DDDCM), whereby the mean ± SD peak response was 3285 ± 2091 SFU/10^6^ PBMC, we had 80% power to detect a 1.58-fold increase. Note, that the induced frequencies of specific T cells in HIV-CORE 002 were the highest published frequencies of vaccine-elicited T cells in the field of HIV-1 vaccines using subunit genetic vaccines and so it was not reasonable to expect huge increases from the DDDC and DDDCM peak responses. For comparison, the STEP study using three administrations a HAdV-5-vectored vaccine induced between 136–686 SFU/10^6^ PBMC [14].

### Isolation and cryopreservation of PBMC

Phlebotomy was performed as described before [15]. Blood was collected into heparinized vacutainers (Becton Dickinson) and processed within 6 hours. Isolated PBMC were cryopreserved using standard procedures [16].

### Peptides and antigens

HIVconsv-derived 15-mer peptides overlapping by 11 amino acids and spanning the entire HIVconsv protein (Ana-Spec, San Jose, USA) were reconstituted to 10–40 mg/ml in DMSO and diluted to working stock solutions of 4 mg/ml in PBS as described previously [15].

### *Ex vivo* IFN-γ ELISPOT assay

Freshly isolated PBMCs were used in an IFN-γ ELISPOT assay as described previously [15, 17]. Pre-wetted ELISPOT plate membranes (S5EJ044I10; Merck Millipore) were coated overnight at 4°C with anti-IFN-γ antibody (10 μg/ml in PBS; clone 1-D1K; Mabtech), washed with PBS and blocked with R10 (RPMI 1460 supplemented with 10% FBS, 2 mM L-glutamine, 10 mM HEPES, 1 mM sodium pyruvate and penicillin-streptomycin antibiotics; Sigma Aldrich) for 1 hour at 37°C. The PBMC were plated out at 2x10^5^ cells/well in 50 μl. For HIVconsv, triplicate well were used for six peptide pools P1-P6, 1.5 μg/ml per peptide. Six negative no-peptide control wells with R10 supplemented with 0.45% DMSO served as the background negative control. Cells cultured with 10 μg/ml PHA (Sigma Aldrich) or a pool of FEC (influenza virus, Epstein-Barr virus and human cytomegalovirus) known epitope peptides at 1 μg/ml each in triplicates were used as an internal positive control. Cell line NKL served as an external positive control for a consistent number of SFU independent of stimulation across different days and assay plates. The cells were incubated overnight at 37°C in 5% CO_2_. Spots were visualised using biotin-conjugated anti-IFN-γ mAb followed by alkaline phosphate-conjugated streptavidin (both from Mabtech) and the colour was developed using substrate BCIP/NBT^Plus^ (Mabtech). The reaction was stopped after 5 min by washing under the tap. The plates were air dried overnight and the spots were counted using an AID ELISpot Reader and version 5.0 software (AID GmbH). Median number of SFU in no-peptide wells was subtracted from test wells and the results were presented as median net SFU/10^6^ PBMC.

### Cultured IFN-γ ELISPOT assay

Frozen PBMC from volunteers were thawed and expanded *in vitro* with peptides plus IL-2 before being subjected to an IFN-γ ELISPOT assay. Short-term cell lines (STCL) were generated from all volunteers at the end of the pSG2.HIVconsv DNA vaccinations, the visit of ChAdV63.HIVconsv administration, by re-stimulation of the cells with each of the six peptide pools P1-P6 at 1.5 μg/ml per peptide in the presence of rhIL-7 at 25 ng/ml. The cells were maintained in culture and supplemented with recombinant human IL-2 at 100 IU/ml on days 3 and 7. After 10 days, the SCTL were washed, re-cultured without IL-2 for a minimum of 24 hours and then assayed for IFN-γ release in an ELISPOT assay as described above.

### Luminex assay

The Luminex assay is a multiplex bead array that measures, in a single sample of culture supernatant, the production by vaccine-elicited T cells of multiple cytokines and chemokines after restimulation of the cells by specific-peptides. The procedures were described previously [15]. Cryopreserved PBMCs from three time points (pre-immune, peak pSG2.HIVconsv DNA and peak ChAdV63.HIVconsv) were thawed and adjusted to 5 x 10^6^ cells/ml in R10 containing either a pool of all HIVconsv peptides at 1.5 μg/ml per peptide, *Staphylococcus* enterotoxin B (SEB; Sigma-Aldrich) at 1.0 μg/ml, or medium alone as a negative control and anti-CD28 and anti-CD49d mAbs both at 1 μg/ml in a final volume of 200 μl per well. The plates were incubated for 48 hours at 37°C, 5% CO_2_, and 150 μl of supernatant was removed from each well and stored at -80 ^o^C until use [18]. A human pre-mixed multi-analyte kit (Magnetic Luminex screening assay, R & D Systems Ltd) was used to measure the following analytes; IFN-γ, TNF-α, IL-2, IL-4, IL-6, IL-10, IL-13, IL-17A, SDF-1α (CXCL12), IP-10 (CXCL10), MIP-1α (CCL3), MIP-1β (CCL4), RANTES (CCL5), Granzyme B and MIG (CXCL9). The culture supernatants were diluted 1:1 and assayed in duplicate according to the kit instructions. Concentrations detected in unstimulated wells were used as background controls. The plate was read using Luminex 200 and XPONENT software.

### Polychromatic flow cytometry for memory subsets

PBMCs were thawed, incubated with 5 μl of the HLA-A*02:01/YQYMDDLV tetramer (NIH Tetramer Core Facility) conjugated to PE for 10 min at room temperature, followed by the addition of 100 μl of a mastermix of anti-membrane marker mAbs containing LIVE/DEAD fixable aqua stain (Molecular Probes, Invitrogen), CD3 ECD (Beckman Coulter), CD4 BV605 and CCR7 Pacific blue (Biolegend), CD8 Alexa Fluor 700, CD14 PE-Cy7, CD16 PE-Cy7, CD19 PE-Cy7, CD45RA APC, CD57 FITC, TIGIT PerCP-eFluor710, PD-1 APC-eFluor780 (eBiosciences) and CD27 Qdot 655 (Life Technologies). The cells were incubated at 4°C for a further 20 min, washed and fixed with 1% paraformaldehyde in PBS prior to running on an LSRII flow cytometer (Becton-Dickinson).

### Statistics

Statistical analyses were carried out using GraphPad Prism 6.0d. ELISPOT and Luminex results were assumed to be non-Gaussian in distribution, thus non-parametric tests were used throughout and medians (range) are shown. Area-under-curve (AUC) values were determined for the response of each volunteer within each group and analyzed using the Kruskal-Wallis test with post-hoc Dunn's Multiple Comparison Test. For comparison of the peak and breadth of responses to peptide pools, significance was determined by the Mann Whitney test. Two-tailed *P* values were used and *P* values of less than 0.05 were considered statistically significant.

## Results

### Safety and tolerability

All administrations of experimental vaccines and intravenous infusions of CPHPC were well tolerated. There were no serious adverse events. There were comparable numbers of non-serious adverse events adverse events in the CPHPC and placebo groups (Table 1).

**Table 1:** Non-serious adverse events.

Disorder	SAE	CPHPC	Placebo
Cardiac	Hypertension	1/20 (5.00%)	0/20 (0.00%)
Blood & lymphatics	Enlarged lymph node in neck	0/20 (0.00%)	1/20 (5.0%)
General	Headache	4/20 (20.00%)	7/20 (35.00%)
	Fatigue	2/20 (10.00%)	5/20 (25.00%)
	Insomnia	1/20 (5.00%)	1/20 (5.00%)
	Pain	1/20 (5.00%)	0/20 (0.00%)
	Dizziness	0/20 (0.00%)	1/20 (5.00%)
	Fever	0/20 (0.00%)	2/20 (10.00%)
	Rigors	0/20 (0.00%)	2/20 (10.00%)
Gastrointestinal	Diarrhoea	1/20 (5.00%)	0/20 (0.00%)
	Dysphasia	2/20 (10.00%)	1/20 (5.00%)
	Nausea	1/20 (5.00%)	2/20 (10.00%)
	Vomiting	0/20 (0.00%)	1/20 (5.00%)
	Anorexia	0/20 (0.00%)	1/20 (5.00%)
Renal & urinary	Abnormal odour	0/20 (0.00%)	1/20 (5.00%)
Skin & subcutaneous	Rash	2/20 (10.00%)	2/20 (10.00%)
Musculoskeletal	Injection site reaction	9/20 (45.00%)	12/20 (60.00%)
	Myalgia	7/20 (35.00%)	8/20 (40.00%)
	Neck stiffness	0/20 (0.00%)	1/20 (5.00%)
Infections & infestations	Coryzal symptoms	0/20 (0.00%)	2/20 (10.00%)
**Total**	**All**	**13/20 (65.00%)**	**16/20 (80.00%)**

### SAP depletion

At the time when the DNA vaccine was administered, the serum SAP concentration, measured by electroimmunoassay [19], was always below the 1 mg/l lower limit of detection in all the CPHPC recipients. All other serum samples from these subjects and from the recipients of the normal saline placebo infusion, contained SAP concentrations within the reference range [19]. The overall range of serum concentration of SAP in all subjects in the study when not receiving CPHPC was 15–65 mg/l, with mean ± SD of 31 ± 5 mg/l in the placebo group and 31 ± 8 mg/l in the CPHPC group.

### SAP depletion did not produce a statistically significant increase in T-cell induction

**Fig 3:**
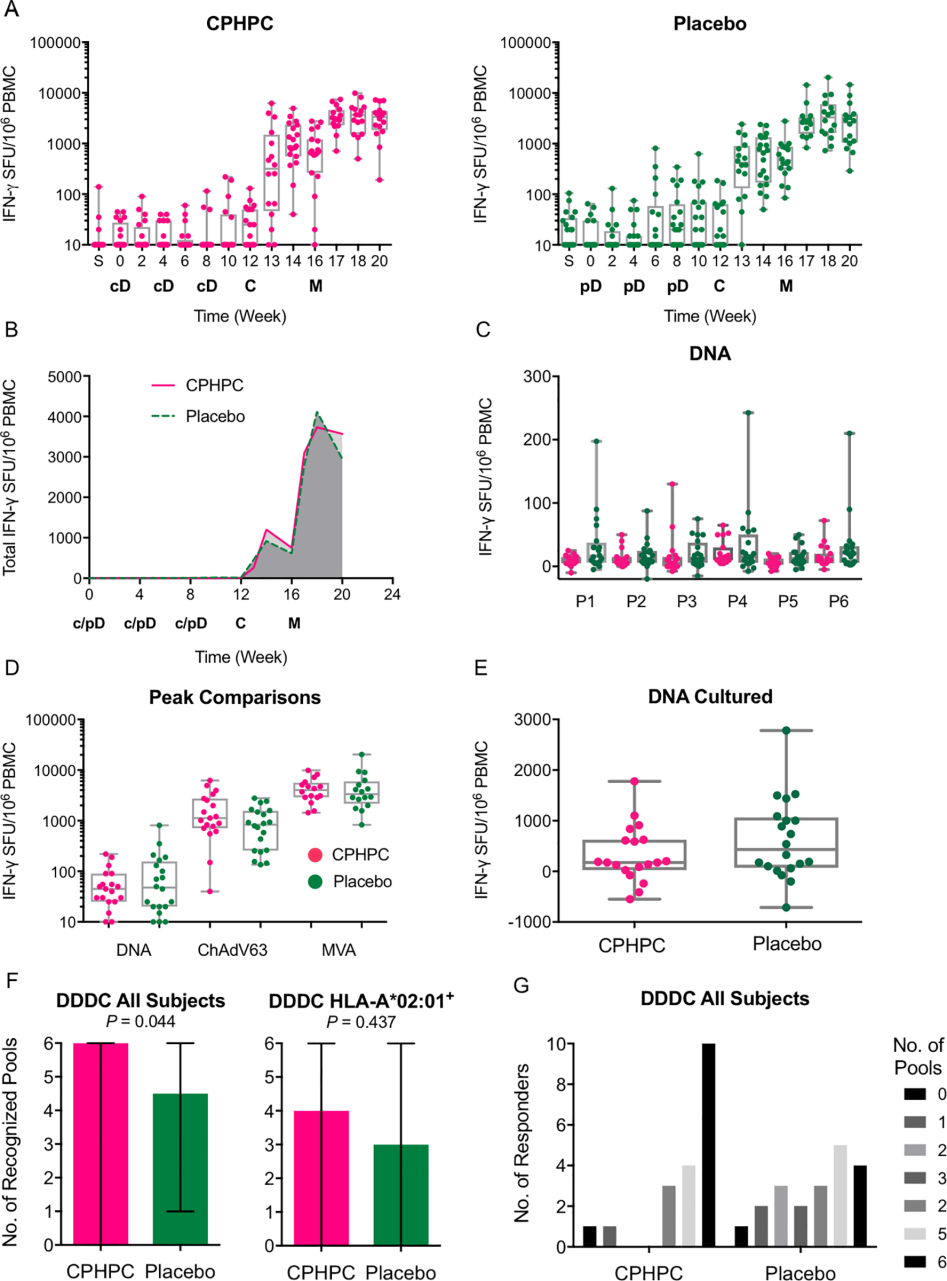
DNA priming of human T-cell responses with and without SAP depletion. Volunteers received heterologous vaccine regimens, which included priming with DNA either preceded or not by depletion of SAP. The vaccines were as follows: cD–pSG2.HIVconsv DNA after a 24-hour infusion of SAP-depleting CPHPC and additional 2-hour infusion after DNA administration (pink); pD–pSG2.HIVconsv DNA with 26-hour infusion of saline as placebo (green); C–simian adenovirus ChAdV63.HIVconsv; and M–poxvirus MVA.HIVconsv. All vaccines were delivered by intramuscular needle injection. Vaccine-induced T cells were enumerated in a fresh *ex vivo* (A-D and F) or following a 10-day expansion (E) IFN-γ ELISPOTs assay using 6 peptide pools P1-P6 spanning the HIVconsv immunogen. (A) Total frequencies were calculated as the sum of T cells responding to 6 pools of peptides P1-P6. The times of vaccine administration are indicated below the graphs. (B) Comparison of AUC using group median T-cell frequencies for each time point in the CPHPC (n = 13) and placebo (n = 13) groups. Only volunteers with full a data set were used. (C) Peak responses to individual pools P1-P6 elicited by plasmid DNA (week 0–8) are shown. (D) Peak T-cell frequencies induced following plasmid pSG2.HIVconsv DNA (week 0–8), ChAdV63.HIVconsv (week 8–12) and MVA.HIVconsv (week 16–20). (E) The proliferative capacity determined as the frequencies of HIVconsv-specific T cells following a 10-day restimulation of samples from week 12. (F) Breadth of induced T-cell responses defined as the number of recognized peptides pools (out of 6) following the ChAdV63.HIVconsv boost, whereby breadth for all (left) and HLA-A*02:01-positive subjects receiving CPHPC (n = 11) or placebo (n = 9) (right) are shown. Data are presented as median with range. (G) Bars depict the number of responders to 0–6 peptide pools P1-P6. The median, interquartile and total range, and individual values are plotted for each visit (A, C, D, and E).

An *ex vivo* IFN-γ ELISPOT assay on freshly isolated PBMCs was employed for detection of any effects that the SAP depletion could have on the efficiency of DNA vaccine priming and subsequent boosts with virus-vectored vaccines. One hundred and ninety nine 15-mer peptides overlapping by 11 amino acids (15/11) spanning the HIVconsv protein were arranged into six peptide pools P1-P6 (Fig 2A) and used to enumerate HIVconsv-specific T cells to assess the overall magnitude (as the sum of the 6 pool frequencies) and breadth (number of recognized pools) of the vaccine-elicited responses. Of the total of 520 samples tested, 11 or 2.1% failed the quality control. The overall kinetics of the elicited responses throughout the vaccination and follow-up periods for the SAP-depleted and untreated subjects showed very similar patterns (Fig 3A). This observation was confirmed by the area under curve (AUC) analysis of 13 volunteers in each group, for whom a complete data set was available. AUC was carried out over the entire time of the study and following each vaccine modality separately: the respective median AUCs for the CPHPC and placebo groups were 15 and 80 for pSG2.HIVconsv DNA (week 0–12), 2520 and 2355 for ChAdV63.HIVconsv (week 0–16), and 15460 and 14780 for MVA.HIVconsv (week 0–20) (Fig 3B). Following the DNA vaccinations with and without SAP depletion, we also failed to observe any differences for the six individual pools of the HIVconsv peptides (Fig 3C). Next we evaluated the peak IFN-γ ELISPOT assay frequencies of HIVconsv-specific T cells after each vaccine modality administration and, again, for the CPHPC and placebo groups, the respective median (range) frequencies were 45 (5–220) and 48 (0–810) induced by pSG2.HIVconsv DNA, 1130 (40–6215) and 835 (135–2785) induced by ChAdV63.HIVconsv, and 4020 (1440–9870) and 3345 (830–20305) following MVA.HIVconsv, which were not statistically separable (Fig 3D). We also assessed whether or not the SAP-depleted DNA priming affected the proliferative capacity of the induced T cells by enumerating the HIVconsv-specific T cells following a 10-day peptide pool-expansion cultured pre-immune and week 9 PBMCs. Once again, SAP depletion did not translate into increased proliferative capacity of HIVconsv-specific T cells (Fig 3E). Finally, we analysed the number of pools that individuals were able to mount T-cell responses against in parallel and found significantly broader responses after the ChAdV63.HIVconsv vaccination for the SAP-depleted group, whereby the SAP-depleted and placebo vaccine recipients responded to median (range) of 6 (0–6) and 4.5 (1–6) peptide pools (*P* = 0.044 in unpaired Mann-Whitney test) (Fig 3F) and 10 vs 4 volunteers responded to 6 pools, respectively (Fig 3G). Overall, following depletion of the SAP from human blood prior to intramuscular DNA administration, we were unable to detect improved induction of T-cell responses with the exception of significantly broader responses at only one point of the vaccination protocol, directly after the subjects had received DDDC.

### SAP depletion did not affect the vaccine-elicited T-cell functionality or memory subtypes

**Fig 4:**
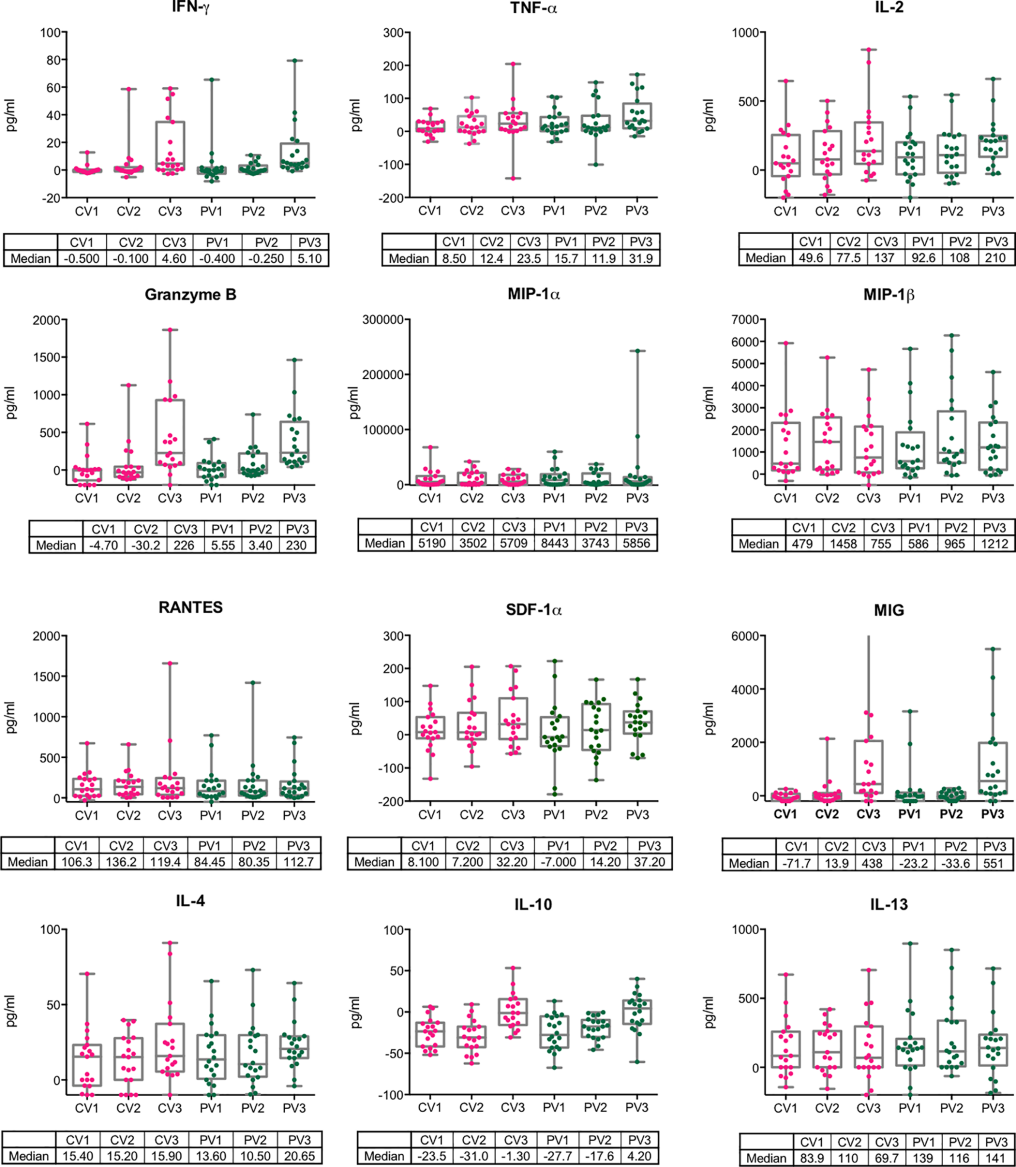
Functionality of vaccine-induced human T cells primed using plasmid DNA in the presence or absence of SAP. The functionality of T cells elicited with (pink) and without (green) CPHPC treatment prior to the DNA administration was assessed in the Luminex assay measuring the concentration of secreted signalling molecules into the culture supernatant by volunteers' PBMC following a 48-hour HIVconsv peptide restimulation. The functionality of specific T cells was determined after pSG2.HIVconsv DNA (CV1 and PV1), ChAdV63.HIVconsv (CV2 and PV2) and MVA.HIVconsv (CV3 and PV3) administration. At no point and for no measured cytokine were the values statistically separable between the CPHPC and placebo recipients (Mann-Witney test).

**Fig 5:**
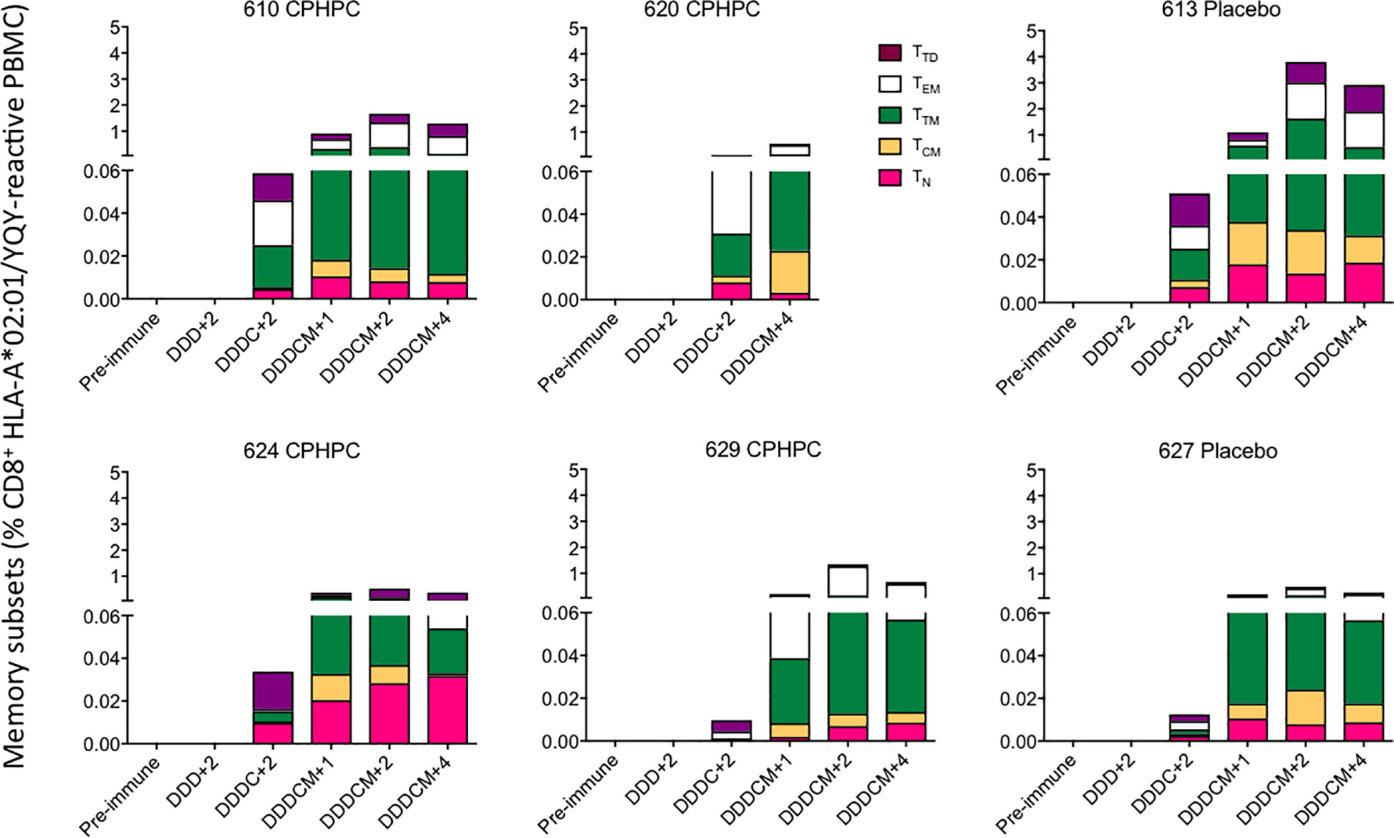
HLA-A*02:01/YQY tetramer-aided analysis of human CD8^+^ T-cell memory subsets. For six HLA-A*02:01-positive vaccine recipients, frozen and thawed PBMCs from indicated time point were analyzed for memory subsets defined as T_N_−naïve T cells (CD45RA^hi^CCR7^hi^CD27^hi^). T_CM_—central memory (CD45RA^lo^CCR7^hi^CD27^hi^), T_TM_—transitional memory (CD45RA^lo^CCR7^lo^CD27^hi^), T_EM_—effector memory (CD45RA^lo^CCR7^lo^CD27^lo^), and T_TD_—terminally differentiated (CD45RA^hi^CCR7^lo^CD27^lo^). See S1 Fig for the gating strategy. D–pSG2.HIVconsv DNA; C–ChAdV63.HIVconsv; M–MVA.HIVconsv. The number next to the regimen indicates weeks after administration of the last vaccine.

T cells can only contribute to the body’s defence if they exert their effector functions effectively and in a timely fashion. To assess any possible effects of the CPHPC treatment prior to DNA priming on the functionality of the HIVconsv-vaccine induced cellular responses, volunteers’ cryopreserved and thawed PBMCs were incubated with peptide pools P1-P6 for 48 hours and the tissue culture supernatants were analyzed using a multiplex bead array quantifying production of 15 cytokines and chemokines. These measurements were carried out for pre-immune, peak DNA and peak ChAdV63.HIVconsv samples. Overall, varied levels of analytes were detected, but the two trial Groups could not be separated (Fig 4). Six individuals, 4 in the CPHPC and 2 in the placebo groups, had HLA-A*02:01-positive tissue type, which allowed us to carry out a highly sensitive evaluation of memory subtypes of their CD8^+^ T cells recognizing HIV-1 Pol-derived epitope YQYMDDLYV using HLA-A*02:01/YQYMDDLYV peptide tetramers. In a polychromatic flow analysis, all six vaccine recipients developed a tetramer-reactive CD8^+^ T cells, which were structured into naïve (CD45RA^hi^CCR7^hi^CD27^hi^), central memory (CD45RA^lo^CCR7^hi^CD27^hi^), transitional memory (CD45RA^lo^CCR7^lo^CD27^hi^), effector memory (CD45RA^lo^CCR7^lo^CD27^lo^) and terminally differentiated (CD45RA^hi^CCR7^lo^CD27^lo^) T cells. No tetramer-reactive populations stained for the programmed cell death protein 1 (PD-1)/CD279 and T-cell immune receptor with Ig and ITIM domains (TIGIT) inhibitory markers. Again, we were unable to detect any outstanding differences between the SAP-depleted and placebo groups (Fig 5). Thus, depletion of circulating SAP prior to intramuscular needle administration of plasmid DNA, had no obvious quantitative or qualitative effect on priming of transgene-specific T cells nor did it influence subsequent boosts with simian adenovirus- or poxvirus MVA-vectored vaccines.

## Discussion

Enhancement of the immunogenicity of DNA vaccines in humans, making them effective in clinical practice, is highly desirable and there is compelling experimental animal evidence that depletion of human SAP might be helpful. Availability of the experimental SAP-depleting drug, CPHPC (now known by its recently awarded WHO International Non-Proprietary Name, miridesap) at University College London [5], and of experimental GMP vaccines, including one vectored by plasmid DNA [8], in Oxford University, uniquely enabled us to test this idea in humans. Infusion of CPHPC for 24 hours before each injection of the DNA vaccine reduced circulating SAP, as expected, to less than 1 mg/l. Multiple parameters of DNA vaccine-primed T cells were compared in the SAP-depleted and control groups: kinetics of appearance, frequency at peak, proliferative capacity, ability to produce intercellular signalling molecules and structure of memory subtypes. However, only the breadth of response, that is the number of different epitopes that T cells as a population recognize at any one time, reached statistical significance (*P* = 0.044) favouring the SAP-depleted recipients. This was observed only after the DNA prime-simian adenovirus boost regimen (DDDC), but not after DNAalone (DDD), and it was no longer detected after the MVA boost (DDDCM).

After vaccinating the first 7 volunteers with DDDC, we amended the immunization protocol by adding another rather strongly boosting vaccine modality MVA in the hope that any minor differences achieved during the DNA priming phase would be amplified. We have learnt since and here that a strongly boosting vaccine may have the opposite effect; rather than amplifying differences, it blurs them and masks them [20]. For that reason, we focused our search for effects on the vaccine-elicited T-cell responses on the period after DDD priming and before the simian adenovirus boost. Nevertheless, neither cultured IFN-γ ELISPOT assays nor profiling of functional molecules produced following a cognate peptide stimulation *in vitro* yielded any statistically separable results between the CPHPC- and placebo-treated groups.

We conclude that depletion of SAP with the regimen adopted here did not appreciably improve the immunogenicity of the naked DNA encoding the HIVconsv antigen. Our hypothesis may thus be wrong. However, the strong rationale provided by the experimental animal findings, that created the hypothesis, still remains and the present suggestion of a possible effect on breadth of immune response hints that it may be valid. There are several possible reasons for this marginal outcome. We excluded one possibility, namely that SAP does not bind the particular DNA immunogen we used, but the contrary possibility remains: the inhibitory potency of SAP may be so great that even the trace amount of residual SAP after CPHPC infusion was sufficient to block DNA immunogenicity. We may thus have used insufficient CPHPC to achieve our desired objective. Given the potential value to human health of efficacious DNA vaccines, and the possible development of an oral dosing form of CPHPC that would make practical SAP depletion a viable proposition, further clinical studies with more effective SAP depletion ahead of DNA vaccination would be worthwhile. In the present trial, CPHPC was infused for just 24 hours before DNA injection and continued for only 2 hours thereafter, based on logistical feasibility in this volunteer study. While circulating SAP was profoundly depleted, we do not know whether SAP was adequately removed from the extracellular compartment of the tissues, specifically the intramuscular sites of DNA injection. The binding of SAP to DNA is very avid and we do not know how much SAP may be sufficient to suppress the capacity of the DNA to transfect cells and trigger expression of the encoded protein immunogen. Investigation of the effects of SAP depletion on immunogenicity of DNA vaccines delivering different immunogens, if they are available, will also be of considerable interest.

Meanwhile, the experimental vaccines that were employed in the HIV-CORE 003 trial were the first generation of HIV-1 T-cell vaccines [7], which focuses elicited T-cell responses on highly functional conserved regions of the HIV-1 proteome common to most global variants, which are the most vulnerable parts of HIV-1 [6]. These vaccines have now been used in 8 clinical trials in UK, Europe and Africa. They have proven the concept that, in-natural-infection subdominant protein epitopes can be taken out of the context of the whole proteins/virus and delivered by an in-human potent regimen such as the simian adenovirus-MVA sequential administration to induce robust HIV-1-specific T-cell responses. These can inhibit *in vitro* replication of viruses of 4 major global clades [8, 9, 11, 21] and, in HIV-1-positive patients, provided a preliminary signal of controlling HIV-1 viremia after pausing antiretroviral treatment [12]. As we demonstrated previously, having induced robust CD8^+^ T-cells response in the volunteers of this trial provides a further opportunity to identify novel subdominant, but potentially protective HIV-1 epitopes [22]. Accumulation of knowledge about protective epitopes may in turn help predict early success or failure of future candidate T-cell vaccines against HIV-1.

## Supporting information

S1 Table HIV-CORE 003 ex vivo ELISPOT data. (PDF)

S2 Table HIV-CORE 003 cultured ELISPOT data. (PDF)

S3 Table HIV-CORE 003 luminex data. (PDF)

S1 Fig Gating strategy for tetramer-reactive T-cell analysis. (PDF)

S1 Text CONSORT 2010 checklist. (PDF)

S2 Text HIV-CORE 003 clinical protocol version 2.2. (PDF)

S3 Text HIV-CORE 003 clinical registry safety. (PDF)

S4 Text HIV-CORE 003 ELISPOT statistical report. (PDF)
