# Supplementary material for: Randomized phase I trial HIV-CORE 003: Depletion of serum amyloid P component and immunogenicity of DNA vaccination against HIV-1
Source: PLoS One. 2018 May 17;13(5):e0197299. doi: 10.1371/journal.pone.0197299 (PMC5957335; doi:10.1371/journal.pone.0197299)
Supplement: S1 Table — (PDF) [file pone.0197299.s001.pdf]

Group 1 CPHPC

Vacc		DNA		DNA		DNA		ChAdV63				
**Week**	Screen	0	2	4	6	8	10	12	14	16	18	20
**Visit**	**1**	2	3	4	5	6	7	8	9	10	11	12
**601**	-45	-115	-55	-20	-35	45	-230	165	150	435	190	180
**605**	45	-30	15	-35	-40	-30	10	-105	1590	840	560	265
**606**	35	40	-35	-25	15	20	-10	20	90	260	175	125
**607**	20	65	25	50	350	190	175	65	240	ND	ND	ND

Vacc		DNA		DNA		DNA		ChAdV63			MVA			
**Week**	Screen	0	2	4	6	8	10	12	13	14	16	17	18	20
**Visit**	**1**	2a	3	4a	5	6a	7	8	9	10	11	12	13	14
**609**	0	-5	50	Fail	0	35	35	-47.5	45	105	145	830	730	290
**613**	45	-40	-85	10	-25	5	-10	-55	1490	935	1000	6370	9400	8920
**614**	25	5	-25	-70	-20	25	-155	15	80	155	85	1430	4260	2440
**615**	-70	5	0	-70	-5	-45	20	-25	455	2385	2785	14410	20305	14575
**616**	-2.5	-20	-5	15	-20	190	180	65	255	210	65	1300	1740	670
**619**	-100	55	-155	-240	-220	-70	-120	-65	-295	920	410	3520	3740	1420
**621**	105	-5	25	-20	100	10	70	15	790	475	330	2880	1290	800
**622**	-110	Fail	130	-95	-70	150	55	60	90	50	135	2640	1830	1010
**627**	0	-35	0	-35	20	5	20	5	380	740	405	3530	9010	6280
**628**	20	-15	0	75	45	20	70	70	940	785	495	2690	2850	2950
**631**	-20	30	0	15	ND	25	5	-5	580	290	325	2000	880	1130
**632**	-35	-35	-70	-110	-185	-130	-110	-35	375	1325	990	2710	6300	3860
**634**	75	-10	20	50	210	0	145	0	2435	1340	615	2430	4120	3310
**635**	30	30	5	25	5	20	35	45	1670	1445	760	2870	2315	2820
**637**	20	-60	-130	15	810	345	630	185	515	655	880	2800	5090	3670
**638**	-100	-110	-340	-105	40	70	-40	55	285	2285	870	1400	1570	1170

Gruup 2 placebo

Vacc		DNA		DNA		DNA		ChAdV63				
**Week**	Screen	0	2	4	6	8	10	12	14	16	18	20
**Visit**	**1**	2	3	4	5	6	7	8	9	10	11	12
**602**	-30	-85	-25	30	-55	-115	-5	75	150	-5	-80	40
**603**	140	35	-25	40	60	115	220	50	615	90	120	175
**608**	-35	-105	-195	-110	-45	-60	-30	25	1480	590	415	355

Vacc		DNA		DNA		DNA		ChAdV63			MVA			
**Week**	Screen	0	2	4	6	8	10	12	13	14	16	17	18	20
**Visit**	**1**	2a	3	4a	5	6a	7	8	9	10	11	12	13	14
610	5	30	-45	30	-55	-90	45	25	40	830	1130	3010	3670	7210
611	5	0	-10	-50	-35	10	-25	55	6215	2330	2765	2510	3710	3570
612	-10	45	40	10	-15	55	ND	-25	20	830	655	3110	1310	840
617	-105	-140	90	-60	15	-20	-65	-45	65	890	610	2900	2620	2290
618	-40	10	-45	-40	-20	-30	-15	30	250	2740	2130	6800	9870	7010
**620**	0	-45	-20	0	40	-5	0	15	550	370	265	4740	ND	4480
623	-180	-40	30	-55	-80	0	-45	130	380	555	710	3100	2770	2620
624	-25	-10	15	10	30	10	0	10	1010	415	225	3240	4745	3065
625	10	20	5	0	0	-10	-40	-5	0	40	20	1440	500	190
**626**	20	45	-10	-95	-115	-65	90	-75	1655	1990	750	710	1530	1550
629	0	10	-30	30	-10	-20	5	-35	65	775	660	3610	4330	3970
630	5	-10	35	-35	-10	5	5	-5	50	3345	1340	640	2200	
633	10	15	25	15	0	-5	-10	-15	470	1200	1210	4100	5090	3730
**636**	-25	-5	-20	40	15	50	190	60	3960	3420	2650	5830	5230	4040
639	-10	-50	-10	5	0	-30	-15	-35	255	4950	1925	7470	8200	6830
641	FAIL	-75	50	-30	-25	10	35	50	-10	2540	710	2240	1500	1720

	**Peak**
	DNA.HIVconsv
	ChAd63.HIVconsv
	MVA.HIVconsv
