# Supplementary material for: Randomized phase I trial HIV-CORE 003: Depletion of serum amyloid P component and immunogenicity of DNA vaccination against HIV-1
Source: PLoS One. 2018 May 17;13(5):e0197299. doi: 10.1371/journal.pone.0197299 (PMC5957335; doi:10.1371/journal.pone.0197299)
Supplement: S2 Table — (PDF) [file pone.0197299.s002.pdf]

Less Bg/Per Million

CPHPC	PTID	Visit	Pool 1	Pool 2	Pool 3	Pool 4	Pool 5	Pool 6
	602	8	75	825	578.5	237.5	-112.5	175
	603	8	200	212.5	387.5	87.5	37.5	-12.5
	608	8	-62.5	100	-125	-162	-337	37.5
	610	7	-187.5	62.5	100	37.5	-150	-100
	611	8	137.5	337.5	350	87.5	25	162.5
	612	7	37.5	-50	12.5	0	0	175
	617	8	125	-25	0	-37.5	-62.5	-75
	618	8	25	-12.5	37.5	87.5	-25	-25
	620	8	-12.5	12.5	50	25	50	0
	623	8	50	37.5	112.5	0	-12.5	0
	624	8	137.5	62.5	37.5	225	100	275
	625	8	-12.5	-125	-137	-75	162	-225
	626	8	350	37.5	75	-37.5	175	-12.5
	629	8	-50	12.5	50	87.5	12.5	25
	630	8	50	37.5	37.5	12.5	-12.5	50
	633	8	62.5	37.5	37.5	12.5	12.5	37.5
	636	8	112.5	25	250	37.5	50	150
	639	8	425	0	25	37.5	12.5	112.5
	641	8	12.5	-25	-112.5	162.5	37.5	-50

Less Bg/Per Million

Placebo	PTID	Visit	Pool 1	Pool 2	Pool 3	Pool 4	Pool 5	Pool 6
	601	8	312.5	137.5	287.5	500	62.5	137.5
	605	8	175	187	175	50	162.5	337.5
	606	8	25	-12.5	25	12.5	50	75
	607	8	112.5	62.5	150	87.5	100	225
	609	7	-137.5	1212	525	-62.5	75	-112.5
	613	8	25	-25	-50	-25	-100	0
	614	8	-37.5	-225	-150	-137.5	-125	-37.5
	615	8	-50	-75	337.5	-62.5	-50	87.5
	616	8	212.5	0	287.5	137.5	87.5	162.5
	619	8	12.5	50	37.5	-25	12.5	62.5
	621	8	62.5	12.5	0	12.5	12.5	-37.5
	622	8	0	-37.5	0	25	50	62.5
	627	8	337.5	550	75	50	-25	12.5
	628	8	12.5	-25	50	112.5	150	237.5
	631	8	12.5	-87.5	0	75	0	12.5
	632	8	75	-212	1025	112.5	312.5	212.5
	634	8	75	37.5	62.5	100	75	-25
	635	8	262.5	12.5	275	225	75	150
	637	8	12.5	82.5	100	575	1112	900
	638	8	37.5	-87.5	-12.5	-12.5	37.5	-37.5

Total	Ex Vivo
1778.5	-115
912.5	115
-549	-60
-237.5	-55
1100	10
175	-15
-75	-20
87.5	-30
125	-5
187.5	0
837.5	10
-412.5	-10
587.5	-65
137.5	-20
175	5
200	-5
625	50
612.5	-30
25	10

Total	Ex Vivo
1437.5	45
1087	-30
175	20
737.5	190
1499.5	35
-200	5
-712.5	25
187.5	-45
887.5	190
150	-70
62.5	10
100	150
1000	5
537.5	20
12.5	25
1525.5	-130
325	0
1000	20
2782	345
-75	70
