# Supplementary material for: Randomized phase I trial HIV-CORE 003: Depletion of serum amyloid P component and immunogenicity of DNA vaccination against HIV-1
Source: PLoS One. 2018 May 17;13(5):e0197299. doi: 10.1371/journal.pone.0197299 (PMC5957335; doi:10.1371/journal.pone.0197299)
Supplement: S3 Table — (PDF) [file pone.0197299.s003.pdf]

All concentrations (pg/ml) have the background from unstimulated cells removed

V1: Preimmune V2: Peak DNA V3: Peak ChAd63

TNFα CPHPC	V1	V2	V3
602	1.3	-2.6	-1.0
603	-21.4	22.8	55.1
608	-8.5	2.6	42.7
610	-10.9	63.2	98.0
611	28.9	35.2	30.2
612	-30.9	-37.5	-142.1
617	4.0	12.4	7.9
618	24.9	-28.8	40.4
620	3.4	0.5	-0.5
623	25.6	60.8	55.0
624	3.9	10.6	14.9
625	52.0	24.5	12.6
626	0.8	1.2	7.3
629	24.2	-6.8	1.6
630	19.6	15.1	23.5
633	8.5	-0.9	4.9
636	30.0	102.6	68.4
639	69.0	46.1	204.4
641	30.3	55.2	45.5

TNFα Placebo	V1	V2
601	-0.5	-1.1
605	-31.5	48.6
606	4.1	-10.6
607	23.6	16.2
609	9.5	11.9
613	105.1	103.5
614	102.3	109.7
615	43.1	7.5
616	3.7	6.3
619	20.1	1.6
621	22.6	11.8
622	16.5	17.6
627	-4.6	-100.5
628	43.8	45.6
631	-23.6	8.7
632	11.2	-4.5
634	14.9	148.5
635	53.5	3.9
637	73.0	122.7
638	-5.3	24.1

SDF-1α CPHPC	V1	V2	V3
602	-5.7	-13.2	-8.7
603	10.6	7.2	32.2
608	0.0	2.4	21.7
610	-132.1	-50.2	138.1
611	-7.7	-26.3	59.5
612	8.1	0.0	-40.0
617	-10.5	49.4	-12.6
618	53.6	-32.7	143.6
620	-30.8	-8.7	-36.0
623	24.3	66.4	193.5
624	39.5	61.6	42.9
625	93.9	104.9	24.3
626	-60.3	22.9	-53.1
629	78.1	-95.5	-56.9
630	60.6	112.2	46.0
633	-47.3	20.8	39.8
636	6.1	205.4	110.3
639	147.7	3.3	206.8
641	24.7	150.0	6.2

SDF-1α Placebo	V1	V2
601	-7.2	-69.1
605	-41.7	13.0
606	-17.5	-32.7
607	19.5	-8.6
609	-5.2	37.7
613	56.1	97.4
614	176.8	166.4
615	66.4	-48.6
616	-30.7	15.4
619	34.7	-53.4
621	-12.5	8.2
622	43.4	82.6
627	-6.8	-136.4
628	81.2	96.1
631	-179.4	-36.2
632	-21.8	-86.6
634	-46.3	75.7
635	222.2	107.1
637	-35.5	55.8
638	-161.5	98.7

IP-10 CPHPC	V1	V2	V3
602			
603	-515.8	-20.3	234.8
608	-859.7		173.0
610			
611			

IP-10 Placebo	V1	V2
601		
605		
606		
607	-225.7	1009.8
609	-18.5	-691.8

612			
617			
618			
620			
623			
624			
625			
626			
629			
630	-97.7	-422.9	1041.6
633			
636	-586.3	-387.4	1471.9
639	-16.6	-642.2	7601.3
641	-280.7	18307.2	26411.3

613		
614	-252.4	-714.3
615	-742.5	-724.6
616		
619		
621		
622		
627	-791.8	960.2
628	-445.8	-1081.5
631		
632	-729.6	-423.2
634	462.9	
635	121.9	-0.7
637		-598.5
638		-124.5

IL-10 CPHPC	V1	V2	V3
602	-44.1	-53.7	-13.4
603	-32.2	1.3	10.3
608	-28.2	-19.0	6.6
610	-22.7	-41.4	-7.1
611	-25.5	-32.3	-22.3
612	-13.0	-42.6	-30.7
617	-47.4	-52.0	-9.9
618	-41.7	-4.7	33.9
620	-18.3	-62.2	-25.5
623	-47.5	-34.2	-1.3
624	-23.5	-21.7	-1.0
625	4.5	-31.0	-15.7
626	-52.0	-30.0	7.0
629	-10.5	-40.6	-17.2
630	-0.1	-17.7	16.7
633	-40.1	-20.7	15.6
636	-17.6	-10.7	22.9
639	6.4	-54.7	53.2
641	-14.1	9.2	-6.9

IL-10 Placebo	V1	V2
601	-15.5	-20.4
605	-47.4	-9.0
606	-44.9	-30.8
607	-6.9	-1.5
609	-3.2	-31.2
613	-36.3	-21.8
614	-4.8	-17.5
615	-32.9	-41.3
616	-13.2	-3.5
619	-42.5	-45.7
621	-29.9	-11.5
622	-4.5	-12.1
627	-31.5	-28.9
628	-17.3	-33.0
631	-50.9	-20.8
632	-25.5	-17.6
634	-0.8	-11.6
635	13.2	-0.4
637	-43.3	-16.2
638	-67.4	-4.1

IFNγ CPHPC	V1	V2	V3
602	-1.1	-1.0	0.4
603	-0.5	4.1	37.7
608	-1.7	1.3	7.6
610	-1.4	7.4	-2.5
611	-0.1	2.0	3.8
612	-2.0	-1.9	34.7
617	-0.3	-0.9	7.6
618	-0.5	0.7	20.2
620	-0.6	-1.4	-2.7
623	-1.7	-0.9	0.7
624	0.2	0.6	3.1
625	3.7	0.0	0.2
626	-1.4	-1.3	2.8

IFNγ Placebo	V1	V2
601	-2.8	-2.7
605	-4.6	-0.5
606	-0.6	-2.3
607	-0.9	3.8
609	-0.1	-1.5
613	-8819.1	-29053.9
614	1.4	-2.0
615	-0.8	-0.9
616	0.2	0.7
619	-0.2	-0.4
621	6.3	0.3
622	12.0	6.2
627	-8.1	-2.2

**629**	12.7	-0.4	-0.1
**630**	1.1	-0.1	4.6
**633**	-2.2	-0.5	11.9
**636**	0.4	8.6	51.6
**639**	0.0	-5.2	59.0
**641**	-0.2	58.6	54.9

**628**	1.9	1.1
**631**	-0.2	-0.1
**632**	-1.9	-0.8
**634**	65.3	5.0
**635**	1.9	1.8
**637**	-3.7	9.1
**638**	-5.7	-0.4

**MIP-1α CPHPC**	**V1**	**V2**	**V3**
**602**	129.4	166.2	11.9
**603**	707.2	3501.8	5709.0
**608**	453.1	768.5	3648.7
**610**	-59.9	41984.2	25659.3
**611**	358.7	21398.5	12941.3
**612**	10327.0	3491.4	8442.1
**617**	968.0	15348.7	18041.4
**618**	6651.4	55.5	1276.2
**620**	1126.4	300.3	109.3
**623**	15619.4	23495.3	28540.6
**624**	6173.2	3349.5	1585.5
**625**	23705.1	26064.6	2816.0
**626**	181.6	203.7	74.3
**629**	10971.9	91.1	190.5
**630**	28673.0	19371.0	18511.3
**633**	5190.1	394.1	164.0
**636**	2673.1	14714.2	10308.6
**639**	67903.1	33221.1	21043.8
**641**	23323.7	10957.4	11035.3

**MIP-1α Placebo**	**V1**	**V2**
**601**	13.3	-4.9
**605**	11109.8	5164.6
**606**	273.9	-6.2
**607**	49314.3	3908.2
**609**	1233.6	4345.8
**613**	8929.8	29120.3
**614**	28483.0	27982.8
**615**	59793.9	37132.3
**616**	726.2	949.1
**619**	14135.0	510.3
**621**	378.6	531.0
**622**	1637.3	3923.8
**627**	20276.0	3577.1
**628**	27809.1	28519.4
**631**	31.6	2319.6
**632**	15005.4	3160.1
**634**	14755.3	16228.9
**635**	7956.5	1351.7
**637**	2276.3	1714.1
**638**	137.3	21798.5

**RANTES CPHPC**	**V1**	**V2**	**V3**
**602**	-30.2	51.8	5.3
**603**	35.2	193.4	111.4
**608**	24.6	104.4	295.1
**610**	106.3	216.3	72.5
**611**	6.9	270.5	174.2
**612**	250.6	59.0	138.3
**617**	28.4	136.2	135.2
**618**	314.4	9.6	89.2
**620**	92.5	34.0	4.0
**623**	160.6	186.7	1659.3
**624**	87.7	39.8	38.1
**625**	298.3	660.1	119.4
**626**	13.7	1.7	5.1
**629**	234.7	45.2	55.0
**630**	218.6	204.1	181.9
**633**	162.9	61.7	10.5
**636**	22.9	213.3	244.4
**639**	673.7	342.6	705.1
**641**	230.8	329.5	256.2

**RANTES Placebo**	**V1**	**V2**
**601**	20.9	15.7
**605**	49.4	138.6
**606**	14.4	5.6
**607**	771.6	215.9
**609**	106.3	216.3
**613**	62.6	397.3
**614**	649.3	1420.8
**615**	214.5	72.5
**616**	9.9	34.1
**619**	278.7	89.8
**621**	42.1	60.9
**622**	16.8	28.6
**627**	147.0	7.9
**628**	221.5	255.9
**631**	-2.8	14.2
**632**	152.8	57.4
**634**	120.1	88.2
**635**	208.4	132.4
**637**	27.9	44.7
**638**	-57.1	292.1

MIP-1β CPHPC	V1	V2	V3
602	108.1	95.0	7.9
603	478.5	1495.1	2178.1
608	86.5	210.3	754.8
610	-301.7	2052.2	3394.5
611	468.8	2713.8	1821.5
612	338.6	540.3	590.3
617	270.8	1457.9	-991.8
618	2677.0	54.0	1098.3
620	121.5	104.1	-22.6
623	2694.3	1838.4	2153.1
624	286.0	347.6	76.4
625	977.8	2660.7	1256.1
626	170.7	286.8	246.0
629	1985.2	-15.3	71.5
630	2867.2	2269.8	-101.3
633	1867.8	264.9	449.5
636	1571.9	2894.2	2002.0
639	5919.1	5274.9	4725.5
641	2323.6	2566.8	2635.7

MIP-1β Placebo	V1	V2
601	-41.3	-60.2
605	-13.6	3337.2
606	262.6	-42.0
607	276.8	1128.1
609	533.8	1059.2
613	2361.5	4371.2
614	5663.5	6269.3
615	1322.7	627.2
616	353.0	429.4
619	1248.0	57.1
621	425.0	514.6
622	542.8	1027.6
627	629.9	529.8
628	4110.4	5589.9
631	-149.3	902.0
632	1245.8	671.6
634	1164.2	2947.0
635	3711.5	796.4
637	2077.4	1620.8
638	173.2	2519.6

IL-4 CPHPC	V1	V2	V3
602	-17.7	-10.5	3.7
603	15.4	28.1	24.9
608	0.0	7.7	25.9
610	-49.5	15.2	51.2
611	0.0	19.7	21.4
612	0.0	6.5	10.3
617	-3.8	7.4	3.2
618	27.7	-11.2	41.5
620	-4.1	-21.3	-12.9
623	17.3	17.3	91.0
624	3.8	15.9	5.5
625	16.8	36.9	11.8
626	-9.4	0.0	15.9
629	32.8	-24.3	3.8
630	23.2	27.7	24.0
633	21.5	0.0	13.5
636	19.1	39.7	37.3
639	70.4	25.4	83.7
641	37.2	38.9	7.7

IL-4 Placebo	V1	V2
601	3.2	-9.6
605	-2.6	31.0
606	6.6	-8.8
607	29.9	19.2
609	19.1	29.9
613	37.5	49.8
614	65.6	73.0
615	22.3	8.0
616	0.0	0.0
619	31.0	-4.7
621	-3.8	8.0
622	14.9	11.3
627	12.3	3.0
628	29.2	29.2
631	-24.5	6.4
632	9.7	1.8
634	24.2	34.3
635	42.7	9.7
637	9.0	21.9
638	-35.8	26.0

IL-2 CPHPC	V1	V2	V3
602	-155.7	-117.6	-26.6
603	37.4	189.6	222.8
608	-43.5	77.5	270.3
610	-397.2	46.6	345.0
611	18.7	175.1	137.2
612	99.2	-57.8	76.4
617	-62.1	57.4	60.9

IL-2 Placebo	V1	V2
601	-31.8	-46.9
605	-82.2	115.9
606	-27.4	-97.5
607	204.5	155.3
609	118.4	100.5
613	262.8	501.8
614	532.1	545.4

618	165.7	35.6	308.8
620	35.5	-179.6	-74.4
623	94.1	151.2	780.9
624	49.6	122.2	109.7
625	291.5	354.0	114.3
626	-178.4	-30.5	-14.4
629	254.1	-151.2	-41.6
630	326.6	282.0	205.6
633	-7.4	-10.2	45.6
636	68.1	309.5	384.1
639	645.1	501.7	872.6
641	270.2	417.5	422.2

615	163.1	57.3
616	-50.2	97.8
619	115.6	-93.1
621	69.0	27.5
622	188.6	241.1
627	16.7	-51.8
628	239.2	256.3
631	-210.2	23.3
632	69.5	-34.2
634	190.9	260.5
635	453.8	158.4
637	46.2	169.6
638	-105.0	255.3

**IL-13 CPHPC**	**V1**	**V2**	**V3**
**602**	-63.5	-63.5	0.0
**603**	51.9	239.6	319.2
**608**	0.0	-22.0	168.0
**610**	-75.6	262.7	460.6
**611**	119.7	302.3	152.8
**612**	0.0	109.7	86.7
**617**	-45.3	216.1	-168.6
**618**	468.4	0.0	229.2
**620**	0.0	65.3	-65.3
**623**	258.7	238.4	467.8
**624**	83.9	-55.4	0.0
**625**	222.9	317.9	0.0
**626**	-143.8	0.0	0.0
**629**	227.2	0.0	47.0
**630**	373.8	245.3	69.7
**633**	192.0	72.6	0.0
**636**	72.6	384.0	296.7
**639**	671.3	-155.5	704.9
**641**	290.2	420.3	-285.2

**IL-13 Placebo**	**V1**	**V2**
**601**	-11.6	0.0
**605**	-149.5	426.6
**606**	153.8	-63.5
**607**	142.8	173.0
**609**	109.7	0.0
**613**	389.9	506.0
**614**	896.8	850.2
**615**	124.1	38.5
**616**	0.0	83.7
**619**	216.1	0.0
**621**	55.4	110.8
**622**	171.0	120.6
**627**	143.8	30.9
**628**	479.4	719.7
**631**	0.0	0.0
**632**	176.4	145.2
**634**	126.0	327.0
**635**	413.1	72.6
**637**	135.0	327.0
**638**	-808.8	342.3

**MIG CPHPC**	**V1**	**V2**	**V3**
**602**	-50.3	-49.9	0.0
**603**	-12.6	84.0	488.7
**608**	-88.0	101.5	437.7
**610**	-97.4	38.6	27.4
**611**	-42.0	-384.5	432.9
**612**	-71.7	-140.6	1168.1
**617**	-87.2	-158.1	190.5
**618**	-227.0	529.6	12032.5
**620**	-1124.0	-875.2	-1796.4
**623**	-157.8	14.3	172.2
**624**	27.3	28.2	191.2
**625**	254.9	13.9	-224.1
**626**	-269.0	-737.2	3116.2
**629**	109.6	-84.5	107.4
**630**	119.5	-35.4	901.0

**MIG Placebo**	**V1**	**V2**
**601**	-317.0	-941.5
**605**	-277.5	-34.0
**606**	-314.4	-428.2
**607**	21.7	232.7
**609**	49.8	-111.7
**613**	-625.0	-1879.3
**614**	137.3	78.8
**615**	-31.4	-33.1
**616**	17.0	190.9
**619**	-16.1	-52.2
**621**	-1121.0	0.0
**622**	1939.9	131.1
**627**	-27.7	-1083.3
**628**	-29.0	-109.7
**631**	-18.7	-51.2

**633**	-367.0	-88.6	1259.0
**636**	-286.6	355.6	2057.4
**639**	178.1	164.2	3023.4
**641**	75.5	2136.4	2204.3

**632**	-43.8	-36.5
**634**	3158.4	282.6
**635**	200.2	71.4
**637**	-633.4	255.3
**638**	223.5	133.9

**Granzyme B CPHI**	**V1**	**V2**	**V3**
**602**	9.1	-25.1	71.0
**603**	-37.3	382.5	936.5
**608**	-132.3	-118.8	408.6
**610**	-272.0	246.9	-422.5
**611**	11.2	-59.7	193.7
**612**	-313.9	-76.3	459.7
**617**	-137.1	-102.9	143.3
**618**	9.1	44.5	374.9
**620**	-0.3	-106.5	-60.7
**623**	-53.5	-90.3	103.5
**624**	-76.2	-30.2	89.8
**625**	190.1	42.6	59.9
**626**	-391.5	-61.3	369.6
**629**	612.5	-9.7	41.2
**630**	339.2	31.9	1175.9
**633**	-232.9	-31.9	225.6
**636**	36.8	258.5	929.0
**639**	-0.7	-125.3	1861.2
**641**	-4.7	1126.2	980.1

**Granzyme B Place**	**V1**	**V2**
**601**	-88.5	8.0
**605**	-474.4	-72.2
**606**	12.2	-33.7
**607**	39.9	298.0
**609**	-123.6	77.9
**613**	65.2	242.8
**614**	120.6	16.8
**615**	-68.3	-67.4
**616**	17.9	46.3
**619**	-60.9	-8.4
**621**	51.3	-41.7
**622**	411.4	187.3
**627**	-150.1	-78.3
**628**	373.7	307.1
**631**	-90.9	-1.2
**632**	-1.1	-55.8
**634**	102.2	231.5
**635**	108.0	-19.4
**637**	-6.2	738.1
**638**	-304.8	-16.9

V3
25.0
129.8
1.1
30.4
12.5
172.2
92.2
61.4
8.5
-14.6
8.1
18.1
25.5
56.5
-2.8
36.5
59.0
33.4
133.1
144.5

V3
55.3
29.9
15.7
-1.9
71.8
19.9
109.8
53.4
67.0
-69.6
26.1
58.2
-58.5
-60.5
36.3
38.1
0.0
167.1
89.2
124.2

V3
-673.1
92.6

72.8
198.1

-267.8
406.8

2042.9
2283.9
1539.5
336.1
4586.9

V3
-10.2
7.5
-12.9
-2
11.5
-60.4
9.6
0.9
40.0
-19.9
-21.8
-26.7
-14.9
22.7
14.0
10.1
20.5
13.9
-10.9
30.7

V3
7.3
21.0
1.1
3.7
1.4
-31759.7
2.3
41.5
1.7
-0.7
2.6
4.1
6.3

36.4
5.1
5.1
79.2
10.5
4.1
13.7

**V3**
943.8
5627.4
316.1
8479.7
3067.7
31452.0
242642.3
10091.2
264.7
189.5
15.5
6084.1
16885.3
13719.4
26.8
7636.3
13742.5
4172.5
911.7
87594.3

**V3**
124.8
52.8
18.6
744.3
72.5
288.7
448.1
207.7
17.9
24.1
-5.0
67.2
116.3
132.0
5.9
159.8
109.0
178.2
56.0
676.5

V3
1242.3
2570.7
176.1
2245.8
774.3
3079.9
3242.5
705.3
255.9
-14.8
71.5
1280.6
1182.6
-59.7
84.5
1262.7
1036.2
2366.0
1732.9
4617.0

V3
18.3
14.7
9.2
27.2
24.2
27.0
53.4
28.2
19.1
0.0
-4.1
14.5
16.3
22.2
10.7
29.1
18.5
30.1
38.5
64.3

V3
151.6
153.5
32.9
332.7
214.9
124.6
505.3

252.0
230.7
-27.1
-21.9
87.5
146.6
236.7
8.7
208.0
212.0
292.2
234.7
660.9

V3
98.3
211.7
63.5
205.9
144.6
400.9
611.4
124.1
83.7
-167.5
-185.9
136.4
-102.8
-2.8
-84.1
197.5
270.1
207.6
245.4
715.6

V3
778.0
2137.3
41.5
95.5
75.2
-2063.2
171.4
909.8
1996.7
288.9
131.9
-371.9
85.1
1213.2
5494.3

760.2
4426.7
1937.8
342.1
3046.9

V3
673.0
541.9
111.1
310.2
143.8
409.7
90.6
1032.6
140.1
41.2
112.0
177.6
203.1
1461.3
64.7
94.2
720.4
257.2
684.1
496.3
