# Supplementary figures and images for: Randomized phase I trial HIV-CORE 003: Depletion of serum amyloid P component and immunogenicity of DNA vaccination against HIV-1
Source: PLoS One. 2018 May 17;13(5):e0197299. doi: 10.1371/journal.pone.0197299 (PMC5957335; doi:10.1371/journal.pone.0197299)

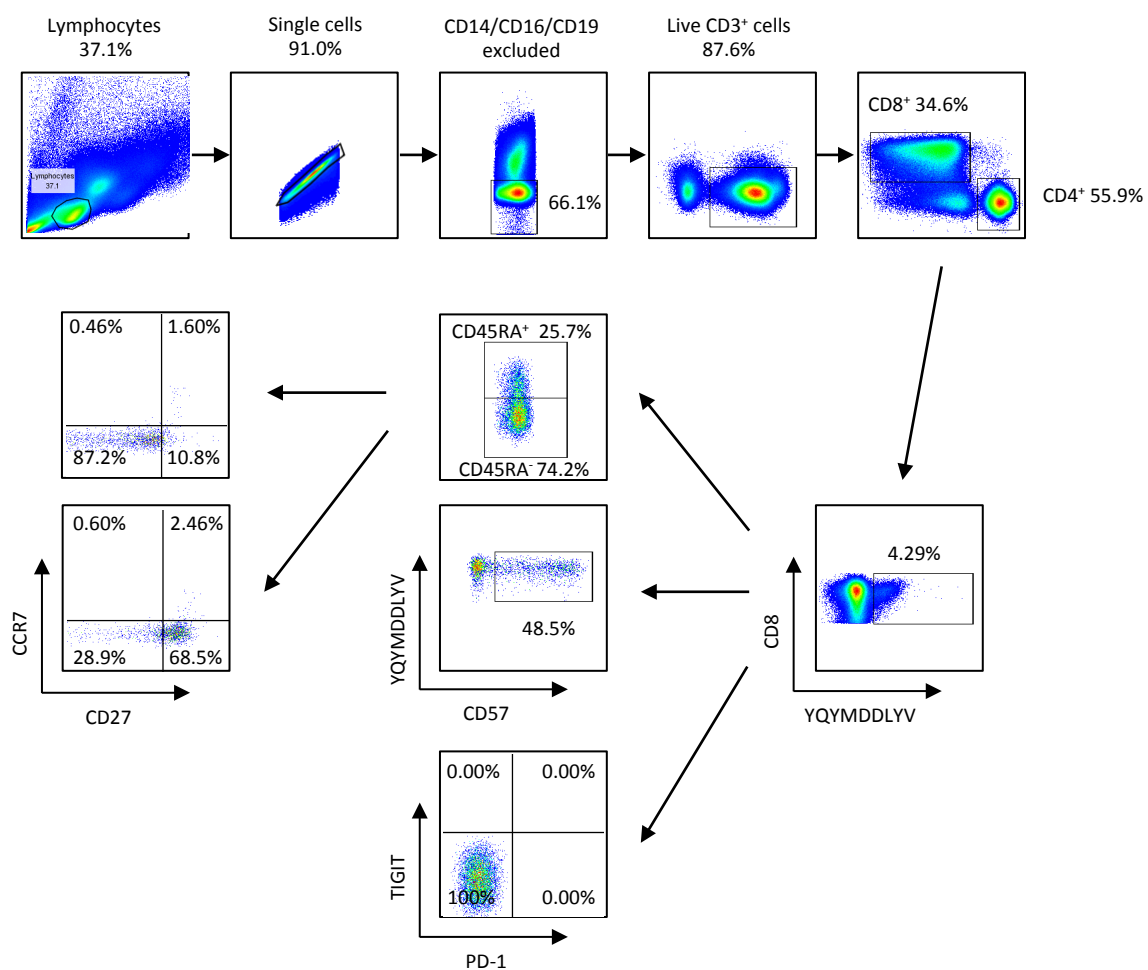

S1 Fig. Gating strategy for tetramer-reactive T cell analysis.

Supplement: S1 Fig — (PDF) [file pone.0197299.s004.pdf]
