# Supplementary material for: Randomized phase I trial HIV-CORE 003: Depletion of serum amyloid P component and immunogenicity of DNA vaccination against HIV-1
Source: PLoS One. 2018 May 17;13(5):e0197299. doi: 10.1371/journal.pone.0197299 (PMC5957335; doi:10.1371/journal.pone.0197299)
Supplement: S2 Text — (PDF) [file pone.0197299.s006.pdf]

**Full title of trial**	A randomised double-blind, placebo-controlled phase I/IIa trial to investigate the effect of depletion of serum amyloid P component (SAP) on the immune response to DNA vaccination in healthy male volunteers.
**Short title**	HIV-CORE003
**Version and date of protocol**	Version 2.2 7th May 2015
**Sponsor:**	University College London
**Sponsor protocol number**	11/0455
**Funder**	UK Medical Research Council
**EudraCT no**	2012-004052-11
**ACTIVE IMP(s):**	(R)-1-[6-[(R)-2-carboxy-pyrrolidin-1-yl]-6-oxo-hexanoyl]pyrrolidine-2-carboxylic acid (CPHPC)<br>pSG2.HIVconsv DNA vaccine<br>ChAdV63.HIVconsv vaccine<br>MVA.HIVconsv vaccine
**PLACEBO IMP(s):**	0.9% sodium chloride
**Phase of trial**	Phase I/IIa
**Sites(s)**	National Amyloidosis Centre and Private Patient Unit<br>Royal Free London NHS Foundation Trust<br>Pond Street, London, NW3 2QG

Chief investigator:

Dr Julian Gillmore
National Amyloidosis Centre,
Centre for Amyloidosis & Acute Phase Proteins,
UCL Division of Medicine, Royal Free Campus,
Rowland Hill Street,
London NW3 2PF.

Postal address:
As above.

Sponsor Representative:

Dr Nick McNally, Director of Research Support
Joint Research Office, 1st Floor Maple House,
149 Tottenham Court Road,
London W1T 7NF.

Postal address:
Joint Research Office, UCL
(1st Floor, Maple House),
Ground Floor, Rosenheim Wing,
25 Grafton Way,
London WC1E 6DB.

1 Signatures

The Chief Investigator and the JRO have discussed this protocol. The investigators agree to perform the investigations and to abide by this protocol

The investigator agrees to conduct the trial in compliance with the approved protocol, EU GCP and UK Regulations for CTIMPs (SI 2004/1031; as amended), the UK Data Protection Act (1998), the Trust Information Governance Policy (or other local equivalent), the GMO (Contained Use) Regulations 2000 (and subsequent amendments), the Research Governance Framework (2005' 2nd Edition; as amended), the Sponsor's SOPs, and other regulatory requirements as amended.

Chief investigator
Dr Julian Gillmore

Signature: [signature]   Date: 7th May 2015

Sponsor Representative

Dr Nick McNally
UCL

Signature: [signature]   Date: 4th August 2015

2 Contents

1 Signatures ... 3
2 Contents ... 4
3 List of abbreviations ... 9
4 Trial personnel ... 11
5 Summary ... 14
6 Introduction ... 17
6.1 Background ... 17
6.1.1 Vaccination to prevent or treat disease ... 17
6.1.2 Role of serum amyloid P component (SAP) ... 17
6.1.3 Addressing the HIV/AIDS pandemic ... 18
6.1.4 DNA vaccination after serum SAP depletion ... 18
6.2 Summary of pre-clinical data ... 18
6.2.1 CPHPC ... 18
6.2.2 pSG2.HIVconsv DNA, ChAdV63.HIVconsv and MVA.HIVconsv vaccine ... 19
6.3 Summary of clinical data ... 19
6.3.1 CPHPC ... 20
6.3.2 pSG2.HIVconsv DNA,ChAdV63.HIVconsv and MVA.HIVconsv vaccine ... 20
6.4 Rationale and risks/benefits ... 21
6.5 Assessment and management of risk ... 22
7 Objectives ... 22
7.1 Primary: ... 22
7.2 Secondary: ... 23
8 Trial design ... 23
8.1 Overall design and rationale for the chosen approaches ... 23
8.2 Study endpoints/outcome measures ... 24
8.2.1 Primary immunogenicity endpoint ... 24
8.2.2 Primary safety and tolerability endpoint ... 24
8.2.3 Secondary immunogenicity endpoints ... 24
9 Selection of subjects ... 25
9.1 Inclusion criteria ... 25

9.2 Exclusion criteria 25
10 Study procedures 27
10.1 Recruitment 27
10.2 Reimbursement 27
10.3 Study visits 27
10.3.1 Screening visit (visit 1) 27
10.3.2 Infusion/Vaccination visits 28
10.3.3 Non-infusion/vaccination (monitoring) visits during treatment period 29
10.3.4 Booster vaccination visit 30
10.3.5 Follow-up visits 30
10.3.6 End of study (EOS) or early termination (ET) visit 30
10.3.7 Unscheduled visits 31
10.3.8 Informed consent 31
10.3.9 Counselling regarding contraception & use of condoms 31
10.3.10 Medical history and physical examination 32
10.3.11 Blood and urine collection 32
10.3.12 Laboratory results 32
10.4 Blood sample processing 32
10.4.1 Routine biochemistry and haematology 33
10.4.2 Virology 33
10.4.3 HLA typing 33
10.4.4 Serum SAP levels 33
10.4.5 Plasma CPHPC levels 33
10.4.6 Cellular immunology assays 34
10.5 Assessments 34
10.5.1 Safety assessments 34
10.5.2 Local reactogenicity events 34
10.5.3 Systemic reactogenicity events 34
10.5.4 Other AEs 35
10.5.5 Routine laboratory parameters 35
10.5.6 Specific screening tests 35
10.5.7 Immunogenicity Assessments 35

10.5.8 Other assessments 35
10.6 Use of concomitant medications 36
10.7 Rescue medications 36
10.8 Randomisation and Blinding 36
10.9 Definition of End of Study 37
10.10 Unblinding 37
11 Investigational Medicinal Product 38
11.1 Name and description of investigational medicinal products 38
11.2 Packaging, storage and shipment 39
11.3 Description and justification of route of administration and dosage 40
11.4 Dosage, dosage modifications and method of administration 40
11.5 Dispensing and handling 40
11.6 Drug accountability 41
11.7 Assessment of compliance 41
11.8 Post-trial IMP arrangements 41
12 AEs, SAEs and SUSARs 41
12.1 Definitions 42
12.2 Severity grading of AEs 42
12.3 Assessment of causality and expectedness 43
12.4 Procedural expected AEs 44
12.5 Recording AEs 44
12.6 Reporting procedures 45
12.6.1 Notification of deaths 45
12.6.2 Development Safety Update Reports 45
12.6.3 Annual progress reports 45
12.6.4 Notification of serious breaches to GCP and/or the protocol 45
12.6.5 Reporting Urgent Safety Measures 46
13 Discontinuation/withdrawal of participants and stopping rules 46
13.1 Conditions under which a subject may be withdrawn from the trial 46
13.1.1 Follow-up after discontinuation 46
13.1.2 Replacement of subjects 47
13.2 Stopping rules 47

14 Management of HIV issues during and following the study ... 47
14.1 HIV testing ... 47
14.2 Social discrimination as a result of antibody response to IMP ... 47
15 Data handling ... 48
15.1 Data collection and record keeping at the trial site ... 48
15.2 Data entry onto the trial database ... 48
15.3 Data analysis ... 48
15.4 Confidentiality ... 48
15.5 Trial database ... 49
16 Quality control and quality assurance ... 49
17 Statistical considerations ... 49
17.1 Statistical analysis plan ... 49
17.2 Summary of baseline data ... 49
17.3 Primary outcome analysis ... 50
17.4 Secondary outcome analysis ... 50
17.5 Primary safety analysis ... 51
18 Sample size calculation ... 51
18.1 Randomisation methods ... 54
18.2 Planned recruitment rate ... 54
18.3 Interim analysis ... 54
18.4 Other statistical considerations ... 54
19 Names of committees involved in the trial ... 55
19.1 Trial Management Group ... 55
19.2 Independent Data Monitoring Committee ... 55
20 Direct access to source data/documents ... 55
21 Ethics and regulatory requirements ... 55
22 Monitoring requirement for the trial ... 56
23 Finance ... 56
24 Insurance ... 56
25 Archiving ... 56
26 Publication policy ... 57
27 Statement of compliance ... 57

28 References 58

3 List of abbreviations

ADL	Activities of daily living
AE	Adverse event
ALC	Absolute lymphocyte count
ALT	Alanine aminotransferase
ANC	Absolute neutrophil count
APR	Annual progress report
AR	Adverse reaction
AST	Aspartate aminotransferase
CI	Chief Investigator
CRF	Clinical Research Facility
CPHPC	(R)-1-[6-[(R)-2-carboxy-pyrrolidin-1-yl]-6-oxo-hexanoyl]pyrrolidine-2-carboxylic acid
CTIMP	Clinical Trial of Investigational Medicinal Product
DNA	Deoxyribonucleic acid
DSUR	Development Safety Update Report
EudraCT	European Clinical Trials Database
GCP	Good Clinical Practice
GMO	Genetically Modified Organisms
GMP	Good Manufacturing Practice
GP	General Practitioner
GSK	GlaxoSmithKline
HAART	Highly active antiretroviral treatment
IB	Investigator Brochure
ICF	Informed Consent Form
IDMC	Independent Data Monitoring Committee
IM	Intramuscular injection
IMP	Investigational Medicinal Product
IMPD	Investigational Medicinal Product Dossier
ISF	Investigator Site File
IV	Intravenous
LLN	Lower limit of normal

MHRA	Medicines and Healthcare products Regulatory Agency
NAC	National Amyloidosis Centre
NOAEL	No observable adverse event limit
NHS R&D	National Health Service Research & Development
PBMC	Peripheral blood mononuclear cells
PIS	Participant Information Sheet
PoC	Proof of concept
REC	Research Ethics Committee
RFH	Royal Free London Hospital
SAE	Serious Adverse Event
SAP	Serum amyloid P component
SAR	Serious Adverse Reaction
SOP	Standard Operating Procedure
SUSAR	Suspected Unexpected Serious Adverse Reaction
TMG	Trial Management Group
UCLH	University College London Hospital
UCL	University College London
ULN	Upper limit of normal

4 Trial personnel

Chief Investigator (CI)

Dr Julian Gillmore
National Amyloidosis Centre, Centre for Amyloidosis & Acute Phase Proteins,
UCL Medical School, Royal Free Campus,
Rowland Hill Street, London, NW3 2PF.
E-mail: j.gillmore@ucl.ac.uk
Tel: 020 7433 2726
Fax: 020 74332817

Sponsor's representative

Dr Nick McNally,

Director of Research Support
Joint Research Office, 1st Floor Maple House,
149 Tottenham Court Road,
London, W1T 7NF.

Postal address:
Joint Research Office, UCL,
Gower Street,

London, WC1E 6BT.
E-mail: n.mcnally@ucl.ac.uk
Tel: 020 7679 6639
Fax: 020 7679 0876

Statistician

Stephen Gerry
Medical Statistician,
Centre for Statistics in Medicine,
University of Oxford,
Botnar Research Centre,
Windmill Road, Oxford OX3 7LD
Email: Stephen.gerry@csm.ox.ac.uk
Tel: 01865 223457

Laboratories

1. For immunology assays and HLA typing:

Prof Tomas Hanke
The Jenner Institute
University of Oxford
Old Road Campus Research Building

Roosevelt Drive
Oxford OX3 7DQ
United Kingdom
E-mail: tomas.hanke@ndm.ox.ac.uk
Tel: +44 (0)1865 617630
Fax: +44 (0)1865 617608

2. For SAP assays:
Professor Sir Mark Pepys FRS
(Principal Investigator on the MRC DCS Award which is funding the study)
Wolfson Drug Discovery Unit
Centre for Amyloidosis & Acute Phase Proteins,
UCL Medical School, Royal Free Campus,
Rowland Hill Street, London, NW3 2PF.
E-mail: m,pepys@ucl.ac.uk
Tel: 020 7433 2801
Fax: 020 7433 2803

3. For CPHPC assays:
Gary Boyle
GlaxoSmithKline
E-mail: gary.w.boyle@gsk.com
Tel: 01920 882465

Co-investigators

1. Dr Lucy Dorrell
Target Discovery Institute,
NDM Research Building,
University of Oxford,
Old Road Campus, Off Roosevelt Drive,
Oxford, OX3 7FZ
E-mail: lucy.dorrell@ndm.ox.ac.uk
Tel: 01865 612904

2. Prof Tomas Hanke
The Jenner Institute
University of Oxford
Old Road Campus Research Building
Roosevelt Drive
Oxford OX3 7DQ
United Kingdom
E-mail: tomas.hanke@ndm.ox.ac.uk

Tel: +44 (0)1865 617630

Fax: +44 (0)1865 617608

3. Ms Thirusha Lane

National Amyloidosis Centre,

Address and fax as overleaf

E-Mail: t.lane@ucl.ac.uk

Tel: 020 7433 2759

Fax: 020 7433 2817

Trial Management Group

Dr Lucy Dorrell	lucy.dorrell@ndm.ox.ac.uk	01865 612904
Dr Julian Gillmore	j.gillmore@ucl.ac.uk	020 7433 2726
Prof Tomas Hanke	tomas.hanke@ndm.ox.ac.uk	01865 617630
Ms Thirusha Lane	t.lane@ucl.ac.uk	020 7433 2759

Role of Pentraxin Therapeutics Ltd

Pentraxin Therapeutics Ltd is the UCL spinout company founded by Sir Mark Pepys FRS to own all his IP, patents and proprietary information, in order to facilitate their development commercialisation and use in practice. The company owns all the granted patents covering CPHPC, some of which have been licensed exclusively to GSK.

Role of University of Oxford

Oxford University is the employer of Professor Tomáš Hanke, a co-applicant on the MRC award, and of his team, members of which under his supervision, will undertake the various tasks precisely detailed in the grant application, comprising arrangements for provision of the HIV vaccines, immunological assays of the responses by the participants to vaccination, and analysis and interpretation of the results of the study.

Role of GlaxoSmithKline

GlaxoSmithKline (GSK) has an exclusive License Agreement with Pentraxin Therapeutics Ltd for the development of CPHPC (also known as GSK2315698) for the treatment of systemic amyloidosis. This study is outside the scope of the License Agreement. GSK's role in this study will be limited to: supply of CPHPC and relevant preclinical, clinical and CMC data and measurement of CPHPC plasma concentrations. In accordance with the terms of the License Agreement and regulatory safety reporting responsibilities, safety data from this study will be shared with GSK.

5 Summary

**Title:**	A randomised double-blind, placebo-controlled phase I/IIa trial to investigate the effect of depletion of serum amyloid P component (SAP) on the immune response to DNA vaccination in healthy male volunteers.
**Short title:**	HIV-CORE003
**Trial medication:**	1. CPHPC<br>2. pSG2.HIVconsv DNA<br>3. ChAdV63.HIVconsv<br>4. MVA.HIVconsv<br>5. 0.9% sodium chloride (placebo)
**Phase of trial:**	I/IIa
**Objectives:**	***Primary:***<br>1. To demonstrate that the T cell immune response to three priming doses of DNA vaccine is significantly enhanced in SAP depleted subjects compared to identically vaccinated SAP sufficient control subjects.<br>2. To demonstrate that the T cell immune response to subsequent boosting by 2 separate recombinant virus vaccines expressing the same immunogen as the DNA vaccine is significantly enhanced in subjects who were primed by DNA vaccination whilst SAP depleted, compared to identically vaccinated SAP sufficient control subjects.<br>3. To confirm safety and tolerability of the treatment regimen involving three courses of intravenous (IV) infusion of CPHPC, to produce maximal depletion of plasma SAP, combined with intramuscular (IM) injection of DNA vaccine.<br>***Secondary:***<br>1. To characterise the *in vitro* inhibition of HIV-1 replication by HIVconsv-specific T cells induced by the above vaccination regimen.

Type of trial: Randomised double-blind placebo-controlled phase I/IIa trial in healthy male volunteers.

Grp	n	Week 0	Week 4	Week 8	Week 12	Week 16*
1	20	CPHPC<br>pSG2.HIVconsv DNA	CPHPC<br>pSG2.HIVconsv DNA	CPHPC<br>pSG2.HIVconsv DNA	ChAdV63.HIVconsv	MVA.HIVconsv
2	20	Placebo<br>pSG2.HIVconsv DNA	Placebo<br>pSG2.HIVconsv DNA	Placebo<br>pSG2.HIVconsv DNA	ChAdV63.HIVconsv	MVA.HIVconsv

Note: CPHPC = SAP depleter; pSG2.HIVconsv = DNA vaccination; ChAdV63.HIVconsv = booster vaccination; MVA.HIVconsv = second booster vaccination. (*33 subjects will receive the MVA.HIVconsv at week 16; subjects 1 to 7 will be invited back to receive the additional boost of MVA.HIVconsv as part of a separate follow-on study.)

Trial design and methods: 40 healthy male volunteers will be recruited via advertisements. All subjects will receive an identical DNA vaccination regimen, however, 20 will be randomised to SAP depletion prior to vaccination, and 20 will be SAP sufficient (randomised to receive placebo). The primary immunogenicity endpoint will be the magnitude and breadth of differences in T cell frequencies between randomised groups as measured by the *ex vivo* IFN-γ ELISPOT assay on PBMCs of subjects.

Trial duration per participant: Approximately 24 weeks

Estimated total trial duration: 30 months

Planned trial sites: 1

Total number of participants planned: 40

Main inclusion/exclusion criteria:

Inclusion:

1) Healthy males aged 18 to 50 years.
2) Willing to comply with the requirements of the protocol and available for follow-up for the planned duration of the study.
3) Willing to undergo HIV-1 testing, HIV-1 counselling and receive HIV-1 test results.

Exclusion:

1. Any relevant abnormality revealed from history or examination or from GP report.
2. Any clinically significant acute or chronic medical condition that is considered progressive or, in the opinion of the CI or designee, would make the volunteer unsuitable for the study.
3. Any clinically significant acute or chronic medical condition including abnormal laboratory findings.
4. Reported high-risk behaviour for HIV-1 infection.

5. Confirmed HIV-1 or HIV-2 infection.
6. Receipt of study-unrelated live attenuated vaccine within the previous 60 days or planned receipt within 60 days after vaccination
7. Receipt of blood transfusion or blood products within the previous six months.
8. Participation in another clinical trial of an IMP currently or within the previous three months.
9. Previous receipt of any investigational HIV-1 vaccine.
10. History of severe or very severe local or systemic reactogenicity events or allergic reactions.
11. Confirmed diagnosis of acute or chronic hepatitis B virus infection, confirmed diagnosis of hepatitis C virus infection; confirmed or suspected diagnosis of sexually transmitted infection.
12. Smallpox vaccination within the previous three years.
13. Major psychiatric illness.
14. Use of prescription or non-prescription drugs unless in the opinion of the CI the medication will not interfere with the study procedures or compromise subject safety.
15. Known allergy to study drugs' excipients.

**Statistical methodology and analysis:**	The primary analysis will involve the calculation of the difference between SAP depleted and control group participants in $\log_{10}$ transformed IFN-γ ELISPOT data at 16 weeks (12 weeks for first 7 volunteers, or after 3rd booster vaccine for those returning for participation in a separate follow-on study). This will be back transformed and presented as an n-fold difference between treatment groups with associated 95% confidence interval.

6 Introduction

6.1 Background

6.1.1 Vaccination to prevent or treat disease

Vaccination is one of the most important achievements of medicine. Injection of modified pathogenic organisms, or materials from them, induces protective immunity against the infections which they cause. Smallpox has been eradicated from the planet and polio is almost gone. Diphtheria, tetanus and pertussis have been essentially eliminated from developed countries and, had it not been for the mendacious campaign against MMR vaccine, measles could also have been greatly reduced. Successful immunisation induces a protective immune response against particular component(s) of the target pathogen, the so-called immunogen(s). For some diseases the immunogens are not known and for others they are difficult and expensive to produce, transport and administer, for example influenza vaccine must be produced in millions of chicken eggs.

A very attractive potential solution is parenteral injection of naked DNA encoding the pathogen-derived immunogen or epitopes. In this process, known as DNA vaccination, the DNA enters cells, predominantly at the site of injection, and causes them to produce the immunogen locally within the body. DNA vaccination works well and stimulates excellent protective immunity against a variety of different infections, and even some cancers, in mice, horses, dogs, rabbits and pigs. But in humans and other primates, and in cows and sheep, it has not been efficacious. Despite enormous academic and pharmaceutical industry efforts, the reasons for this failure have hitherto not been understood or overcome.

6.1.2 Role of serum amyloid P component (SAP)

Our group previously discovered that serum amyloid P component (SAP) is the single normal human plasma protein which binds strongly to DNA (1). We have lately discovered that there is complete concordance among species tested so far between the efficacy of DNA vaccination and the absence of SAP binding avidly to DNA. In particular, SAP in mice, which respond well to DNA vaccination, binds DNA very weakly (2). In contrast, nonhuman primates, cows and sheep share with humans the presence of SAP which strongly binds to DNA. We believe that binding of DNA by SAP may be responsible for blocking induction of immune responses by DNA vaccination and that depletion of SAP may overcome this inhibition.

SAP contributes to important human diseases, amyloidosis and Alzheimer's disease, and we have previously identified a drug, (R)-1-[6-[(R)-2-carboxy-pyrrolidin-1-yl]-6-oxo-hexanoyl]pyrrolidine-2-carboxylic acid (CPHPC), which removes almost all SAP from the blood in humans (3). It has lately been reported that human SAP potently blocks murine immune responses to DNA vaccination and that this inhibition is completely abrogated by CPHPC (4), (5). These observations confirm our hypothesis.

6.1.3 Addressing the HIV/AIDS pandemic

Development of an effective, accessible vaccine is the only realistic hope for halting the human immunodeficiency virus type 1 (HIV-1)/AIDS epidemic. Ideally, such a vaccine should induce broadly neutralizing antibodies and effective T cells at the same time. Both of these goals face substantial and very different challenges (6), with one major roadblock in common: the enormous HIV-1 genome plasticity, i.e. ability to change and escape immune responses. There is a need to develop vaccines which may be used both prophylactically and therapeutically to either prevent HIV-1 acquisition, control its replication without HAART and/or eventually eradicated the virus from the body completely.

The approach taken in the proposed clinical study aims to overcome the antigenic variation of HIV-1 by focusing induced T cell responses on the functionally conserved regions of HIV-1 proteins, which HIV-1 cannot change without a significant cost to its fitness. Thus, the HIVconsv immunogen is a chimaeric protein assembled from the 14 most conserved regions of the HIV-1 proteome alternating among the four most common HIV-1 clades: A, B, C and D (7). The gene coding for HIVconsv was made synthetically and was inserted into three safe non-replicating vaccine vectors: plasmid DNA to construct pSG2.HIVconsv, attenuated chimpanzee adenovirus to construct ChAdV63.HIVconsv and recombinant modified vaccinia virus Ankara to construct MVA.HIVconsv. These three vectors facilitate delivery of the immunogen gene into host cells, which then express the HIVconsv protein and initiate a series of processes leading to the presentation of HIVconsv-derived peptides to the cells of the host immune system and induction of the HIVconsv-specific host T cell responses.

6.1.4 DNA vaccination after serum SAP depletion

We now propose to undertake the first clinical proof-of-concept (PoC) study of DNA vaccination after SAP depletion. We will measure the immune responses to DNA vaccination against HIV-1 in healthy adult male volunteers, comparing a group in whom SAP has been completely depleted at the time of DNA vaccination and a control group vaccinated without SAP depletion. We predict that SAP depletion at the time of vaccination will enhance the immune response. A positive result, potentially consistent with improved protective immunity against HIV-1, will be very encouraging. Furthermore, proof of the concept that SAP depletion can enhance immune responses to DNA vaccination in humans will open the way in humans to use of prophylactic and therapeutic DNA vaccination against major infections including HIV-1, malaria and tuberculosis, and also cancer, with enormous potential health and economic benefits.

6.2 Summary of pre-clinical data

Please refer to the respective Investigator's Brochures (IB) for detailed information. Available preclinical data for each IMP is summarised hereunder.

6.2.1 CPHPC

In experimental models of murine reactive systemic (amyloid A protein [AA] type) amyloidosis, intraperitoneal or subcutaneous injections of CPHPC for 5 days resulted in inhibition of the uptake of

radiolabelled human SAP tracer and removal of endogenous mouse SAP from amyloid deposits at doses ≥1.5 mg/kg/day, in a dose-related manner. CPHPC administered in the drinking water was also effective in depleting circulating human SAP in the mouse amyloidosis model. CPHPC was well tolerated in IV single and repeat dose studies in rats and dogs at doses up to 400 and 200 mg/kg/day in the respective species. There were no findings in safety pharmacology or genetic toxicity studies that would preclude the intravenous or oral administration of CPHPC to humans.

6.2.2 pSG2.HIVconsv DNA, ChAdV63.HIVconsv and MVA.HIVconsv

No evidence of toxicity of the pSG2.HIVconsv DNA, ChAdV63.HIVconsv or MVA.HIVconsv vaccine candidates has been noted in non-GLP studies in mice and rhesus macaques to date (7), (8), (9),(10), (11). One GLP repeat dose toxicity study (study UNO 0012) has been conducted by Huntingdon Life Sciences with the vaccines in mice; in which the mice received 3 doses of the pSG2.HIVconsv DNA vaccine followed by a single dose of the ChAdV63.HIVconsv vaccine. Treatment with the vaccines was well-tolerated and not associated with any adverse systemic toxicological changes. All minor toxicity findings in the study were considered to be consistent with the predicted response to vaccine administration by intramuscular injection. The results of the UNO0012 study were reviewed and summarised in the IB and the full report is available on request.

Two separate pre-clinical GLP toxicology studies have been carried out by Huntingdon Life Sciences. Study UNO0011 assessed the systemic toxic potential of 2×10^7 pfu/dose of MVA.HIVconsv administered by intramuscular injection to groups of 10 male and 10 female BALB/c mice on days 1, 15 and 29 (the MMM regimen). Vaccinations were not associated with any systemic toxicological changes. The findings of increased cellularity of the draining lymph nodes, high plasma gamma globulin, concentration and aspartate aminotransferase activity and inflammatory changes at the dose sites were considered to be consistent with a predicted response to vaccine administration. These results supported the Medicines and Healthcare products Regulatory Agency (MHRA), UK approval for clinical trial HIV-CORE 001.

Study UNO0012 assessed in similar groups of BALB/c mice the systemic toxic potential following administration of 50 µg/dose of pSG2.HIVconsv DNA administered by intramuscular injection on days 1, 15 and 29, followed by an intramuscular injection of 5×10^9 vp ChAdV63.HIVconsv on day 43 (the DDDC regimen). Again, vaccinations were not associated with any systemic toxicological changes and all the observed lymph node changes were consistent with response to the vaccine administration. The UNO0011 toxicology protocol MMM together with the UNO0012 protocol DDDC supported the MHRA UK approval of the Clinical Protocol HIV-CORE 002 (15).

6.3 Summary of clinical data

Please refer to the respective IBs for detailed information. Available clinical data for each IMP is summarised hereunder

6.3.1 CPHPC

In previous academic sponsored clinical investigations published in peer-reviewed journals, CPHPC was generally well-tolerated and, other than discontinuation for local injection site discomfort in a few patients, no significant drug-related adverse safety findings were identified (12). A GSK sponsored clinical study in healthy volunteers was performed to provide safety information and to explore the PK/PD relationship for depletion of circulating SAP by IV administration of CPHPC. Infusion regimens ranged from 5 mg/hr for 1 hour to 40 mg/hr for 24 hours, with a total dose range of 5 to 960 mg. All of the administered regimens were well tolerated. No dose limiting adverse events or safety findings were observed over an exposure range that included approximate parity with the NOAEL (No Observed Adverse Effect Level). A GSK-sponsored PK/PD study involving intravenous doses of CPHPC ranging from 5 to 30 mg/hr for 48 hour infusions (total dose range 240 to 1440 mg) into patients with systemic amyloidosis is ongoing, and the regimen has been generally well tolerated.

6.3.2 pSG2.HIVconsv DNA, ChAdV63.HIVconsv and MVA.HIVconsv

Vaccine candidates ChAdV63.HIVconsv (C), MVA.HIVconsv (M) and pSG2.HIVconsv DNA (D) have been evaluated in a single-blind placebo-controlled phase I trial in healthy volunteers, HIV-CORE002 (EudraCT No. 2010-018439-16), sponsored by University of Oxford. Two volunteers received one vaccination with ChAdV63.HIVconsv $1.2x10^8$ infectious units (IU) (for this batch equivalent to $5x10^9$ virus particles) for initial safety evaluation of the ChAdV-63 vector. Thirty volunteers were then recruited and assigned sequentially in groups of 10 to one of three vaccination regimens: CM (weeks 0 and 8), DDDCM (weeks 0, 4, 8, 12 and 20) or DDDMC (weeks 0, 4, 8, 12 and 16). Within each group, subjects were randomised to receive vaccine or placebo in a 4:1 ratio. Vaccine doses were: ChAdV63.HIVconsv $1.2x10^9$ IU, MVA.HIVconsv $2x10^8$ pfu, pSG2.HIVconsv DNA 4 mg. All vaccines were given via the IM route and each dose divided between two arms. 28/30 subjects completed the vaccination schedule; two subjects withdrew after receiving one vaccination, for reasons unrelated to safety or tolerability.

Overall, safety and tolerability profiles were excellent. There were no SUSARs and the majority of local and systemic vaccination reactions were grade one and lasted <48 hours (erythema/pain at injection site, myalgia, 'flu'-like symptoms, headache. One volunteer experienced fever >38.5°C. There was one serious adverse event that was unrelated to any of the investigational vaccines (appendicitis occurring after enrolment and before receipt of any vaccine (16).

Clinical trial HIV-CORE 001 (Eudract: 2009-012662-31) was a phase I trial sponsored by University of Oxford, which ended in November 2013 evaluating MVA.HIVconsv in HIV-1 chronically infected adults receiving HAART. Patients received vaccine regimens of mmm or MMM (8 vaccinees + 2 placebos per arm) at $5x10^7$ pfu and $2x10^8$ pfu per dose, respectively, at 0, 4 and 12 weeks.

All vaccine administrations of MVA.HIVconsv were been well tolerated in the HIV-1-positive subjects. The reactogenicity profile is similar to that of healthy HIV-1-uninfected volunteers. There were no SUSARs. There were 3 serious adverse events (pneumonia, as described above, Hodgkin's lymphoma, pyelonephritis) – all were unrelated/unlikely related to the investigational vaccine.

Preliminary immunogenicity data analysis of HIV-CORE 001 indicates that MVA.HIVconsv vaccinations increased the breadth of responses to conserved HIV epitopes and increased CD8+ T cell viral inhibitory capacity in 5 of the volunteers, particularly those receiving the higher MVA.HIVconsv dose. Formal data analysis by a statistician is ongoing.

Clinical trial BCN 01 (EudraCT number 2011-000846-39) is sponsored by IrsiCaixa AIDS Research Institute, and assesses ChAdV63.HIVconsv and MVA.HIVconsv in heterologous vaccination regimens CM and C_M (volunteers 12 per arm) as a therapeutic strategy in a population of HIV-1-infected individuals with early viral suppression by Tenofovir/Emtricitabine plus Raltegravir and a 6-month treatment. BCN 01 is a phase I, multicentre study. The C and M vaccines are delivered intramuscularly according to a 0-8 weeks or a 0-24 weeks schedules. All vaccinations have been completed. To date, the vaccines were well tolerated. There have been no SAEs or SUSARs. There have been no confirmed increases in viral load or significant changes in CD4 counts.

6.4 Rationale and risks/benefits

Prophylactic immunisation against, for example, HIV-1, hepatitis C, tuberculosis, malaria and other infectious diseases, and the lack of effective non-surgical treatments for most forms of cancer, are massive unmet medical needs. Vaccination with DNA encoding the immunogens responsible for protective immunity to infection and cancer is a potentially very powerful approach to prophylaxis and treatment of many major, hitherto poorly treatable diseases. DNA vaccination works efficiently in some animals and high immunogenicity has led to the development and marketing of three veterinary DNA vaccines. Unfortunately, humans and other primates do not mount clinically useful immune responses to DNA vaccines. After considerable efforts to understand and overcome this failure, most large pharmaceutical companies have abandoned work on DNA vaccination in humans. If the approach proposed here is successful the benefits would be applicable to patients and healthy individuals who are at risk worldwide.

GSK has recently undertaken a comprehensive formal phase I study of IV infusion of CPHPC. Data from this study show that depletion of circulating SAP to very low levels can be achieved within 24 hours in healthy volunteers using an intravenous infusion regimen. All of the administered dosing regimens were well tolerated; please see the IB for details. The pSG2.HIVconsv DNA vaccine and the ChAdV63.HIVconsv and MVA.HIVconsv vaccines have been evaluated in a clinical trial in healthy HIV-1 uninfected adult volunteers in trial HIV-CORE002 completed in August 2012, and showed no dose-limiting adverse effects or safety findings. Whilst the combined administration of these four IMPs is novel, information on the safety profile of each individual IMP is available, and we do not anticipate any untoward effects of the combined treatment. Seven subjects have completed this trial to date and there have been no SAES , SUSARS or unexpected toxicities. However in the interests of safety, the treatment phase of the study will be carried out at the Private Patients Unit and the National Amyloidosis Centre at the Royal Free Hospital.

6.5 Assessment and management of risk

This trial is categorised as 'Type C' i.e. the trial carries risk which is markedly higher than that of standard medical care. This is based on the fact that the study subjects are healthy volunteers, and there is therefore no standard medical care. Whilst the study team, together with the wider collaborative team, have extensive clinical experience with the individual IMPs, the administration of the vaccines after SAP depletion is a novel approach. The treatment phase of the study will therefore be conducted within the private patient unit with full overnight monitoring and access to emergency facilities and an outpatient's clinic. Dosing of the first two subjects, one of whom was randomised to active treatment (CPHPC) and the other to placebo, took place concurrently, with intensive monitoring at a clinical research facility, and further subjects were dosed only once the first two subjects had safely completed the first dosing visit, and the safety data from the week 0 and 2 visits had been reviewed and approved by the CI and the independent data monitoring committee (IDMC). For subsequent dosing cohorts (of up to 6 subjects) there will be a minimum interval of three weeks between the first dose for each cohort to allow for review by the PI of safety data from the week 0 and 2 visits. Within the study dosing regimen the CPHPC is administered before the vaccine, so if the infusion is not well tolerated it may be stopped before the vaccine is given. Safety data of each subsequent subject will be reviewed by the PI/designee after each infusion/vaccination visit, and further infusions/vaccinations will not occur until this has been done. Additional safety oversight will be provided by the IDMC who will review data at key points of the study and if emergent issues arise. Risks associated with the use of the pSG2.HIVconsv DNA, ChAdV63.HIVconsv and MVA.HIVconsv vaccines as GMO are negligible as none of the three vaccines can replicate and give rise to propagating progeny following injection.

The transgene product is an un-natural protein of no documented biological activity other than being able to induce T cell responses to its processed parts/peptides.

7 Objectives

7.1 Primary:

1. To demonstrate that the T cell immune response to three priming doses of DNA vaccine is significantly enhanced in SAP depleted subjects compared to identically vaccinated SAP sufficient control subjects.

2. To demonstrate that the T cell immune response to subsequent boosting by 2 separate recombinant virus vaccines (ChAdv63.HIVconsv only in any of the 1st 7 subjects who do not consent to receiving the MVA.HIVconsv boost) expressing the same immunogen as the DNA vaccine is significantly enhanced in subjects who were primed by DNA vaccination whilst SAP depleted, compared to identically vaccinated SAP sufficient control subjects.

3. To confirm safety and tolerability of the treatment regimen involving three courses of IV infusion of CPHPC, to produce maximal depletion of plasma SAP, combined with IM injection of DNA vaccine.

7.2 Secondary:

To characterise the *in vitro* inhibition of HIV-1 replication by HIVconsv-specific T cells induced by the above vaccination regimen(s).

8 Trial design

8.1 Overall design and rationale for the chosen approaches

The purpose of this double-blind, placebo-controlled study is to test whether SAP depletion enhances the immune response to DNA vaccination in general, rather than specifically to create an efficacious vaccine at this stage against any particular pathogen. SAP depletion will be achieved by an IV infusion of 40 mg of CPHPC per hour for 26 hours. This regimen is based on the GSK healthy volunteer phase I study in which plasma SAP concentration was reduced to <0.2 mg/L in all cases. We expect that the SAP in the extracellular fluid compartment will be similarly depleted at 24 hours, since plasma values are already well below 1 mg/l, that is ~97% reduced from normal, within four to six hours. The vaccination will be given after 24 hours of infusion, and the CPHPC/placebo infusion will continue for a further two hours after the vaccination. CPHPC is highly water soluble with a maximal extracellular volume of distribution (unpublished) so that it will be abundantly present at the site of DNA injection and thus able to block residual SAP binding to DNA.

DNA vaccination will be administered by IM injection after 24 hours of CPHPC infusion. Although the extracellular persistence of such DNA is known to be extremely brief (13) we will continue the CPHPC infusion for a further two hours after vaccination, and the proposed timing will therefore be appropriate to rigorously test the effect of SAP depletion at the time of DNA vaccination. The control group will receive an IV saline infusion identical to the test group but without any CPHPC. The choice of HIVconsv as the DNA immunogen has been facilitated because of the ready availability in a pharmaceutical form manufactured to EU GMP standard (manufactured according to the rules governing medicinal products in the European Union, Volume 4, including Annex 13) in Oxford, and the team's extensive current experience with this system, as well as the potential clinical rewards of a successful outcome. The HIVconsv vaccines focus on induction of T cells specific for HIV-1 and are currently being tested in the HIV-CORE002 (so-called for **CO**nserved **RE**gions) phase I trial in healthy volunteers in Oxford without HIV-1/2 infection. The immunisation regimens being examined are CM, DDDCM and DDDMC, where 'D', 'C' and 'M' stand, respectively, for plasmid pSG2.HIVconsv DNA, attenuated chimpanzee adenovirus ChAdV63.HIVconsv and attenuated poxvirus MVA.HIVconsv. HIV-CORE002 recruitment started in March 2011 and the last volunteer/last blood sample in the first phase of this trial took place in August 2012. An application to extend the study to test volunteer's vaccine-elicited responses at 6, 12 and 24 months after the last vaccination has been granted. The HIV-CORE003 trial proposed here will test a regimen of priming with plasmid DNA followed by boosting with recombinant attenuated chimpanzee adenovirus and recombinant modified vaccinia virus Ankara, and will compare the responses between groups with and without depletion of SAP before each plasmid DNA dose. The effect of SAP depletion on the response to immunisation with DNA alone will be determined and the influence of this T cell priming on the response to the

subsequent recombinant adenovirus boost will also be evaluated. Substantial differences between the groups will establish PoC that SAP depletion can enhance the immune response to DNA vaccination.

Forty subjects will be enrolled; the first 7 have received the DDDC immunisation regimen of three administrations of pSG2.HIVconsv DNA (D) followed by one administration of ChAdV63.HIVconsv (C). By amendment to the protocol, a further 33 will receive the DDDCM immunisation regimen (M = MVA.HIVconsv); subjects 1 to 7 will be invited to receive the MVA.HIVconsv booster and identical follow-up thereafter as part of a separate follow-on study. The volunteers will be randomized into two groups of 20 for depletion (i.e. receipt of CPHPC) (Group 1) or no depletion (i.e. receipt of saline placebo) (Group 2) of SAP (Table 1).

Group	n	Week 0	Week 4	Week 8	Week 12	Week 16*
1	20	CPHPC<br>pSG2.HIVconsv DNA	CPHPC<br>pSG2.HIVconsv DNA	CPHPC<br>pSG2.HIVconsv DNA	ChAdV63.HIVconsv	MVA.HIVconsv
2	20	Placebo<br>pSG2.HIVconsv DNA	Placebo<br>pSG2.HIVconsv DNA	Placebo<br>pSG2.HIVconsv DNA	ChAdV63.HIVconsv	MVA.HIVconsv

Table 1. Vaccination regimens. CPHPC = SAP depleter; pSG2.HIVconsv = DNA vaccination; ChAdV63.HIVconsv = booster vaccination; MVA.HIVconsv = second booster vaccination. (*33 subjects will receive the MVA.HIVconsv at week 16; the 1st 7 subjects will be invited back to receive the additional boost of MVA.HIVconsv as part of a separate follow-on study).

8.2 Study endpoints/outcome measures

8.2.1 Primary immunogenicity endpoint

The primary immunogenicity endpoint will be the magnitude and breadth of differences in T cell frequencies between randomised groups as measured by the *ex vivo* IFN-γ ELISPOT assay on peripheral blood mononuclear cells (PBMCs) of subjects.

8.2.2 Primary safety and tolerability endpoint

The primary safety and tolerability endpoint will be the development of grade 3 or 4 local or systemic reactions, as defined in appendix D, after administration of CPHPC infusion followed by either of the HIVconsv vaccines.

8.2.3 Secondary immunogenicity endpoints

Further characterization of the vaccine elicited immune responses. Several immunological research-grade, state-of-the-art assays will be employed, which may include Intracellular Cytokine Staining to determine contribution of the CD4 and CD8 T cell subsets, their functionality in terms of production of intercellular signalling molecules, proliferation to recall HIV antigens and *in vitro* inhibition of HIV-1 replication in autologous cells.

9 Selection of subjects

Testing immunogenicity of vaccines in patients with compromised immune responses may not properly reflect the safety and immunogenicity of vaccines in healthy volunteers. The good safety profiles in humans of the vaccines also strongly support evaluation of our vaccine regimens first in HIV-1-negative volunteers rather than in HIV-1-infected patients.

All 40 volunteers in this trial will be deemed to be at low risk of HIV-1 infection. Subjects will be adult males aged 18-50 who fully comprehend the purpose and details of this study as provided in the PIS and are able to provide written informed consent. Eligibility will depend on the results of laboratory tests, review of medical histories, physical exam results and answers to questions about risk behaviours.

9.1 Inclusion criteria

1) Healthy males, as assessed by a medical history, physical examination and laboratory tests.
2) Aged at least 18 years on the day of screening and no greater than 50 years on the day of the first vaccination.
3) Willing to comply with the requirements of the protocol and available for follow-up for the planned duration of the study.
4) In the opinion of the CI or designee, the volunteer has understood the information provided and is able to provide written informed consent, which includes compliance with the requirements and restrictions listed in the consent form.
5) Willing to undergo HIV-1 testing, HIV-1 counselling and receive HIV-1 test results.
6) If heterosexually active male; willing to use an effective method of contraception from the day of the first vaccination until six weeks after the last vaccination.
7) Willing to forgo donating blood during the study.

9.2 Exclusion criteria

1) Any relevant abnormality revealed from history or examination or from GP report, including history of immunodeficiency or autoimmune disease, or use of systemic corticosteroids, immunosuppressive, antiviral, anticancer or other medication that, in the opinion of the CI or designee, is clinically significant, within the previous six months.
2) Any clinically significant acute or chronic medical condition that is considered progressive or, in the opinion of the CI or designee, would make the volunteer unsuitable for the study.
3) Any of the following abnormal laboratory parameters listed below:

   <u>Haematology</u>

   - Haemoglobin < 10.0 g/dl
   - Absolute Neutrophil Count (ANC) ≤ 1000 /mm^3 (≤ 1 x 10^9 /l)
   - Absolute Lymphocyte Count (ALC) ≤ 600 /mm^3 (≤ 1 x 10^9 /l)
   - Platelets ≤100,000 /mm^3, ≥ 550,000 /mm^3 (≤ 90 /l, ≥ 550 /l)

   <u>Biochemistry</u>

   - Creatinine > 1.3 x upper limit of normal (ULN)

- Aspartate aminotransferase (AST) > 2.5 x ULN
- Alanine aminotransferase (ALT) > 2.5 x ULN

Urinalysis

- Abnormal dipstick (haematuria or significant proteinuria) confirmed by microscopy or biochemistry

4) Volunteer self-reported high-risk behaviour for HIV-1 infection. High-risk behaviour for HIV-1 infection is defined as follows. Within the previous six months the volunteer has:
   - Had unprotected vaginal or anal sex with a known HIV-1-infected person or a casual partner (i.e. not continuing, established relationship)
   - Engaged in sex work for money or drugs
   - Used injection drugs
   - Acquired a sexually transmitted infection
5) Confirmed HIV-1 or HIV-2 infection.
6) Receipt of live attenuated vaccine within the previous 60 days before first dose or planned receipt during treatment phase, or within 60 days after last vaccination with IMP, or receipt of other vaccine, including influenza vaccine, within the previous 14 days before first dose, during treatment phase, or planned receipt within 14 days after vaccination with the IMP.
7) Receipt of blood transfusion or blood products within the previous six months.
8) Participation in another clinical trial of an IMP currently or within the previous three months or expected participation during this study.
9) Previous receipt of any investigational HIV-1 vaccine. History of severe or very severe local or systemic reactogenicity events or history of severe or very severe allergic reactions.
10) Confirmed diagnosis of acute or chronic hepatitis B virus infection (spontaneous clearance leading to natural immunity, indicated by antibodies to core + antigens, is not an exclusion criterion); confirmed diagnosis of hepatitis C virus infection; confirmed or suspected sexually transmitted infection.
11) Smallpox vaccination within the previous three years (smallpox vaccination prior to three years should be documented but is not an exclusion criterion).
12) Major psychiatric illness including any history of schizophrenia or severe psychosis, bipolar disorder requiring therapy, suicidal attempt or ideation in the previous three years.
13) Use of prescription or non-prescription drugs, including vitamins, herbal and dietary supplements (including St John's Wort) within seven days (or 14 days if the drug is a potential enzyme inducer) or five half-lives (whichever is longer) prior to the first dose of study medication, unless in the opinion of the CI the medication will not interfere with the study procedures or compromise subject safety.
14) Known allergy to excipients of study drugs.

10 Study procedures

10.1 Recruitment

Potential participants will be recruited through advertisements in for example local newspapers, hospitals and university colleges. Interested persons will be offered an informal discussion with the CI, co-investigator, or study physician, when they will be provided with information on the vaccine and the study protocol and will be given a copy of the PIS. They will have the opportunity to ask questions and to arrange an appointment for a screening visit if they wish to take part. Volunteers recruited in the study will be free to leave the study at any time.

10.2 Reimbursement

Volunteers will be reimbursed for their time, effort and travel costs to the study site due to study participation. Reimbursement amounts will be documented in the PIS.

10.3 Study visits

All visits will take place at the Royal Free Hospital (RFH) which is part of the Royal Free London NHS Foundation Trust. Visits 2b, 4b and 6b (the infusion visits) will begin at the Private Patient Unit (PPU) which is an inpatient ward within the RFH. Volunteers will be admitted to the PPU for commencement of the CPHPC/placebo infusion and will remain inpatients there for overnight observation. Medical cover will be provided by the on-call medical team. Volunteers will be discharged from the PPU the next morning to the National Amyloidosis Centre (NAC), where the rest of the visit will take place. All other study visits will take place at the NAC. All visits and procedures are laid out in the Schedule of Procedures (Appendix A).

The screening visit can take place up to 28 days prior to visit 2; visits 3, 5, 7, 9, 10, 12 and 13 will have a +/- 3 day window; the pre-check visits (2a, 4a and 6a) will have a +/- 1 day window; however, the infusion/vaccination visits (2b, 4b and 6b) will have no allowable visit window.

10.3.1 Screening visit (visit 1)

Each subject who consents to screening will be assigned a unique screening number, and these screening numbers will be sequential. At screening (Visit 1, up to 28 days prior to day 0 and at least 24 hours after giving the volunteer the PIS), site personnel (trial nurse/physician/co-investigator) will perform the following:

- Review the PIS and Informed Consent Form (ICF) prior to obtaining written informed consent
- Obtain written informed consent before performing any study procedures.

If the volunteer agrees to participate, site personnel will:

- Give the volunteer the opportunity to ask any additional questions about study procedures
- Confirm study eligibility

- Obtain a complete medical history including concomitant medication (prescribed and non-prescribed)
- Perform a directed physical examination including weight, electrocardiogram (ECG) and vital signs (oral temperature, resting heart rate [HR] and blood pressure [BP]) will be measured after the participant has rested for at least five minutes
- Obtain blood samples for laboratory investigations indicated in Appendix A.
- Contact the volunteer's General Practitioner (GP) to obtain a medical history.

Screening laboratory test (s) may be repeated once at the discretion of the CI or designee to investigate any isolated abnormalities. If the screening visit occurs more than 28 days prior to the date of vaccination all screening procedures must be repeated. In such cases the complete medical history may be replaced by an interim medical history and the ICF should be reviewed if necessary. Subjects will be randomised to a treatment arm once all screening results and the GP report have been approved by the CI or delegate.

10.3.2 Infusion/vaccination pre-check visits (visits 2a, 4a and 6a)

The purpose of this visit is to confirm fitness and eligibility to proceed with the infusion/vaccination visit. At these visits study staff will:

- Review any adverse events (AEs) and concomitant medications
- Confirm/re-confirm eligibility, including review laboratory data from the screening/previous visit
- Perform a directed physical examination including vital signs in addition to any further examination indicated by history or observation
- Collect blood specimens for tests as specified in Appendix A.

10.3.3 Infusion/Vaccination visits (visits 2b, 4b and 6b)

All infusion/vaccination visits will begin at the PPU. At the first infusion/vaccination visit (visit 2b) study staff will check that screening and randomisation have been completed. At visits 2b (week 0), 4b (week 4), and 6b (week 8) volunteers will receive CPHPC (n=20) or placebo (n=20) infusion over 26 hours. On visits 2b, 4b and 6b all volunteers (n=40) will receive IM DNA vaccination 24 (+ ½) hours after commencement of the CPHPC infusion. These visits are specified in the Schedule of Procedures (Appendix A) and infusion/vaccination visit procedures are specified in Details of Inpatient Stay (Appendix B).

For the first half-hour of the infusion, subjects will remain under continuous observation by site staff to avoid undetected anaphylactic reactions. During the infusion, site staff will monitor vital signs half-hourly for the first two hours, two-hourly for the next four hours and then at 12 and 24 hours (see Appendix B for further detail). Site staff will also monitor volunteers for local and systemic reactogenicity after both the infusion and the vaccination at time-points listed in Appendix B, and these will be recorded as AEs if they occur.

The vaccination will be given 24 (+ ½) hours after the CPHPC/placebo infusion has commenced (i.e. the vaccine may be given between 24 hours and 24 ½ hours after the start of the infusion). The vaccination should be given after blood sampling and after the last vital sign measurement at 24 hours (± 10 min) post-infusion, and these measurements will serve as the pre-vaccination vital signs. The CPHPC/placebo infusion will continue for approximately 2 hours after the vaccination (approximately 26 hours from commencement).

After the vaccination, site personnel will:

- Cover the vaccination sites with semi-occlusive dressings
- Observe the subject closely for 30 minutes for signs of acute reaction
- Monitor vital signs after 30 minutes, 2 hours and 4 hours post-vaccination
- Record any local reaction at the site of vaccine administration as an AE (Appendix C).

Once the infusion has been completed, subjects will remain at the NAC for 2 hours for observation, and vital signs will be recorded as per the post-vaccination schedule detailed below and in Appendix B. If the site staff are satisfied that the vital signs and reactogenicity assessments after the 4-hour post-vaccination time-point are within the normal range, the subject may be discharged without medical review.

Prior to discharge subjects will be provided with thermometers and diary cards, and will be instructed on how to measure and record their body temperature for the next 3 days.
Site staff will contact subjects by telephone after three to five days to review AEs and concomitant medications. If necessary subjects may be brought in for an unscheduled visit (see 10.3.7).

10.3.4 Non-infusion/vaccination (monitoring) visits (visits 3, 5, 7, 9, 10, 12, and 13)

All visits will take place at the NAC. At visit 3 (week 2), visit 5 (week 6), visit 7 (week 10), visit 9 (week 13), visit 12 (week 17), and visit 13 (week 18), study staff will perform the following :

- Review any adverse events (AEs) and concomitant medications
- Where indicated perform a directed physical examination. NOTE: Medical review at these visits is only required where there are new AEs revealed on questioning or examination of vital signs by the study nurse, or if there are ongoing AEs **which require medical review**)
- Measure and record vital signs
- Collect and review diary card and retain with the case report forms (or review electronic record of diary card and transcribe into source document, where applicable) (visits 3, 5, 7, 9 and 12 only)
- Review safety laboratory data from the previous visit
- Collect blood specimens for tests as specified in the schedule of procedures

10.3.5 Booster vaccination visits (visits 8 and 11)

On visit 8 (week 12) volunteers will attend the NAC to receive IM ChAdv63.HIVconsv booster vaccination only (without prior CPHPC/placebo infusion). On visit 11 (week16) volunteers will attend the NAC to receive IM MVA.HIVconsv booster vaccination only (without prior CPHPC/placebo infusion). Prior to each booster vaccine administration site staff will:

- Review any adverse events (AEs) and concomitant medications
- Where indicated perform a directed physical examination
- Measure and record vital signs
- Review safety laboratory data from the previous visit (if not previously reviewed)
- NOTE: Fitness to proceed to with booster vaccination will be documented in medical notes/source documents by PI/sub-investigator/study physician after review of labs and AEs after visits 7 and 10. Thus there will be no requirement for medical assessment or physical examination prior to booster vaccinations at visits 8 and 11 unless indicated by emergence of new AE.
- Collect blood specimens for tests as specified in the schedule of procedures

Thereafter, the booster vaccinations will be administered. Subjects will remain at the NAC for observation for two hours post-vaccination. Vital signs will be recorded at ½ hour and 2 hours after the booster vaccinations. If site staff are satisfied that the 2 hour vital signs and reactogenicity assessments are with the normal range, the subject may be discharged without medical review. In the event that abnormalities are detected vital signs will be measured half-hourly until results are within the normal range or deemed clinically insignificant by the PI/study physician.

Prior to discharge subjects will be provided with thermometers and diary cards, and will be instructed on how to measure and record their body temperature for the next 3 days.

Site staff will contact subjects by telephone after three to five days to review AEs and concomitant medications. If necessary subjects may be brought in for an unscheduled visit.

10.3.6 End of study (EOS) or early termination (ET) visit

The EOS visit (visit 14, week 20) or ET visit procedures (see section 13) will be performed according to the schedule of procedures. Site personnel will:

- Review any adverse events (AEs) and concomitant medications
- Perform a directed physical examination
- Measure and record vital signs
- Collect and review diary card and retain with the source documents (where indicated)
- Review safety laboratory data from the previous visit (if not previously reviewed)
- Collect blood specimens for tests as specified in the schedule of procedures
- In the case of early withdrawal, ascertain reasons therefore, where possible.

10.3.7 Unscheduled visits

Visits/contacts other than those described in the schedule of procedures, for example in response to a telephone follow up, will be classified as unscheduled visits and recorded on a designated case report form page. They may occur:

- For administrative reasons
- To review a laboratory investigation from a previous visit
- To repeat an examination or blood test
- To review the outcome of an AE
- To conduct a study visit where a volunteer has missed the scheduled study visit window (these would normally be carried out in keeping with the study protocol and noted as a deviation)
- For any other reason requested by the volunteer or CI/delegate.

10.3.8 Informed consent

Potential participants will be given the PIS (in person, or by post or e-mail), and its content will be reviewed with them in person or over the telephone to enable discussion of: the exact nature of the study; the implications and constraints of the protocol; and the known side effects and any risks involved in taking part. It will be clearly stated that the participant is free to withdraw from the study at any time for any reason without prejudice to future care, and with no obligation to give the reason for withdrawal. Participants will be allowed as much time as they require (a minimum of 24 hours) to consider the information, and to question the CI/designee, their GP or other independent parties to decide whether they will participate in the study. Written informed consent will then be obtained in person by means of participant dated signature and dated signature of the person who presented and obtained the informed consent. The person obtaining the consent will be suitably qualified and experienced and will have been authorised to do so by the CI. Consent will not denote enrolment into trial. No study procedures will be undertaken prior to obtaining written informed consent. A copy of the signed PIS and ICF will be given to the participants and the original signed form will be retained at the study site in a locked cabinet. A copy will be sent to the UCLH site where the treatment phase of the trial will be undertaken, and a copy will be sent to their GP. If new safety information results in significant changes in the risk/benefit assessment, the consent form will be reviewed and updated if necessary and subjects will be re-consented as appropriate.

10.3.9 Counselling regarding contraception & use of condoms

Site personnel will counsel study participants about the importance of preventing pregnancies and about effective methods of contraception including correct use of condoms. Participants will be advised on where they may obtain condoms if required.

Subjects with female partners of child-bearing potential must use one of the following contraceptive methods after the first dose of study treatment until the EOS visit:

- Condom with spermicide <u>plus</u> partner use of a highly effective contraceptive (see list below).

- Abstinence, defined as sexual inactivity consistent with the preferred and usual lifestyle of the subject. Periodic abstinence (e.g. calendar, ovulation, symptothermal, post-ovulation methods) and withdrawal are not acceptable methods of contraception.

The following are considered highly effective (failure rate <1%) contraceptive methods:

- Oral contraceptive, either combined or progesterone alone
- Injectable progestogen
- Implants of etonogestrel or levonorgestrel
- Estrogenic vaginal ring
- Percutaneous contraceptive patches
- Intrauterine device (IUD) or intrauterine system (IUS) that meets the <1% failure rate as started in the product label.

10.3.10 **Medical history and physical examination**

At screening, a comprehensive history of previous illness and any medical or surgical interventions will be recorded. A medical history report will be obtained from the volunteer's GP. A directed physical examination will be performed at the visits specified in the schedule of procedures or as clinically indicated. This will include examination of skin, mucous membranes, lymph nodes, respiratory, cardiovascular, abdominal and/or central nervous systems as indicated by history or observation.

10.3.11 **Blood and urine collection**

Approximately 940ml of blood will be drawn over the study period of approximately six months including the screening, treatment and follow-up periods. The volume will vary from 16 to 85ml per visit. The specific blood tests are detailed in the schedule of procedures and in section 10.4 below. Urine will be analysed using a standard NHS urinalysis dipstick at screening.

10.3.12 **Laboratory results**

All safety laboratory (RFH laboratories) results will be reviewed and the reports signed by the CI/designee who will record on the source document whether they are normal, abnormal but not clinically significant, or abnormal AND clinically significant. For clinically significant abnormalities, actions taken must be recorded in the source document; the eligibility of the participants to continue in the trial must be reviewed, and the decision documented in the source document as well. Only abnormal laboratory results will be transcribed into the CRF.

10.4 Blood sample processing

Detailed information on sample processing and shipping will be included in a trial-specific manual.

10.4.1 Routine biochemistry and haematology

Table 2 below provides detail regarding what test will be performed. Blood will be drawn in the appropriate Vacutainer® tubes at every visit and processed according to local laboratory SOPs. All samples will be processed at the RFH clinical haematology and biochemistry laboratories. Blood samples will be analysed on the day of draw as per routine clinical practice.

Laboratory Parameter	Test
Haematology	Full blood count (haemoglobin, haematocrit, erythrocytes, leucocytes, platelets)<br><br>Differential count (absolute neutrophils, absolute lymphocytes)
Biochemistry	Liver function tests: (aspartate aminotransferase (AST), alanine aminotransferase (ALT), total and direct bilirubin). Kidney function tests: (creatinine) and urinalysis (see below)
Urinalysis (only at screening)	Dipstick test for protein, blood, glucose, ketones, leucocytes, nitrite. If abnormalities (blood, protein, leucocytes) are found in the dipstick test then further tests will be performed e.g. microscopy and culture

Table 2 Routine laboratory parameters

10.4.2 Virology

Blood will be drawn for testing for HBsAg, HCV, Syphilis and HIV-1 and -2 in the appropriate Vacutainer® tubes at the screening visit and processed according to local laboratory SOPs. Tests may be repeated if the CI/study physician suspects exposure or infection during the study. All tests will be carried out at the RFH clinical virology laboratories. Blood samples will be processed on the day of draw.

10.4.3 HLA typing

Blood will be drawn in the appropriate Vacutainer® tube at the screening visit and will be shipped at ambient temperature as soon as possible after draw to the immunology lab in Oxford. Samples will be analysed according to local laboratory SOPs.

10.4.4 Serum SAP levels

Blood will be drawn in the appropriate Vacutainer® tubes at particular visits indicated in Appendix A. Samples will be centrifuged on site, and the serum component aliquoted and stored at -70 to -90°C. Samples will be analysed at the CAAPP laboratory at the end of the study as per local SOPs.

10.4.5 Plasma CPHPC levels

Blood will be drawn in the appropriate Vacutainer® tubes at specific visits and time-points indicated in Appendix A. Samples will be centrifuged on site, and the plasma component transferred to matrix-

tubes and stored at -20°C. Samples will be batched and shipped periodically to GSK laboratories, on dry ice, for analysis according to local laboratory SOPs.

10.4.6 Cellular immunology assays

Blood will be drawn in the appropriate Vacutainer® tubes at every visit and will be shipped ambient as soon as possible after draw to the immunology lab in Oxford. Samples will be processed, and immunology assays will be performed, as per local laboratory SOP.
Samples remaining at the end of the study may be retained and used (anonymously) in future ethically-approved research, with written informed consent of the donors.

10.5 Assessments

10.5.1 Safety assessments

Data on local and systemic reactogenicity events related to the vaccinations will be collected using specific questions; these will be recorded as AEs. For CPHPC, local reactions observed at the cannula site will be recorded as AEs. Data on other events will be collected with an open question. All such data will be recorded as AEs in the source documents and case report forms.

10.5.2 Local reactogenicity events

The presence of local reactogenicity events will be assessed at the time points specified in the Appendix A. For the vaccinations, local reactogenicity events will be collected prospectively by questioning and examination at the vaccination visits and post-vaccination follow-up visits. For CPHPC, the infusion site will be monitored for signs of phlebitis, and the subject will be advised to report any local symptoms. Local reactogenicity events (pain, tenderness, erythema or skin discoloration, skin damage (vesiculation or ulceration), induration (formation of crust or scab) will be assessed and graded according to the Adverse Event Grading Toxicity Table (Appendix C). Local reactogenicity events will be recorded as AEs.

10.5.3 Systemic reactogenicity events

The presence of systemic reactogenicity events will be assessed at the time points specified in the Schedule of Procedures.
For vaccinations: Volunteers will record their temperature between 0-3 days post-vaccination in the study-specific diary (Appendix C). Systemic reactogenicity events will be collected prospectively by the scheduled telephone review, inspection of the diary and questioning and examination at the vaccination visits and post-vaccination follow-up visits. Vital signs will be measured by site personnel as specified in section 10.3 above as well as in Appendices A and B.
For CPHPC infusions: Site staff will monitor vital signs immediately before the start of the infusion, and then at half hourly intervals for the first two hours of the infusion, and hourly thereafter for the next four hours, and then at 12 and 24 hours.

Feverishness, chills, headache, nausea, vomiting, cardiovascular symptoms, malaise and myalgia will be graded according to the Adverse Event Grading Toxicity Table (Appendix C). A participant will be withdrawn from the trial if the adverse event meets the definition of a stopping criterion (see section 13.1). Systemic reactogenicity events will be recorded as AEs.

10.5.4 Other AEs

Occurrence of other AEs (including serious AEs, SAEs) will be collected following an open question to volunteers at the time points specified in the schedule of procedures. Volunteers will be asked to record any AEs experienced during the three days post-vaccination in the diary card (or in electronic form). Clinically significant abnormal laboratory results and clinically significant out of range vital signs will be recorded as AEs in the CRF. The adverse events will be graded as specified in the Adverse Event Grading Toxicity Table (Appendix C).

10.5.5 Routine laboratory parameters

Table 2 (above) shows the laboratory parameters that will be measured routinely. These will include haematology, biochemistry and urinalysis. The samples for these tests will be collected at the time points specified in the schedule of procedures.

10.5.6 Specific screening tests

Volunteers will be screened to exclude the following diseases:

- Hepatitis B: positive for hepatitis B surface antigen (HbsAg)
- Hepatitis C: positive for hepatitis C antibodies (HCV antibodies)
- Syphilis.

10.5.7 Immunogenicity Assessments

10.5.7.1 Cellular Responses

T cell responses will be determined initially by IFN-γ ELISPOT assay and, depending on the number of cells available, by further exploratory assays including investigation by intracellular cytokine staining and *in vitro* HIV-1 suppression assays (see section 8.2.3 for further detail).

10.5.8 Other assessments

10.5.8.1 HLA typing

HLA typing will be performed on samples collected at the time point specified in the schedule of procedures. This is a standard for T cell vaccine studies; as T cell epitopes will be mapped, it would be beneficial to know HLA class I (and II) restrictions. The local laboratory will perform the analyses in accordance with site-specific SOPs.

10.5.8.2 HIV antibody test

Samples will be tested by standard ELISA according to local SOPs at the time point specified in the schedule of procedures.

10.5.8.3 Diary cards

After each vaccination visit, prior to discharge from the NAC, subjects will be provided with thermometers and diary cards, and will be instructed on how to measure and record their body temperature for the next 3 days. They will also be asked to record any AEs experienced and concomitant medications taken during this period. Subjects may keep an electronic record (rather than paper) if they wish to, but must present (or e-mail) this for review at the scheduled visit.

10.6 Use of concomitant medications

Use of prescription or non-prescription drugs, including vitamins, herbal and dietary supplements (including St John's Wort) within seven days (or 14 days if the drug is a potential enzyme inducer) or five half-lives (whichever is longer) prior to the first dose of study medication, and during the treatment phase, is prohibited unless in the opinion of the CI the medication will not interfere with the study procedures or compromise subject safety. Medications specifically and completely prohibited are corticosteroids, immunosuppressants and anticoagulants for the duration of the study until the EOS/ET visit. Subjects will be advised that should they require a medicine (not specified below) they should ring a member of the study team to discuss this.

During the screening visit, subjects will be questioned about their 'usual medications', (e.g. paracetamol or aspirin occasional for headache, multivitamin tablets, etc.), and these will be recorded on the screening case report form. The subject will be allowed to continue their 'usual medications' over the course of the study as long as they do not meet the criteria for prohibited medications (above) or are not associated with an exclusion criterion (section 9.2).

Medications allowed for treatment of potential side effects from the vaccinations are e.g. oral paracetamol for coryzal symptoms and oral antihistamines or paracetamol for injection site reactions. All concomitant medications used during the trial will be recorded in the CRFs.

10.7 Rescue medications

None.

10.8 Randomisation and Blinding

Following informed consent and screening, once eligibility has been confirmed, subject study numbers will be assigned sequentially as each subject enters the study (this will be different from the screening number). Each study number will be used only once, i.e. replacement subjects will be

assigned a new number, not the same number as the subject they are replacing. Trial screening and participant logs will be maintained.

The first two subjects will be randomised one to active treatment arm, and one to placebo. All subjects thereafter will be randomly assigned to receive CPHPC or placebo by means of a simple blocked randomisation list generated by www.sealedenvelope.com. Allocation of a randomisation number (study subject number) will be performed by a designated member of the study team. The entire study team and the study subjects will be blinded with respect to allocation of CPHPC or placebo within the groups. CPHPC or placebo will be prepared by unblinded staff at RFH pharmacy preparative services. They will have access to the randomisation list and will be instructed not to discuss the identity of the study drug with any blinded member of the study team or with volunteers.

10.9 Definition of End of Study

The end of the study is the date of the last visit of the last participant.

10.10 Unblinding

In the event of a medical emergency or where the treating physician judges that knowledge of the group assignment would be necessary for the clinical management of the volunteer, the treating physician will be able to unblind the subject by accessing the code breaks for the trial that are held in an online system designed specifically for the study by www.sealedenvelope.com. The subject will carry (when not at the RFH) a 24-hour emergency contact card with details of the study and contact number for the Investigator. If the person required to unblind is not the CI or PI then that health care professional will notify the investigating team that unblinding is required for a trial subject and will be given access to the website or provided with the treatment details. Unblinding in the event of a SUSAR will be as per the Sponsor's SOP.

On receipt of the treatment allocation details the CI/designee/treating health care professional will treat the participant's medical emergency as appropriate. The CI/designee will document the breaking of the code and the reasons for doing so on the appropriate CRF, in the site file and in the source documents. It will also be documented at the end of the study in any final study report and/or statistical report.

The CI/designee will notify the JRO (acting on behalf of the Sponsor) in writing as soon as possible following the code break detailing the necessity of the code break. The written information will be disseminated to the IDMC for review.

All subjects will be given the 24-hour emergency contact card.

11 Investigational Medicinal Product

11.1 Name and description of investigational medicinal products

GSK2315698A (CPHPC), a SAP-depleter, is a palindromic bis (D proline) compound. Its chemical structure has been unambiguously identified: (R)-1-[6-[(R)-2-Carboxy-pyrrolidin-1-yl]-6-oxo-hexanoyl] pyrrolidine-2- carboxylic acid. GSK2315698 solution is a clear colourless solution which contains GSK2315698A at a concentration of 200 mg/mL. The solution is sterilised by filtration and aseptically filled into glass vials. The vials are sealed with rubber stoppers and aluminium seals. The bulk product is manufactured for this trial by GSK. The labelled final product vial is processed and QP released by RFH MIA IMP production unit.

Placebo to match CPHPC is prepared for the trial by the UCLH pharmacy preparation unit under aseptic conditions starting from UK-marketed 0.9% w/v saline solution.

The three vaccines are not blinded;
pSG2.HIVconsv DNA vaccine consists of the plasmid pSG2 and the HIVconsv gene. When the vaccine is injected into muscle it expresses the HIVconsv immunogen. The HIVconsv gene was constructed by assembling the 14 most conserved regions of the HIV-1 proteome into one chimeric protein. The HIVconsv gene is approximately 2.5 kbp in size, which is large enough to encode fourteen of the most highly conserved regions of the HIV-1 proteins, each 27 to 128 amino acids in length. Each segment is a consensus sequence from one of the four major HIV-1 clades A, B, C and D, which alternate to ensure equal clade coverage. Epitopes recognised by rhesus macaque and mouse CD8+ T cells, and a mAb were added to the C-terminus. The vaccine contain a gene encoding one or more antigens under the regulation of an eukaryotic enhancer/promoter and polyadenylation signals that confer appropriate expression of the immunogens. When injected into muscle, cells surrounding the injection site internalize the plasmid and transport the DNA to the nucleus where transcription occurs as it would in natural infection. The vaccine is produced by culture in an appropriate *E. coli* host strain. The product is supplied as sterile aliquots of DNA solution in phosphate buffered saline (PBS), pH 7.4 (4.4 mg/mL) is manufactured, labelled and QP released by Bristol Institute for Transfusion Sciences Clinical Biotechnology Centre, Bristol, UK.

ChAdV63.HIVconsv vaccine is a live recombinant replication-deficient chimpanzee adenovirus type 63 (AdCh63), containing the same immunogen as described above for pSG2.HIVconsv, assembled into one chimeric protein. The primary pharmacodynamic action of ChAdV63.HIVconsv is the induction of a cellular immune response which is potentially prophylactic for healthy subjects and therapeutic in individuals already infected with the HIV-1 virus. It is manufactured, labelled and QP released by the Clinical Biomanufacturing Facility (CBF), Churchill Hospital, Oxford which holds a MHRA MIA IMP license. The vaccine is produced by suspension culture in HEK293 cells.

MVA.HIVconsv is a recombinant modified vaccinia virus Ankara (MVA) containing the same immunogen assembled into one chimaeric protein, as described above for pSG2.HIVconsv and ChAdV63.HIVconsv. The MVA.HIVconsv vaccine is produced by IDT GmbH, Germany, by culture in

chick embryo fibroblasts (CEF) prepared from Specific Pathogen Free (SPF) embryonated hens' eggs. It will be labelled and QP released by the Oxford CBF.

11.2 Packaging, storage and shipment

- The drug product, **GSK2315698 (CPHPC)** solution, is a clear, colourless solution containing 200 mg/mL GSK2315698A in water and sodium hydroxide. The Solution will be provided in 5 mL, Type 1, clear glass vials closed with rubber stoppers and sealed with aluminium overseals. It is manufactured for the trial by GSK and supplied as bulk unlabeled stock with an accompanying intermediate QP release (for the concentrated solution) and delivered to RFH Manufacturing Unit which will perform labelling of the individual vials and will perform the final QP release.
- The vial has to be stored refrigerated 2°C - 8°C and protected from light.
- The GSK2315698 Solution will be diluted with 0.9% saline solution, to a concentration of 20 mg/mL, at the clinical study site before IV administration.
- Diluted solutions with concentrations in the range of 200 mg/mL to 0.4 mg/mL are given 48-hour in-use shelf life when stored at up to 25 C. The dilution will be performed at the RFH Pharmacy Department in the aseptic unit. The aseptic unit is located within the RFH, but if deemed necessary, the transfer will be using a refrigerated bag in order to guarantee that the cold chain is maintained.
- The placebo for CPHPC will be prepared by the above said preparation unit under the same conditions starting from UK-marketed 0.9% w/v saline solution.

Both the Active and the Placebo will be labelled by the preparative unit at RFL in a blinded fashion.

The MVA.HIVconsv vaccine is provided as a white cloudy solution formulated in Tris-saline buffer (10 mM Tris HCl, 140 mM NaCl, pH 7.7) at a concentration of 8.6 x 10^8 pfu/ml. The vaccine is supplied from the manufacturer as aliquots of 350 µl (0.35 ml) in sterile 2.0-ml clear glass injection vials sealed with 13-mm rubber stoppers and 13-mm aluminium caps. The extractable volume is 0.23 ml. Manufacture of the MVA.HIVconsv drug product was carried out under cGMP by IDT (Germany). The standard shelf life for this product is 24 months when stored at – 70 °C or below.

The ChAdV63.HIVconsv vaccine will be provided as a sterile suspension of virus (1.35 x 10^{11} vp/mL = 3.75x10^9 IU/mL)) in formulation buffer (10 mM histidine, 7.5% sucrose, 35mM NaCl, 1 mM MgCl2, 0.1% PS80, 0.1 mM EDTA, 0.5% EtOH, pH 6.6). The virus suspension is supplied by the manufacturer as sterile 0.65-mL aliquots in 2-mL clear glass vials, sealed with 13 mm bromobutyl rubber stoppers and 13 mm aluminium crimps. The standard shelf life assigned to ChAdV63-vectored vaccines manufactured by CBF under cGMP conditions is 24 months, when stored at -80°C.

The pSG2.HIVconsv DNA vaccine will be provided as a sterile solution of pDNA (4.4 mg/mL) in phosphate buffered saline pH 7.4 (Ph Eur 4005000). The proposed dose given in the trial will be 909 µL (4.0 mg)*. The DNA solution is supplied by the manufacturer as sterile 1.0-1.3 mL aliquots in 2 mL clear borosilicate glass injection vials, sealed with rubber stoppers and aluminium caps.

Manufacture of the drug product is carried out in accordance with the requirements of cGMP by the Clinical Biotechnology Centre.

**For practicality, the total volume of pSG2.HIVconsv DNA administered will be 900 μL, to give a total dose of 3.96 mg.*

The vaccines will be shipped to RFL pharmacy on dry ice with temperature logging. On arrival they will be transferred to monitored storage between -70°C and -90°C. CPHPC vials will be shipped under controlled conditions to the RFL pharmacy where they will be labelled by RFH MIA IMP unit and QP released. The vials will be stored on receipt at 2°C to 8°C Placebo will be used from stock.

11.3 Description and justification of route of administration and dosage

Routes and dosages of all IMPs have been tested and justified in previous clinical trials. The completed GSK phase I study in healthy volunteers has provided a well-tolerated regimen (40 mg infused over 24 hours) capable of reducing circulating SAP from the normal range of ~20-50 mg/L to less than 0.2 mg/L within 24 hours. In studies involving patients with amyloidosis, CPHPC was infused for 48 hours with no related adverse events. Thus our proposed infusion regimen of 40 mg/hour for 26 hours in HIV-CORE003 is anticipated to be both efficacious in depleting serum SAP and safe.

All subjects will receive three IM injections of 4 mg (909 μL)* of pSG2.HIVconsv DNA followed by one IM injection of 1.2×10^9 IU (0.32 ml) of ChAdV63.HIVconsv and one IM injection of 2.0×10^8 pfu (0.23ml) of MVA.HIVconsv. These are the same doses used in the HIV-CORE002 trial.
**For practicality, the total volume of pSG2.HIVconsv DNA administered will be 900 μL, to give a total dose of 3.96 mg.*

11.4 Dosage, dosage modifications and method of administration

All subjects will receive exactly the same dosages of IMPs and no dose modifications are planned. The vaccines will be administered by intramuscular injection at the time-points specified in the schedule of procedures. All subjects will receive three IM injections of 4 mg (909 μL)* of pSG2.HIVconsv DNA followed by one IM injection of 1.2×10^9 IU (0.32 ml) of ChAdV63.HIVconsv and one IM injection of 2.0×10^8 pfu (0.23ml) of MVA.HIVconsv. All vaccine doses will be injected into deltoid regions of both arms, half into each arm.
**For practicality, the total volume of pSG2.HIVconsv DNA administered will be 900 μL (0.9 ml), to give a total dose of 3.96 mg.*

CPHPC/placebo will be given at the time-points specified in the schedule of procedures, by IV infusion via an indwelling cannula; 40 mg/hour CPHPC (20 mg/mL solution) will be infused over 26 hours.

Complete instructions for the handling and administration of IMPs are supplied in the site- and study-specific SOPs.

11.5 Dispensing and handling

The vaccines and CPHPC will be stored and dispensed according to site- and study-specific SOPs.

All vaccines will be used as supplied by the manufacturer with no further preparation. The vaccines will be thawed until completely liquid by holding the vial in the hand. The vials will not be shaken and the required volume will be drawn into the appropriate syringe. The vaccines will be administered within one hour of thawing.

CPHPC or matching placebo will be prepared and dispensed by RFH Pharmacy Department aseptic unit , after reconstitution and dilution, in ready-to-infuse syringes or bags labelled in a blinded fashion. Pharmacy will prepare a worksheet for the preparation based on the Protocol and IB. Further instructions will be detailed in a Pharmacy manual.

The ChAdV63.HIVconsv and MVA.HIVconsv vaccines are classified as genetically modified organism under the Genetically Modified Organisms (Contained Use) Regulations 2000.

A risk assessment of the activities will be carried out and reviewed by the local Genetic Modification Safety Committee. Local approval for the GMO activities will be gained before the site is opened to recruitment, and appropriate requirements for handling/containment/destruction will be in place.

11.6 Drug accountability

During the study an accountability log, dispensing log and log of used vials will be kept for each IMP; these logs will be monitored. All used vaccine and CPHPC vials will be kept in sealed bags for potential reconciliation by the Sponsor; they will be discarded by the study staff (in the case of the vaccine vials) and pharmacy (in the case of the CPHPC vials) according to local procedures, upon authorization from the Sponsor.

11.7 Assessment of compliance

As all IMPs will be administered at the study site by study personnel, subject compliance will not be an issue.

11.8 Post-trial IMP arrangements

As this is a non-therapeutic, PoC trial in healthy volunteers, there is no necessity for post-trial IMP arrangements.

12 AEs, SAEs and SUSARs

Collection, recording and reporting of AEs (including serious and non-serious events and reactions) to the sponsor will be completed according to the sponsor's SOP.

12.1 Definitions

Term	Definition
Adverse Event (AE)	Any untoward medical occurrence in a patient or clinical trial subject administered a medicinal product and which does not necessarily have a causal relationship with this treatment.
Adverse Reaction (AR)	Any untoward and unintended response in a subject to an investigational medicinal product which **is related** to any dose administered to that subject. *This includes medication errors, uses outside of protocol (including misuse and abuse of product)*
Serious adverse event (SAE), serious adverse reaction (SAR) or unexpected serious adverse reaction	Any adverse event, adverse reaction or unexpected adverse reaction, respectively, that:<br>• results in death,<br>• is life-threatening,<br>• requires hospitalisation or prolongation of existing hospitalisation,<br>• results in persistent or significant disability or incapacity, or<br>• consists of a congenital anomaly or birth defect
Important Medical Event	These events may jeopardise the subject or may require an intervention to prevent one of the above characteristics/consequences. Such events should also be considered 'serious'.
Unexpected adverse reaction	An adverse reaction the nature and severity of which is not consistent with the information about the medicinal product in question set out:<br>(a) in the case of a product with a marketing authorization, in the summary of product characteristics for that product,<br>(b) in the case of any other investigational medicinal product, in the investigator's brochure relating to the trial in question.
SUSAR	Suspected Unexpected Serious Adverse Reaction

12.2 Severity grading of AEs

Severity grading of all AEs will be assessed and determined by the CI or designee. Criteria for grading the severity of AEs (as mild, moderate, severe and very severe) are listed in the Adverse Event Grading Toxicity Table (Appendix D). The principle used to develop the grading criteria is shown in Table 4 and the same principle should be used in grading any events that have not been anticipated in the Adverse Event Grading Toxicity Table.

Category	Definition
Mild	The adverse event does not interfere with the volunteer's daily routine, and does not require intervention; it causes slight discomfort
Moderate	The adverse event interferes with some aspects of the volunteer's routine, or requires intervention, but is not damaging to health; it causes moderate discomfort
Severe	The adverse event results in alteration, discomfort or disability which is clearly damaging to health

Table 3. Severity Grading Criteria for AEs.

12.3 Assessment of causality and expectedness

The assessment of relationship of adverse events to the administration of IMP is a clinical decision based on all available information at the time of the completion of the case report form. The assessment will be made by the CI/study physician. The following categories will be used to define the causality of the adverse event:

Category	Definition
Definitely:	There is clear evidence to suggest a causal relationship, and other possible contributing factors can be ruled out.
Probably:	There is evidence to suggest a causal relationship, and the influence of other factors is unlikely
Possibly	There is some evidence to suggest a causal relationship (e.g. the event occurred within a reasonable time after administration of the trial medication). However, the influence of other factors may have contributed to the event (e.g. the patient's clinical condition, other concomitant events).
Unlikely	There is little evidence to suggest there is a causal relationship (e.g. the event did not occur within a reasonable time after administration of the trial medication). There is another reasonable explanation for the event (e.g. the patient's clinical condition, other concomitant treatments).
Not related	There is no evidence of any causal relationship.
Not Assessable	Unable to assess on information available.

The IB for each IMP is the reference documents to be used to assess expectedness against the IMPs. The protocol will be used as the reference document to assess procedural expected events.

Category	Definition
Expected	An adverse event that is classed in nature as serious and which is consistent with the information about the IMP listed in the Investigator Brochure.
Unexpected	An adverse event that is classed in nature as serious and which is not consistent with the information about the IMP listed in the Investigator Brochure.

12.4 Procedural expected AEs

The following AEs may be expected as a result of study procedures.

- Intravenous cannulation: temporary discomfort or pain at the insertion site; phlebitis; bruising.
- Venepuncture: temporary discomfort or pain at the venepuncture site; bruising; papule formation.
- Intramuscular injection: temporary pain or discomfort at the injection site; temporary pain or discomfort radiating to lower arm on movement.
- Blood donation: Vasovagal response resulting in temporary feeling of weakness, dizziness, possible fainting episode.

12.5 Recording AEs

All AEs including SAEs occurring from the start of first CPHPC infusion up to the end of study follow-up will be recorded. All AEs occurring during the study, as observed by the study team or reported by the participant, whether or not attributed to study medication, will be recorded on the case report form and in the AE log. The following information will be recorded: description, date of onset and end date, severity, assessment of relatedness to study medication or other drug or device, and action taken. Follow-up and resolution information will be provided as necessary. For vital signs and laboratory tests, only those deemed to be clinically significant by the CI/study physician will be recorded as AEs. All AEs will be followed until resolution or the event is considered stable. All related AEs that result in a participant's withdrawal from the study or are present at the end of the study, will be followed up until a satisfactory resolution occurs. The CI/designee will follow the criteria specified in section 13 in making the severity assessment and the decision to withdraw a subject from the study. A participant may also voluntarily withdraw from treatment due to what he perceives as an intolerable AE. If either of these occurs, the participant will undergo, where possible, an end of study assessment and be given appropriate care under medical supervision until symptoms cease or the condition becomes stable. Clinically significant abnormalities in the results of laboratory tests will also be recorded as adverse events. If the results are not expected as part of IMP treatment, these will also be recorded as unexpected.

The severity of events and expectedness will be assessed by the CI/study physician using the criteria described above. Individual AEs will not be reported to the Sponsor and IDMC unless they are considered to be serious. The IDMC and Sponsor will be provided with the AE log for review at least once per quarter. Any partner pregnancy occurring during the clinical study and the outcome of the pregnancy will be reported to the Sponsor by means of a Pregnancy Form, as soon as possible and within one working day of the CI/designee becoming aware of it. Pregnancy reports will be forwarded

to GSK via the Sponsor. Pregnancies will be recorded in the source documents and CRF, and will be followed up for congenital abnormality or birth defect.

12.6 Reporting procedures

All SAEs and SUSARs will be notified to the sponsor within 24 hours of becoming aware of the event according to the sponsor's written SOP. All SAEs and SUSARs will be recorded in the source documents and the CRF, and the sponsor's AE log. The AE log will be reported to the sponsor at least once per year. All SAEs and SUSARs will be reported to the sponsor on the appropriate Sponsor's SAE form. The CI/designee will complete the SAE form and the form will be faxed to the sponsor on 020 3108 2312, or e-mailed to sae@ucl.ac.uk, within 24 hours of his / her becoming aware of the event. The SAEs and SUSARs, considered related to IMPs, will be forwarded to the relevant IMP suppliers. . The PI/designee will respond to any SAE queries raised by the sponsor as soon as possible. The Sponsor will report all SUSARs to the REC and MHRA according to the Sponsor's SOP. SUSARs that are fatal or life-threatening must be notified to the MHRA and REC within seven days after the Sponsor has learned of them. Other SUSARs must be reported to the REC and MHRA within 15 days after the sponsor has learned of them. All SAEs/SUSARs will also be reported by the CI/ designee to the IDMC within the same timelines.

12.6.1 Notification of deaths

All deaths will be reported to the Sponsor and IDMC irrespective of whether the death is related to the IMP. Reporting will occur within 24 hours of the CI/designee becoming aware of it.

12.6.2 Development Safety Update Reports

The sponsor will provide the main REC and the MHRA with Development Safety Update Reports (DSUR) which will be written in conjunction with the trial team, GSK and the Sponsor's office. The report will be submitted within 60 days of the Developmental International Birth Date (DIBD) of the trial each year until the trial is declared ended

12.6.3 Annual progress reports

An annual progress report (APR) will be submitted to the REC within 30 days of the anniversary date on which the favourable opinion was given, and annually until the trial is declared as having ended. The CI/designee will prepare the APR.

12.6.4 Notification of serious breaches to GCP and/or the protocol

A "serious breach" is a breach which is likely to affect to a significant degree:
(a) The safety or physical or mental integrity of the subjects of the trial, and/or
(b) The scientific value of the trial.
The Sponsor shall notify the licensing authority in writing of any serious breach of:
(a) The conditions and principles of GCP in connection with that trial, and/or

(b) The protocol relating to that trial, as amended from time to time, within seven days of becoming aware of that breach.
The Sponsor will be notified immediately of any case where the above definition applies during the trial conduct phase. The Sponsor's SOP on the 'Notification of violations, urgent safety measures and serious breaches' will be followed.

12.6.5 Reporting Urgent Safety Measures

If any urgent safety measures are taken the CI/Sponsor shall immediately and in any event no later than 3 days from the date the measures are taken, give written notice to the MHRA and the relevant REC of the measures taken and the circumstances giving rise to those measures.

13 Discontinuation/withdrawal of participants and stopping rules

13.1 Conditions under which a subject may be withdrawn from the trial

Each participant has the right to withdraw from the study at any time. Volunteers will be discontinued from further trial treatment for any of the following reasons:

1. Volunteer request
2. A disease, condition or an adverse event (including clinically significant abnormal laboratory value) that develops, regardless of relationship to the IMPs, if, in the opinion of the PI or designee, further CPHPC infusions or vaccinations would jeopardise the safety of the volunteer
3. Significant protocol deviation which the TMG believe may compromise the study data/results
4. Loss to follow up
5. Investigator discretion.

13.1.1 Follow-up after discontinuation

The reason for withdrawal will be recorded in the CRF. Where possible, study procedures for early termination will be performed (see schedule of procedures). If a volunteer develops a condition which may not necessarily be related to the IMP but which may cause the investigators to recommend discontinuation, the volunteer will continue to be followed up until any adverse event is judged to have resolved or stabilised. Any adverse event resulting in the discontinuation of a volunteer's participation will be followed up until, in the opinion of the CI/designee, it has resolved or stabilised, where possible. Volunteers who have received the vaccines and who co-incidentally acquire HIV-1 infection through sexual (or parenteral) exposure during the study will be followed as determined by the IDMC.

13.1.2 Replacement of subjects

Subjects who withdraw or are withdrawn after receiving a vaccination will not be replaced. Subjects who withdraw or are withdrawn prior to receiving a vaccination will be replaced; this includes subjects who have received the CPHPC infusion but have withdrawn prior to the vaccination. Screening and subject logs will be kept.

13.2 Stopping rules

The trial may be prematurely stopped or temporarily halted under the following conditions:

- If the CI, sponsor or the IDMC decide to suspend the trial pending safety review of an emergent issue(s)
- If the CI and Sponsor decide to stop the trial for safety, administrative or other reasons.

14 Management of HIV issues during and following the study

14.1 HIV testing

Only volunteers who are HIV-1 and -2seronegative at screening will participate in the study. The HIV screening tests and routine post-vaccination tests will be performed as specified in the schedule of procedures. If a volunteer during or after the study is found to be HIV-1 or -2 seropositive, a newly drawn blood specimen will be collected for confirmation. If a volunteer appears to have developed HIV-1 or -2 specific antibodies as a result of vaccination and these cause reactivity on a standard HIV ELISA test, he will be followed up until the ELISA test becomes negative. If a volunteer suspects he has been exposed to HIV during the study he would be advised to contact the trial nurse/physician/CI as soon as possible. A risk assessment would be carried out by the trial nurse/physician/CI and the volunteer would be offered an HIV antibody test at the trial site, or referred to a GUM clinic if he prefers this option. Written information concerning tests performed and results will be provided upon request. Volunteers who are found to be HIV seropositive at screening and volunteers who acquire coincidental HIV infection during the study will receive counselling and referral for support and/or care.

14.2 Social discrimination as a result of antibody response to IMP

The aim is to minimise the possibility of social discrimination in volunteers (if any) who develop vaccine-induced HIV antibodies and therefore test positive on a routine HIV test. The volunteer will be offered a volunteer card stating that he has participated in a vaccine study giving a site contact number in case of medical emergency.

15 Data handling

15.1 Data collection and record keeping at the trial site

Data will be collected by the site study personnel and recorded in the source document. Data will be transferred from the source document to the paper case report forms. Where information or data are first recorded on the case report form this may be the source document, data points for which this is applicable will be defined before the start of the trial. Other source documents include but are not limited to:

- Documentation of any existing conditions or past conditions relevant to eligibility
- Signed ICFs
- Reported laboratory results
- Diary cards

A file will be held at the trial site for each volunteer containing all the case report forms; source documents will be kept in a separate file. All essential documents will be kept in a secure location and retained as required by GCP and applicable local requirements.

It is the responsibility of the CI to ensure the accuracy of all data entered in the case report forms. The delegation log will identify all those personnel with responsibilities for data collection and handling, including those who have access to the trial database.

15.2 Data entry onto the trial database

Data will be entered from the case report form onto the trial database by the designated team members, as listed in the delegation log. The database will be held at the NAC. All data will be recorded in the case report form for transcription into the database; no data will be entered directly onto the database. To provide for real time assessment of safety, data will be entered as soon as reasonably feasible after a study visit but not before data monitoring and query resolution. Immunogenicity and PK results will not be available until the end of the study when the assays are performed, and so will not be entered onto the database until the end of the trial.

15.3 Data analysis

The data analysis plan will be developed by the trial statistician and agreed upon by the CI and study team prior to unblinding the study.

15.4 Confidentiality

All data will be handled in accordance with the UK Data Protection Act 1998. The case report forms will not bear the subject's name or other personal identifiable data. The subject's initials, date of birth and trial identification number will be used for identification.

15.5 Trial database

A Microsoft® Access® database will be used for data entry. Designated members of the research team will be responsible for data entry and quality. A single data entry with visual quality control checks method will be used to ensure validity and quality of data; the visual check will not be performed by the same person who has entered the data. . Data analysis will be performed by the trial statistician. The data will be stored on the secure UCL Medical School server which is backed up every day

For visits previously conducted at UCLH, site staff will enter data from source documents onto paper case report forms. Source data will remain on site; case report forms will be photocopied after data monitoring and query resolution; on completion of the treatment visits at the UCLH CRF, the original case report forms will be transferred to the NAC by designated members of the study team whilst the copies will remain at UCLH. Case report forms will not contain any participant identifying data. Where data are transferred electronically this will be in accordance with the UK Data Protection Act 1998 as well as UCL Information Security Policy and Trust Information Governance Policy. There will be a documented record of physical and electronic data transfer.

16 Quality control and quality assurance

Regular monitoring will be performed in compliance with GCP requirements and in accordance with the study specific monitoring plan. The trial monitor(s) will verify that the study is conducted, recorded and reported in compliance with the protocol, the GCP and other applicable regulatory requirements. An independent audit of the study may be performed, if required by the sponsor.

The CI (by signing the protocol) and the volunteers (by giving informed consent) permit study-related monitoring, audits, Sponsor's reviews and regulatory inspections, and direct access to source documents. Such information will be treated as strictly confidential and will under no circumstances be made publicly available.

17 Statistical considerations

Stephen Gerry is the trial statistician who will be responsible for all statistical aspects of the trial from design through to analysis and dissemination.

17.1 Statistical analysis plan

A detailed statistical analysis plan will be developed by the study's professional statistician and agreed upon by the study team prior to unblinding the study.

17.2 Summary of baseline data

The primary immunogenicity readouts will be based on the determination of the frequencies of

HIVconsv-specific T cells expressed as the numbers of spot-forming units (SFU)/10^6 of peripheral blood mononuclear cells (PBMC) using the IFN-γ ELISPOT assay and 6 pools of HIVconsv-derived 15-mer peptides overlapping by 11 amino acids. The group frequencies will be summarized as means (and standard deviations), or medians (with lower and upper quartiles) of the group. Prior to the vaccination, baseline HIVconsv-specific frequencies will be determined and these are expected to be close to 0, because volunteers are HIV-1-uninfected and therefore are not expected to have HIV-1-specific T cell responses. Volunteers will be randomized into two groups in respect to the CPHPC treatment and the frequencies of HIVconsv-specific PBMC for each of these groups will be described separately. The no-treatment post-vaccination baseline for the CPHPC treatment will be the frequencies elicited by the CPHPC-untreated vaccinated group after DDD, DDDC and DDDCM regimens; these should be the same as those detected in the HIV-CORE002 for DDD,DDDC and for DDDCM regimens.

In HIV-CORE002, induction of HIV-1-specific responses was determined using a standardized IFN-γ ELISPOT assays. All three vaccines ChAdV63.HIVconsv, MVA.HIVconsv, and pSG2.HIVconsv DNA were immunogenic. Thus, relevant to this trial, frequencies induced by C alone peaked at a median of 612 spot-forming units (SFU)/10^6 PBMC. DDD alone did not induce any detectable T cell responses, but following a ChAdV63.HIVconsv boost (DDDC), peak median responses reached 1331 SFU/10^6 PBMC and confirmed the priming of HIV-1-specific responses by DDD (14). This low, but definite priming with pSG2.HIVconsv DNA vaccine provides an ideal baseline platform for detection of increased T cell frequencies by the CPHPC treatment. The mean +/- SD for peak responses in DDDCM are 5664+/-2091 SFU/10^6 PBMC.

17.3 Primary outcome analysis

The primary analysis will involve the calculation of the difference between SAP depleted and control group participants in $\log_{10}$ transformed IFN-γ ELISPOT data at 12 weeks. This will be back transformed and presented as an n-fold difference between treatment groups with associated 95% confidence interval. An analysis of variance will be used to compare the two groups.

17.4 Secondary outcome analysis

Although the ELISPOT assay is a very useful and well validated first-line approach to enumeration of specific T cells induced by vaccination, the relationship of IFN-γ production to protection against HIV-1-infection is uncertain. In the absence of simple functional T cell correlates of protection, the *in vitro* HIV-1-inhibition (HIA) assay is the most relevant functional T cell assay *in vitro*, measuring the ability of vaccine-induced CD8+ T cell to control HIV-1 infection in the cultured CD4+ T cells. All secondary outcome analyses will be descriptive.

All secondary outcome data will be analysed descriptively.

17.5 Primary safety analysis

The primary safety analysis will be descriptive.

18 Sample size

18.1 Sample size calculation

The proposed sample sizes take into account the time required to recruit healthy volunteers at four to five per month. The present study will be the first time that pSG2.HIVconsv DNA vaccine is given to humans undergoing SAP depletion and the effect size cannot be robustly predicted. Therefore this study will be primarily a pilot phase I trial of safety and immunogenicity providing descriptive results. With 20 participants in each group, and based on the results of the HIV-CORE002 study, Tables 4 and 5 show the power available to detect the given fold differences in numbers of SFU/10^6 of PBMC between the 2 groups.

Fold increase on raw values	Standard deviation (on $\log_{10}$ scale)	Power available after DDD with 20 participants in each group
4	0.82	62%
5		75%
10		96%

Table 4. Power available for the enhancement of DNA vaccination alone. All calculations assume a 5% significance level.

Fold increase on raw values	Standard deviation (on $\log_{10}$ scale)	Power available after DDDC with 20 participants in each group
2	0.37	71%
2.3		86%
3		98%
4		100%
5		100%
10		100%

Table 5. Power available for the enhancement of ChAdV63.HIVconsv boost following enhanced DNA priming. All calculations assume a 5% significance level.

18.2 Revised sample size calculations for addition of MVA.HIVconsv booster (M)

Tables 6, 7 and 8 show the revised sample size calculations taking into account the addition of the M booster.

a) Power available at 12 weeks with 20 participants in each group. Based on HIV Core-002 data from DDDCM group **0-12 weeks (after DDD):**

Mean peak response = 73.1, SD=67.1

Mean $\log_{10}$(peak response) = 1.53 SD=0.82

(Fold change= x_1/x_2

Log(fold change)=$\log(x_1/x_2)=\log(x_1)-\log(x_2)$ = log(2) for 2 fold increase = 0.301)

Fold increase on raw values	Difference to be detected from 1.53 ± SD=0.82 (on $\log_{10}$ scale)	Standard deviation (on $\log_{10}$ scale)	Increase in number of SFU/10^6 PBMC from mean peak in HIV-CORE002 of 73 ± SD=67	Power available after DDD with 20 participants in each group
4	0.602	0.82	219	62%
5	0.699		292	75%
10	1		658	96%

Table 6. Power available at 12 weeks with 20 participants in each group. Based on HIV Core-002 data from DDDCM group 0-12 weeks (after DDD). All calculations have assumed 5% significance level.

b) Power available after booster with 20 participants in each group. Based on HIV Core-002 data from DDDCM group **12-20 weeks (after DDDC):**

Mean peak response = 1644, SD=1287

Mean $\log_{10}$(peak response) = 3.09 SD=0.37

(Fold change= x_1/x_2)

Log(fold change)=$\log(x_1/x_2)=\log(x_1)-\log(x_2)$ = log(2) for 2 fold increase = 0.301)

Fold increase on raw values	Difference to be detected from 3.09 ± SD=0.37 (on $\log_{10}$ scale)	Standard deviation (on $\log_{10}$ scale)	Increase in number of SFU/10^6 PBMC from mean peak in HIV-CORE002 of 1644 ± SD=1287	Power available after DDDC with 20 participants in each group
2	0.301	0.37	1644	71%
2.3	0.362		2137	86%
3	0.477		3288	98%
4	0.602		4932	100%
5	0.699		6576	100%
10	1		14796	100%

Table 7. Power available after booster with 20 participants in each group. Based on HIV Core-002 data from DDDCM group 12-20 weeks (after DDDC). All calculations have assumed 5% significance level.

c) Power available after booster with 20 participants in each group. Based on HIV Core-002 data from DDDCM group **21-22 weeks (after DDDCM):**

Mean peak response = 5664, SD=2091

8 volunteers peaked at 6240, 7960, 5340, 4980, 4260, 7060, 1670, 7805 [SFU/10^6 PBMC]

Mean $\log_{10}$(peak response) = 3.71 SD= 0.22

(Fold change= $x_1/x_{2)}$

Log(fold change)=$\log(x_1/x_2)=\log(x_1)-\log(x_2)$ = log(2) for 2 fold increase = 0.301)

Fold increase on raw values	Difference to be detected from x.xx ± SD=y.yy (on log_{10} scale)	Standard deviation (on log_{10} scale)	Increase in number of SFU/10^6 PBMC from mean peak in HIV-CORE002 of 5664 ± D=2091	Power available after DDDC with 20 participants in each group
1.58	0.200	0.22	3285	80%
1.70	0.231		3965	90%
2	0.301		5664	99%
2.3	0.362		7363	100%
3	0.477		11328	100%
4	0.602		16992	100%

Table 8. Power available after booster with 20 participants in each group. Based on HIV Core-002 data from DDDCM group 21-22 weeks (after DDDCM). All calculations have assumed 5% significance level.

18.3 Randomisation methods

Subjects will be randomised using a simple block randomisation.

18.4 Planned recruitment rate

Based on the combined experience of recruiting healthy volunteers of the study team we anticipate that recruitment will be complete within one year of FPFV. This is equivalent to 3 to 5 volunteers recruited per month for the first year.

18.5 Interim analysis

There will be no interim analysis in this small phase I/IIa PoC trial.

18.6 Other statistical considerations

Any deviation(s) from the original statistical plan will be described and justified in the final report.

19 Names of committees involved in the trial

19.1 Trial Management Group

The role of the Trial Management Group (TMG) is to monitor all aspects of the conduct and progress of the trial, ensure that the protocol is adhered to and take appropriate action to safeguard participants and the quality of the data. The members of the TMG will comprise key members of the trial team including the research nurse and CI.

19.2 Independent Data Monitoring Committee

The role of the Independent Data Monitoring Committee (IDMC) is to review the accruing trial data at specified time-points and to assess whether there are any safety issues that should be brought to participants' attention or any reasons for the trial not to continue. In this trial the IDMC will convene to review safety data at three confirmed time-points:1) after the first two subjects have completed the first dosing visit; 2) after 20 subjects have completed at least one dosing visit; and at the end of the trial. An IDMC charter will be put in place with all the members of the IDMC for this trial.

20 Direct access to source data/documents

The CI and study sites will permit trial-related monitoring, audits, REC review, and regulatory inspections, providing direct access to source data/documents. Trial participants are informed of this during the informed consent discussion. Participants will consent to provide access to their medical notes.

21 Ethics and regulatory requirements

Issues with regards to HIV infection and/or HIV-1 antibody positivity are covered in section 14. No other ethical issues are anticipated.

The sponsor will ensure that the trial protocol, PIS, ICF, GP letter and submitted supporting documents have been approved, as required, by the appropriate regulatory body (MHRA in UK) and/or a REC, prior to any patient recruitment. The protocol and all agreed substantial protocol amendments, will be documented and submitted for ethical and/or regulatory approval prior to implementation. Before the site can enrol patients into the trial, the CI/designee will obtain approval from their NHS Trust Research & Development (R&D) department and be granted written permission. It is the responsibility of the CI/ designee to ensure that all subsequent amendments gain the necessary approval. This does not affect the individual clinician's responsibility to take immediate action if thought necessary to protect the health and interest of individual patients (see section 12.6.5 for reporting of urgent safety measures).

Within 90 days after the end of the trial, the CI/Sponsor will ensure that the REC and the MHRA are notified that the trial has finished. If the trial is terminated prematurely, those reports will be made within 15 days after the end of the trial. The CI will supply the Sponsor with a summary report of the clinical trial, which will then be submitted to the MHRA and main REC within 1year after the end of the trial.

22 Monitoring requirement for the trial

A trial-specific monitoring plan will be agreed prior to commencement of the trial. The trial will be monitored within the agreed plan.

23 Finance

The trial is funded by a Medical Research Council Developmental Clinical Studies grant.

24 Insurance

UCL holds insurance against claims from participants for injury caused by their participation in the clinical trial. Participants may be able to claim compensation if they can prove that UCL has been negligent. However, as this clinical trial is being carried out in a hospital, the hospital continues to have a duty of care to the participant of the clinical trial. UCL does not accept liability for any breach in the hospital's duty of care, or any negligence on the part of hospital employees. This applies whether the hospital is an NHS Trust or otherwise. Participants may also be able to claim compensation for injury caused by participation in this clinical trial without the need to prove negligence on the part of UCL or another party. Participants who sustain injury and wish to make a claim for compensation should do so in writing in the first instance to the CI, who will pass the claim to the Sponsor's Insurers, via the Sponsor's office. Hospitals selected to participate in this clinical trial shall provide clinical negligence insurance cover for harm caused by their employees and a copy of the relevant insurance policy or summary shall be provided to UCL, upon request.

25 Archiving

Archiving will be authorised by the Sponsor following submission of the end of study report.
The CI is responsible for the secure archiving of essential trial documents and the trial database as per UCL policy. All essential documents will be archived for a minimum of 5 years after completion of trial. Destruction of essential documents will require authorisation from the Sponsor.

26 Publication policy

All proposed publications will be discussed with Sponsor prior to publishing other than those presented at scientific forums/meetings.

27 Statement of compliance

The trial will be conducted in compliance with the approved protocol, the UK Regulations, EU GCP and the applicable regulatory requirement(s)

28 References

1. Pepys MB, Butler PJG. Serum amyloid P component is the major calcium-dependent specific DNA binding protein of the serum. Biochem Biophys Res Commun. 1987;148:308-13.

2. Gillmore JD, Hutchinson WL, Herbert J, Bybee A, Mitchell DA, Hasserjian RP, et al. Autoimmunity and glomerulonephritis in mice with targeted deletion of the serum amyloid P component gene: SAP deficiency or strain combination? Immunology. 2004;112:255-64.

3. Pepys MB, Herbert J, Hutchinson WL, Tennent GA, Lachmann HJ, Gallimore JR, et al. Targeted pharmacological depletion of serum amyloid P component for treatment of human amyloidosis. Nature. 2002;417:254-9.

4. Wang Y, Guo Y, Wang X, Huang J, Shang J, Sun S. Human serum amyloid P functions as a negative regulator of the innate and adaptive immune responses to DNA vaccines. J Immunol. 2011;186(5):2860-70.

5. Wang Y, Guo Y, Wang X, Huang J, Shang J, Sun S. Serum amyloid P component facilitates DNA clearance and inhibits plasmid transfection: implications for human DNA vaccine. Gene therapy. 2012;19(1):70-7.

6. Walker BD, Burton DR. Toward an AIDS vaccine. Science. 2008;320(5877):760-4.

7. Letourneau S, Im EJ, Mashishi T, Brereton C, Bridgeman A, Yang H, et al. Design and pre-clinical evaluation of a universal HIV-1 vaccine. PloS one. 2007;2(10):e984.

8. Knudsen ML, Mbewe-Mvula A, Rosario M, Johansson DX, Kakoulidou M, Bridgeman A, et al. Superior induction of T cell responses to conserved HIV-1 regions by electroporated alphavirus replicon DNA compared to that with conventional plasmid DNA vaccine. J Virol. 2012;86(8):4082-90.

9. Rosario M, Borthwick N, Stewart-Jones GB, Mbewe-Mvula A, Bridgeman A, Colloca S, et al. Prime-boost regimens with adjuvanted synthetic long peptides elicit T cells and antibodies to conserved regions of HIV-1 in macaques. Aids. 2012;26(3):275-84.

10. Rosario M, Bridgeman A, Quakkelaar ED, Quigley MF, Hill BJ, Knudsen ML, et al. Long peptides induce polyfunctional T cells against conserved regions of HIV-1 with superior breadth to single-gene vaccines in macaques. Eur J Immunol. 2010;40(7):1973-84.

11. Im EJ, Hanke T. MVA as a vector for vaccines against HIV-1. Expert Rev Vaccines. 2004;3(4 Suppl):S89-97.

12. Gillmore JD, Tennent GA, Hutchinson WL, Gallimore JR, Lachmann HJ, Goodman HJ, et al. Sustained pharmacological depletion of serum amyloid P component in patients with systemic amyloidosis. Br J Haematol. 2010 Jan 8.

13. Faurez F, Dory D, Le Moigne V, Gravier R, Jestin A. Biosafety of DNA vaccines: New generation of DNA vectors and current knowledge on the fate of plasmids after injection. Vaccine. 2010;28(23):3888-95.

14. Borthwick B, Ahmed T, Ondondo B, Hayes P, Rose A, Ibrahimsa U, Hayton E-J, Black A, Bridgeman A, Rosario M, Hill AVS, Berrie E, Moyle S, Frahm N, Cox J, Colloca S, Nicosia A, Gilmour

J, McMichael AJ, Dorrell L and Hanke T. Vaccine-elicited human T cells recognizing conserved protein regions inhibit HIV-1. *Mol Ther* 22:464-475 (2014).

15.Ondondo B, Brennen C, Nicosia A, Crome SJ and Hanke T. Absence of systemic toxicological changes following intramuscular administration of novel pSG2.HIVconsv DNA, ChAdV63.HIVconsv and MVA.HIVconsv vaccines to BALB/c mice. *Vaccine* 31: 5594– 5601 (2013)).

16. Hayton E-J, Rose A, Ibrahimsa U, Del Sorbo M, Capone S, Crook A, Black A, Dorrell L and Hanke T. Safety and tolerability of conserved region vaccines vectored by plasmid DNA, simian adenovirus and modified vaccinia virus Ankara administered to human immunodeficiency virus type 1-uninfected adults in a randomized, single-blind phase I trial. *PLoS ONE* (Submitted)

29 Appendices

29.1 Appendix A: Schedule of procedures

Study week		0	0	2	4	4	6	8	8	10	12	13	14	16	17	18	20
Visit number	1 Screening	2a	2b	3	4a	4b	5	6a	6b	7	8	9	10	11	12	13	EOS/ET 14
Visit window (days)	≤ -28	0	0	±3	0	0	±3	0	0	±3	±3	±3	±3	±3		±3	±3
Informed consent	x																
HIV risk assessment	x																
Medical history	x																
Weight and ECG	x																
Vital signs (blood pressure, heart rate and temperature)	x	x	x	x	x	x	x	x	x	x	x	x	x	x	x	x	x
Physical exam	x	x		x*	x		x*	x		x*	x*	x*	x*	x*	x*	x*	x
Concomitant medications	x	x		x	x		x	x		x	x		x	x	x	x	x
Adverse event monitoring		x	x	x	x	x	x	x	x	x	x	x	x	x	x	x	x
HLA typing	x																
HBsAg, HCV antibodies, Syphilis	x																
Serum HIV ELISA and HIV test counseling	x																
Routine haematology (FBC + differential)	x	x	x	x	x	x	x	x	x	x	x	x	x	x	x	x	x
Routine biochemistry (renal and liver profiles)	x	x	x	x	x	x	x	x	x	x	x	x	x	x	x	x	x
Serum SAP levels	x	x	x	x	x	x	x	x	x	x							
Plasma CPHPC levels			x			x			x								
Cellular immunology assays	x	x		x	x		x	x		x	x	x	x	x	x	x	x
Urinalysis (± microscopy & culture)	x																
Confirm eligibility	x	x															
Randomisation	x**																
CPHPC/Placebo 26 hr infusion			x			x			x								
DNA vaccine administration			x			x			x								
ChAdv63 HIVconsv booster vaccine administration											x						
MVA HIVconsv booster vaccine administration														x			
Local and systemic reactogenicity assessment			x	x	x	x	x	x	x	x	x	x	x	x	x	x	x
Issue diary card			x			x			x		x			x			
Review diary card				x			x			x			x		x		
Telephone review			x***			x***			x***		x***			x***			
* if indicated<br>** subject to successful screening, and before visit 2<br>*** day 3 to 5 after vaccination																	

29.2 Appendix B: Details of inpatient stay

Procedure	Time-point													
	Pre-infusion	0 min	30 min (±5min)	60 min (±5min)	90 min (±5min)	120 min (±10min)	4 hr (±10min)	6 hr (±15min)	12 hr (±15min)	24hr (±10min)	24 hr to 24 hr 30 min	30 min post-vaccine	2 hr post-vaccine	4hr post-vaccine
Admission to unit	x													
Physical exam*														
Medical history*														
Vital signs	x		x	x	x	x	x	x	x	x**		x	x	x
Routine biochemistry and haematology														
Cellular immunology assay														
Serum SAP levels										x**				
Plasma CPHPC levels										x**				
CPHPC/Placebo infusion		x	x	x	x	x	x	x	x	x			x	
Vaccination											x***			
Monitor for local/systemic reactogenicity		x	x	x	x	x	x	x	x	x	x	x	x	x
Discharge														x****

*Repeat at any time point if required
**Prior to vaccination
***30 minute flexibility window for vaccination from time-point 24 hr
****If vital signs and reactogenicity assessments within normal range

29.3 Appendix C: Adverse Event Severity Assessment Criteria

Adapted from Division of AIDS, 2004

Abbreviations: ADL- activities of daily living; LLN – lower limit of normal; ULN – upper limit of normal

**CLINICAL**				
**PARAMETER**	**GRADE 1**	**GRADE 2**	**GRADE 3**	**GRADE 4**
**SYSTEMIC EVENTS**				
Acute systemic allergic reaction	Localised urticaria; no medical intervention indicated	Localised urticaria with medical intervention indicated OR mild angioedema with medical intervention indicated	Generalised urticaria OR angioedema with medical intervention indicated OR symptomatic mild bronchospasm	Acute anaphylaxis OR life-threatening bronchospasm OR laryngeal oedema
Chills / rigors	Symptoms causing minimal or no interference with ADL	Symptoms causing greater than minimal interference with ADL	Symptoms causing inability to perform ADL	NA
Fatigue / Malaise	Symptoms causing minimal or no interference with ADL	Symptoms causing greater than minimal interference with ADL	Symptoms causing inability to perform ADL	Incapacitating symptoms causing inability to perform basic self-care
Pain (other than pain at injection site) – indicate body site	Pain causing minimal or no interference with ADL	Pain causing greater than minimal interference with ADL	Pain causing inability to perform AD	Disabling pain causing inability to perform basic self-care OR requiring hospitalisation (other than to Accident and Emergency Dept)

Headache	Symptoms causing minimal or no interference with ADL	Symptoms causing greater than minimal interference with ADL	Symptoms causing inability to perform ADL	Symptoms causing inability to perform basic self-care OR requiring hospitalisation (other than to Accident and Emergency Dept) OR headache with significant impairment of alertness or other neurological function
Fever (non-axillary)	37.7 – 38.6°C	38.7 – 39.3°C	39.3 – 40.5°C	> 40.5°C
**INJECTION SITE REACTIONS**				
Injection site pain (pain without touching) OR Tenderness (pain when area is touched)	Pain / tenderness causing no or minimal limitation of use of limb	Pain / tenderness limiting use of limb OR causing greater than minimal interference with ADL	Pain / tenderness causing inability to perform ADL	Pain / tenderness causing inability to perform basic self-care OR requiring hospitalisation (other than to Accident and Emergency Dept) indicated
Pruritus associated with injection	Localised to injection site and relieved spontaneously or with < 48 hours' treatment	Itching beyond the injection site but not generalised OR localised requiring > 48 hours' treatment	Generalised and causing inability to perform ADL	NA
Localised injection site reaction	Erythema or induration 5x5 cm-9x9 cm	Erythema or induration or oedema > 9 cm, any diameter	Ulceration OR secondary infection OR phlebitis OR sterile abscess OR drainage	Necrosis (involving dermis or deeper tissues)
**SKIN**				

Cutaneous reaction - rash	Localised macular rash	Diffuse macular or maculo-papular rash OR Target lesions	Diffuse macular or maculo-papular rash with vesicles or limited number of bullae OR superficial ulcerations of mucous membrane limited to one site	Extensive or generalised bullous lesions OR Stevens-Johnson syndrome OR ulceration of mucous membrane involving two or more distinct mucosal sites or toxic epidermal necrolysis
Pruritis (itching, no skin lesions)	Itching causing no or minimal interference with ADL	Itching causing greater than minimal interference with ADL	Itching causing inability to perform ADL	NA
Alopecia	Thinning detectable by study participant	Thinning or patchy loss detectable by healthcare provider	Complete hair loss	NA
**CARDIOVASCULAR**				
Cardiac Arrhythmia	Asymptomatic and NO intervention indicated	Asymptomatic AND non-urgent medical intervention indicated	Symptomatic, non-life-threatening AND non-urgent medical intervention indicated	Life-threatening arrhythmia OR urgent intervention indicated
Ischaemia / infarction	NA	NA	Symptomatic ischaemia (stable angina) OR testing consistent with ischaemia	Unstable angina OR acute myocardial infarction
Haemorrhage (significant acute blood loss)	NA	Symptomatic AND no transfusion indicated	Symptomatic AND transfusion of ≤ 2 units packed RBCs	Life-threatening hypotension OR transfusion of > 2 units packed RBCs

Hypertension (confirmed on repeat testing at same visit)	> 140-159 mmHg systolic OR > 90-99 mmHg diastolic	> 160-179 mmHg systolic OR > 100-109 mmHg diastolic	> 180 mmHg systolic OR > 110 mmHg diastolic	Life-threatening consequences e.g. malignant hypertension OR hospitalisation indicated (other than visit to Accident & Emergency dept.)
Hypotension	NA	Symptomatic, corrected with oral fluid replacement	Symptomatic, IV fluids indicated	Shock requiring use of vasopressors or mechanical assistance to maintain blood pressure
Pericardial effusion	Asymptomatic, small effusion requiring no intervention	Asymptomatic, moderate or larger effusion requiring no intervention	Effusion with non-life threatening functional consequences OR effusion with non-urgent intervention indicated	Life-threatening consequences e.g. tamponade OR urgent intervention indicated
Thrombosis / embolism	NA	Deep vein thrombosis AND no intervention indicated	Deep vein thrombosis AND intervention indicated (e.g. anticoagulation, lysis filter, invasive procedure)	Embolic event (e.g. pulmonary embolism, life-threatening thrombus)
Ventricular dysfunction (congestive heart failure)	NA	Asymptomatic (diagnostic finding) AND intervention indicated	New onset symptoms OR worsening symptoms	Life-threatening congestive heart failure
**GASTROINTESTINAL**				

Anorexia	Loss of appetite without decreased oral intake	Loss of appetite associated with decreased oral intake but without significant weight loss	Loss of appetite associated with significant weight loss	Life-threatening consequences OR Aggressive intervention indicated (e.g., tube feeding or total parenteral nutrition)
Diarrhoea	Transient or intermittent episodes of unformed stools OR increase of ≤ 3 stools over baseline per 24 hour period	Persistent episodes of unformed to watery stools OR over baseline per 24 hour period increase 4-6 stools	Bloody diarrhoea OR increase of ≥ 7 stools per 24 hour period OR IV fluid replacement indicated	Life-threatening consequences e.g. hypotensive shock
Constipation	NA	Persistent constipation requiring regular use of dietary modifications, laxatives or enemas moderate	Requiring disimpaction	Life-threatening consequences e.g. Obstruction
Nausea	Transient (< 24 hours) or intermittent nausea with no or minimal interference with oral intake	Persistent nausea resulting in decreased oral intake for 24-48 hours	Persistent nausea resulting in decreased oral intake for > 48 hours OR aggressive rehydration indicated e.g. IV fluids	Life-threatening consequences e.g. hypotensive shock
Vomiting	Transient or intermittent vomiting with no or minimal interference with oral intake	Frequent episodes of vomiting with no or mild dehydration	Persistent vomiting resulting in orthostatic hypotension OR aggressive rehydration indicated e.g. IV fluids	Life-threatening consequences e.g. hypotensive shock

Oral discomfort / Dysphagia	Mild discomfort, able to eat usual diet	Symptoms causing altered dietary intake but no medical intervention indicated	Symptoms causing severely altered dietary intake with medical intervention indicated	Life-threatening reduction in oral intake
**NEUROLOGICAL**				
Alteration in personality / behaviour or mood (e.g. Agitation, anxiety, depression, mania, psychosis)	Alteration causing no or minimal interference with ADL	Alteration causing greater than minimal interference with ADL	Alterations causing inability to perform ADL	Behaviour potentially harmful to self or others (e.g. Suicidal or homicidal ideation or attempt, acute psychosis) OR causing inability to perform basic self-care
Altered mental status	Changes causing no or minimal interference with ADL	Mild lethargy or somnolence causing greater than minimal interference with ADL	Confusion, memory impairment, lethargy or somnolence causing inability to perform ADL	Delirium OR obtundation OR coma
Ataxia	Asymptomatic ataxia detectable on exam OR minimal ataxia causing no or minimal interference with ADL	Symptomatic ataxia causing greater than minimal interference with ADL	Symptomatic ataxia causing inability to perform ADL	Disabling ataxia causing inability to perform basic self-care
CNS ischaemia (acute)	NA	NA	Transient ischaemic attack	Stroke

Neuromuscular weakness (myopathy & neuropathy)	Asymptomatic with decreased strength on exam OR minimal muscle weakness causing no or minimal interference with ADL	Muscle weakness causing greater than minimal interference with ADL	Muscle weakness causing inability to perform ADL	Disabling muscle weakness causing inability to perform basic self-care OR Respiratory muscle weakness resulting in ventilator dependence
Neurosensory alteration (including paraesthesiae and painful neuropathy)	Asymptomatic with sensory alteration on exam or minimal paraesthesiae causing no or minimal interference with ADL	Sensory alteration or paraesthesiae causing greater than minimal interference with ADL	Sensory alteration or paraesthesiae causing inability to perform ADL	Disabling sensory alteration or paraesthesiae causing inability to perform basic self-care
**RESPIRATORY**				
Bronchospasm (acute)	Transient; FEV1 or peak flow reduced to 70 - 80%	FEV1 or peak flow 50 - 69%	FEV1 or peak flow 25- 49%	Cyanosis OR requiring intubation OR FEV1 or peak flow < 25%
Dyspnoea	Dyspnoea on exertion with no or minimal interference with ADL	Dyspnoea on exertion with greater than minimal interference with ADL	Dyspnoea at rest causing inability to perform ADL	Respiratory failure with ventilatory support indicated
**MUSCULOSKELETAL**				
Arthritis / arthralgia	Joint stiffness, swelling or pain causing no or minimal interference with ADL	Joint stiffness, swelling or pain causing greater than minimal interference with ADL	Joint stiffness, swelling or pain causing inability to perform ADL	Disabling joint stiffness, swelling or pain causing inability to perform basic self-care

Myalgia (non-injection site)	Muscle pain causing no or minimal interference with ADL	Muscle pain causing greater than minimal interference with ADL	Muscle pain causing inability to perform ADL	Disabling muscle pain causing inability to perform basic self-care
**OCULAR / VISUAL**				
Visual impairment (from baseline)	Visual impairment causing no or minimal interference with ADL	Visual impairment causing greater than minimal interference with ADL	Visual impairment causing inability to perform ADL	Disabling visual loss in affected eye(s)
Uveitis	Asymptomatic but detectable on exam	Symptomatic anterior uveitis OR medical intervention indicated	Posterior or pan-uveitis OR operative intervention indicated	Disabling visual loss in affected eye(s)
**ENDOCRINE / METABOLIC**				
Diabetes mellitus	NA	New onset without need to initiate medication OR modification of current medications to regain glucose control	New onset with initiation of medication indicated OR diabetes uncontrolled despite treatment modification	Life-threatening consequences e.g. Ketoacidosis, hyperosmolar non-ketotic coma
Hyperthyroidism OR Hypothyroidism	Asymptomatic	Symptomatic causing greater than minimal interfence with ADL OR thyroid suppression / thyroid replacement therapy indicated	Symptoms causing inability to perform ADL OR uncontrolled despite treatment	Life-threatening consequences (e.g. Thyroid storm / myxoedema coma)

**LABORATORY**				
**PARAMETER**	**GRADE 1**	**GRADE 2**	**GRADE 3**	**GRADE 4**
**HAEMATOLOGY**				
Haemoglobin	10.0-10.9 g/dL OR any decrease 2.5-3.4 g/dL	9.0-9.9 g/dL OR any decrease 3.5-4.4 g/dL	7.0-8.9 g/dL OR any decrease ≥ 4.5 g/dL	< 7.0 g/dL

Absolute neutrophil count	1.0-1.3 x 10^9/L	0.75-0.99 x 10^9/L	0.5-0.74 x 10^9/L	< 0.5 x 10^9/L
WBC, decreased<br>WBC, increased	2.0-2.5 x 10^9/L<br>13.0-14.99 x 10^9/L	1.5-1.99 x 10^9/L<br>15.0-19.99 x 10^9/L	1.0-1.49 x 10^9/L<br>20.0-30.0 x 10^9/L	< 1.0 x 10^9/L<br>> 30.0 x 10^9/L
Platelets, decreased	100.0-124.9 x 10^9/L	50.0-99.9 x 10^9/L	25.0-49.9 x 10^9/L	< 25.0 x 10^9/L
Fibrinogen, decreased	100-200mg/dL<br>OR 0.75-0.99 x LLN	75-99 mg/dL<br>OR 0.5-0.74 x LLN	50-74 mg/dL<br>OR 0.25-0.49 x LLN	< 50 mg/dL OR< 0.25 x LLN OR gross bleeding
Prothrombin time	1.1-1.25 x ULN	1.26-1.5 x ULN	1.51-3.0 x ULN	> 3.0 x ULN
Partial thromboplastin time	1.1-1.66 x ULN	1.67-2.33 x ULN	2.34-3.0 x ULN	> 3.0 x ULN
**CHEMISTRIES**				
Sodium:<br>High<br>Low	 146-150 mmol/L<br>130-135 mmol/L	 151- 154 mmol/L<br>125-129 mmol/L	 155-159 mmol/L<br>121-124 mmol/L	 > 160 mmol/L<br>< 120 mmol/L
Potassium:<br>High<br>Low	 5.6 – 6.0 mmol/L<br>3.0 – 3.4 mmol/L	 6.1 – 6.5 mmol/L<br>2.5 – 2.9 mmol/L	 6.6 – 7.0 mmol/L<br>2.0 – 2.4 mmol/L	 >7.0 mmol/L<br><2.0 mmol/L
Glucose, high (fasting and no prior diabetes)	6.1-6.9 mmol/l	7.0-13.9 mmol/l	14.0-27.9 mmol/l	> 28 mmol/l
Creatinine	1.1-1.3 x ULN	1.4-1.8 x ULN	1.9-3.4 x ULN	≥ 3.5 x UN
ALT	1.25-2.5 x ULN	2.6-5.0 x ULN	5.1-10.0 x ULN	> 10.0 x ULN
ALP	1.25-2.5 x ULN	2.6-5.0 x ULN	5.1-10.0 x ULN	> 10.0 x ULN
Bilirubin	1.25-2.5 x ULN	2.6-5.0 x ULN	5.1-10.0 x ULN	> 10.0 x ULN
GGT	1.25 – 2.5 x ULN	>2.5 – 5.0 x ULN	>5.0 – 10.0 x ULN	> 10.0 x ULN
Calcium (corrected for albumin)<br>High<br>Low	 2.65-2.88 mmol/l<br>1.95-2.10 mmol/l	 2.89-3.13 mmol/l<br>1.75-1.94 mmol/l	 3.14-3.38 mmol/l<br>1.53-1.74 mmol/l	 > 3.38 mmol/l<br>< 1.53 mmol/l
Phosphate	0.81 mmol/l - < LLN	0.65-0.8 mmol/l	0.32-0.64 mmol/l	< 0.32 mmol/l
Uric acid	0.45-0.59 mmol/l	0.6-0.71 mmol/l	0.72-0.89 mmol/l	> 0.89 mmol/l
Lactate	< 2.0 x ULN without acidosis	≥ 2.0 x ULN without acidosis	Increased lactate with pH < 7.3 without life-threatening consequences	Increased lactate with pH < 7.3 with life-threatening consequences

Pancreatic amylase	1.1-1.5 x ULN	1.6-2.0 x ULN	2.1-5.0 x ULN	> 5.0 x ULN
Cardiac troponin I (cTnI)	NA	NA	NA	Levels consistent with myocardial infarction or unstable angina as defined by the manufacturer
Cardiac troponin T (cTnT)	NA	NA	NA	≥ 0.2 ng/mL OR Levels consistent with myocardial infarction or unstable angina as defined by the manufacturer
Creatine kinase	3.0-5.9 x ULN	6.0-9.9 x ULN	10.0-19.9 x ULN	≥ 20.0 x ULN
**URINALYSIS**				
Proteinuria, random collection	1+	2-3+	4+	NA
Haematuria, microscopic	6-10 RBC/hpf	>10 RBC/hpf	Gross, with or without clots OR RBC casts	Transfusion required
