# Supplementary material for: Randomized phase I trial HIV-CORE 003: Depletion of serum amyloid P component and immunogenicity of DNA vaccination against HIV-1
Source: PLoS One. 2018 May 17;13(5):e0197299. doi: 10.1371/journal.pone.0197299 (PMC5957335; doi:10.1371/journal.pone.0197299)
Supplement: S3 Text — (PDF) [file pone.0197299.s007.pdf]

EU Clinical Trials Register

Clinical trial results:
A randomised double-blind, placebo-controlled phase I/IIa trial to investigate the effect of depletion of serum amyloid P component (SAP) on the immune response to DNA vaccination in healthy male volunteers.

Summary

EudraCT number	2012-004052-11
Trial protocol	
Global end of trial date	01 February 2016

Results information

Result version number	v1 (current)
This version publication date	
First version publication date	

Trial information

Trial identification

Sponsor protocol code	11/0455

Additional study identifiers

ISRCTN number	-
ClinicalTrials.gov id (NCT number)	NCT02425241
WHO universal trial number (UTN)	-

Notes:

Sponsors

Sponsor organisation name	University College London
Sponsor organisation address	Gower Street, London, United Kingdom,
Public contact	Dr Julian Gillmore, National Amyloidosis Centre, University College London, 0044 2074332726, j.gillmore@ucl.ac.uk
Scientific contact	Dr Julian Gillmore, National Amyloidosis Centre, University College London, 0044 2074332726, j.gillmore@ucl.ac.uk

Notes:

Paediatric regulatory details

Is trial part of an agreed paediatric investigation plan (PIP)	No
Does article 45 of REGULATION (EC) No 1901/2006 apply to this trial?	No
Does article 46 of REGULATION (EC) No 1901/2006 apply to this trial?	No

Notes:

Results analysis stage

Analysis stage	Final
Date of interim/final analysis	29 August 2017

Is this the analysis of the primary completion data?	Yes
Primary completion date	01 February 2016
Global end of trial reached?	Yes
Global end of trial date	01 February 2016
Was the trial ended prematurely?	No

Notes:

General information about the trial

Main objective of the trial:

This is a proof-of-concept study to determine whether depleting the normal plasma protein serum amyloid P component (SAP) will enhance the body's immune response to DNA vaccination.

Protection of trial subjects:

1. Participants had to undergo testing for HIV, hepatitis and syphilis as part of screening. They were counselled about this prior to testing, and would have been referred for appropriate followup and treatment if they tested positive.
2. Any vaccination has the potential to cause the following: Redness, pain, swelling, itching, bruising, a warm feeling; ‘flu like symptoms such as fever, chills, muscle aches and pains, headaches, nausea, dizziness and fatigue; Allergic reactions such as itchy rash, low blood pressure, sudden body swelling, serious breathing difficulty; A temporary ache around the injection site. Subjects were observed in clinic for two hours after vaccination and received a telephone call three days later to make sure they were well. Subjects were advised on how to manage these symptoms at home and were given 24 hour contact cards.
3. This was a study in healthy volunteers with no direct benefit from participation. To address this, participants will be compensated for their time and effort.
4. There could have been some discomfort from study procedures, such as venepuncture, intramuscular injection and intravenous cannulation. Every effort was made to ensure the volunteers were as comfortable as possible throughout their participation in the study.
5. Subjects were dosed within an inpatient facility with overnight monitoring. The first two subjects were randomised one to each arm and were dosed one at a time; the safety data from these two subjects were reviewed by the IDMC before any further subjects were dosed.
6. Participants underwent testing for HIV, hepatitis and syphilis as part of screening. They were counselled about this prior to testing, and were assured that they would be referred for appropriate follow up and treatment should a test be positive.

Background therapy:

None

Evidence for comparator:

Placebo comparator was used to determine whether SAP depletion had any effect on the response to DNA vaccination.

Actual start date of recruitment	22 April 2013
Long term follow-up planned	No
Independent data monitoring committee (IDMC) involvement?	Yes

Notes:

Population of trial subjects

Subjects enrolled per country

Country: Number of subjects enrolled	United Kingdom: 41
Worldwide total number of subjects	41
EEA total number of subjects	41

Notes:

Subjects enrolled per age group

In utero	0
Preterm newborn - gestational age < 37 wk	0
Newborns (0-27 days)	0
Infants and toddlers (28 days-23 months)	0
Children (2-11 years)	0
Adolescents (12-17 years)	0
Adults (18-64 years)	41
From 65 to 84 years	0
85 years and over	0

Subject disposition

Recruitment

Recruitment details:

Participants were recruited via advertisements - emails, posters and newspaper adverts.

Pre-assignment

Screening details:

Healthy, HIV negative men aged 18 to 50 were invited to contact the research team if they wished to participate in the study. After informed consent volunteers were screened for eligibility against the inclusion and exclusion criteria set out in the study protocol.

Period 1

Period 1 title	Baseline
Is this the baseline period?	Yes
Allocation method	Randomised - controlled
Blinding used	Double blind
Roles blinded	Subject, Investigator

Blinding implementation details:

An online randomisation service was used. Only the pharmacists were unblind to the allocation. The IMP and placebo were identical and so there was no danger of study staff or participants being unblinded. Laboratory tests of pharmacodynamic (PD) effect were only carried out at the end of the trial, after the official unblinding, i.e. PD samples taken throughout the study were frozen and stored for analysis at the end of the study.

Arms

Are arms mutually exclusive?	Yes
**Arm title**	CPHPC

Arm description: -

Arm type	Experimental
Investigational medicinal product name	(R)-1-[6-[(R)-2-carboxy-pyrrolidin-1-yl]-6-oxo-hexanoyl]pyrrolidine-2-carboxylic acid
Investigational medicinal product code	
Other name	CPHPC
Pharmaceutical forms	Concentrate for solution for infusion
Routes of administration	Intravenous drip use

Dosage and administration details:

40 mg/hour CPHPC (20 mg/mL solution) infused intravenously over 26 hours.

**Arm title**	Placebo

Arm description: -

Arm type	Placebo
Investigational medicinal product name	0.9% sodium chloride
Investigational medicinal product code	
Other name	
Pharmaceutical forms	Solution for infusion
Routes of administration	Intravenous use

Dosage and administration details:

2 mL/hr by intravenous infusion

Number of subjects in period 1	CPHPC	Placebo	
Started	20	20	
Completed	20	20	
Not completed	1	0	
Consent withdrawn by subject	1	-	
Joined	1	0	
Late recruitment	1	-	
Late recruitment reason	replacement of volunteer who withdrew consent		

Period 2

Period 2 title	Treatment
Is this the baseline period?	No
Allocation method	Randomised - controlled
Blinding used	Double blind
Roles blinded	Subject, Investigator

Blinding implementation details:

As per previous section.

Arms

Are arms mutually exclusive?	Yes
**Arm title**	CPHPC

Arm description:

Receiving active IMP, CPHPC, plus vaccines

Arm type	Experimental
Investigational medicinal product name	(R)-1-[6-[(R)-2-carboxy-pyrrolidin-1-yl]-6-oxo-hexanoyl]pyrrolidine-2-carboxylic acid
Investigational medicinal product code	
Other name	CPHPC
Pharmaceutical forms	Concentrate for solution for infusion
Routes of administration	Intravenous drip use

Dosage and administration details:

40 mg/hour CPHPC (20 mg/mL solution) infused intravenously over 26 hours.

Investigational medicinal product name	pSG2.HIVconsv
Investigational medicinal product code	
Other name	
Pharmaceutical forms	Solution for injection
Routes of administration	Intramuscular use

Dosage and administration details:

909 µL (4.0 mg) given by intramuscular injection

Investigational medicinal product name	ChAdV63.HIVconsv
Investigational medicinal product code	
Other name	
Pharmaceutical forms	Solution for injection
Routes of administration	Intramuscular use

Dosage and administration details:

1.2x10*9 IU (0.32 ml) given by intramuscular injection

Investigational medicinal product name	MVA.HIVconsv
Investigational medicinal product code	
Other name	
Pharmaceutical forms	Solution for injection
Routes of administration	Intramuscular use

Dosage and administration details:

2.0 x 10*8 pfu (0.23ml) given by intramuscular injection

**Arm title**	Placebo

Arm description:

Control arm receiving normal saline infusion plus vaccines

Arm type	Placebo
Investigational medicinal product name	0.9% sodium chloride
Investigational medicinal product code	
Other name	
Pharmaceutical forms	Solution for infusion
Routes of administration	Intravenous use

Dosage and administration details:

2 mL/hr by intravenous infusion

Investigational medicinal product name	pSG2.HIVconsv
Investigational medicinal product code	
Other name	
Pharmaceutical forms	Solution for injection
Routes of administration	Intramuscular use

Dosage and administration details:

909 µL (4.0 mg) given by intramuscular injection

Investigational medicinal product name	ChAdV63.HIVconsv
Investigational medicinal product code	
Other name	
Pharmaceutical forms	Solution for injection
Routes of administration	Intramuscular use

Dosage and administration details:

1.2x10*9 IU (0.32 ml) given by intramuscular injection

Investigational medicinal product name	MVA.HIVconsv
Investigational medicinal product code	
Other name	
Pharmaceutical forms	Solution for injection
Routes of administration	Intramuscular use

Dosage and administration details:

2.0 x 10*8 pfu (0.23ml) given by intramuscular injection

**Number of subjects in period 2**	CPHPC	Placebo	
Started	20	20	
Completed	19	20	
Not completed	1	0	
Consent withdrawn by subject	1	-	

Baseline characteristics

Reporting groups

Reporting group title	Baseline

Reporting group description:

Whole cohort

Reporting group values	Baseline	Total	
Number of subjects	41	41	
Age categorical			
Adult males aged 18 to 40			
Units: Subjects			
Adults (18-64 years)	41	41	
Gender categorical			
Males only			
Units: Subjects			
Male	41	41	
HIV status<br>Units: Subjects			
Negative	41	41	

End points

End points reporting groups

Reporting group title	CPHPC
Reporting group description: -	
Reporting group title	Placebo
Reporting group description: -	
Reporting group title	CPHPC
Reporting group description: Receiving active IMP, CPHPC, plus vaccines	
Reporting group title	Placebo
Reporting group description: Control arm receiving normal saline infusion plus vaccines	

Primary: Magnitude and breadth of differences in T cell frequencies between randomised groups

End point title	Magnitude and breadth of differences in T cell frequencies between randomised groups

End point description:

The magnitude and breadth of differences in T cell frequencies between randomised groups as measured by the ex vivo IFN-γ ELISPOT assay on peripheral blood mononuclear cells (PBMCs) of subjects

The primary analysis will involve the calculation of the difference between SAP depleted and control group participants in log10 transformed IFN-γ ELISPOT data at 12 weeks.

End point type	Primary

End point timeframe:

Peak ELISpot responses after ChAd63.HIVconsv and MVA.HIV.consv

**End point values**	CPHPC	Placebo		
Subject group type	Reporting group	Reporting group		
Number of subjects analysed	19	20		
Units: SFU/million				
median (full range (min-max))				
Magnitude of response after ChAd63.HIVconsv	1130 (40 to 6215)	835 (135 to 2785)		
Magnitude of response after MVA.HIVconsv	4020 (1440 to 9870)	3345 (830 to 20305)		
Breadth of response	6 (0 to 6)	4.5 (1 to 6)		

Statistical analyses

**Statistical analysis title**	Mann Whitney Test for non-parmetric data
Statistical analysis description: Comparison of medians from each group	
Comparison groups	CPHPC v Placebo

Number of subjects included in analysis	39
Analysis specification	Pre-specified
Analysis type	equivalence
P-value	< 0.05
Method	Wilcoxon (Mann-Whitney)
Parameter estimate	Median difference (final values)
Confidence interval	
level	95 %
sides	2-sided

Primary: Safety and tolerability- the number of Grade 3 or Grade 4 local or systemic reactions

End point title	Safety and tolerability- the number of Grade 3 or Grade 4 local or systemic reactions[1]

End point description:

The primary safety and tolerability endpoint will be the development of grade 3 or 4 (severe or very severe) local or systemic reactions after administration of CPHPC infusion followed by either of the HIVconsv vaccines. Due to the small number of participants, results will be purely descriptive.

End point type	Primary

End point timeframe:

0-20 weeks

Notes:

[1] - No statistical analyses have been specified for this primary end point. It is expected there is at least one statistical analysis for each primary end point.
Justification: Due to the number of participants in this study, it is intended that the primary safety analysis will be descriptive.

**End point values**	CPHPC	Placebo		
Subject group type	Reporting group	Reporting group		
Number of subjects analysed	19	20		
Units: AEs				
Grade 3	0	0		
Grade 4	0	0		

Statistical analyses

No statistical analyses for this end point

Secondary: Further characterization of the vaccine elicited immune responses

End point title	Further characterization of the vaccine elicited immune responses

End point description:

Although the ELISPOT assay is a very useful and well validated first-line approach to enumeration of specific T cells induced by vaccination, the relationship of IFN-γ production to protection against HIV-1-infection is uncertain. Luminex provides a sensitive measurement of interferon gamma and granzyme B, both associated with CD8+ anti-viral responses

End point type	Secondary

End point timeframe:

Measurements after DNA.HIVconsv and ChAd63.HIVconsv vaccination

**End point values**	CPHPC	Placebo		
Subject group type	Reporting group	Reporting group		
Number of subjects analysed	19	20		
Units: pg/ml				
median (full range (min-max))				
Granzyme response after DNA.HIVconsv	-15.1 (-45.15 to 22.25)	1.71 (-18.85 to 104.71)		
Granzyme response after ChAd63.HIVconsv	112.8 (35.5 to 464.5)	115.1 (55.8 to 303.7)		

Statistical analyses

**Statistical analysis title**	Wilcoxon Mann-Witney test
Statistical analysis description: Comparison of medians	
Comparison groups	CPHPC v Placebo
Number of subjects included in analysis	39
Analysis specification	Pre-specified
Analysis type	equivalence
P-value	< 0.05
Method	Wilcoxon (Mann-Whitney)
Parameter estimate	Median difference (final values)

Adverse events

**Adverse events information**	
Timeframe for reporting adverse events:<br>From first CPHPC infusion to end of study	
Adverse event reporting additional description:<br>Number of subjects who experienced adverse effects thought to be probably related or possibly related to CPHPC or vaccines.	
Assessment type	Systematic

Dictionary used

Dictionary name	Division of AIDS
Dictionary version	2004

Reporting groups

Reporting group title	CPHPC
Reporting group description:<br>Arm A, treatment group receiving CPHPC. Number of subjects who experienced non-serious adverse events deemed to be related or possibly related to CPHPC or vaccines.	
Reporting group title	Placebo
Reporting group description:<br>Related or possibly related non-serious adverse events in subjects who received placebo infusion and vaccines	

**Serious adverse events**	CPHPC	Placebo	
Total subjects affected by serious adverse events			
subjects affected / exposed	0 / 20 (0.00%)	0 / 20 (0.00%)	
number of deaths (all causes)	0	0	
number of deaths resulting from adverse events	0	0	

Frequency threshold for reporting non-serious adverse events: 5 %

**Non-serious adverse events**	CPHPC	Placebo	
Total subjects affected by non-serious adverse events			
subjects affected / exposed	13 / 20 (65.00%)	16 / 20 (80.00%)	
Cardiac disorders			
Hypertension			
subjects affected / exposed	1 / 20 (5.00%)	0 / 20 (0.00%)	
occurrences (all)	1	0	
Blood and lymphatic system disorders			
Enlarged lymph node in neck			
subjects affected / exposed	0 / 20 (0.00%)	1 / 20 (5.00%)	
occurrences (all)	0	1	

General disorders and administration site conditions			
Headache	Additional description: Headache		
alternative assessment type: Non-systematic			
subjects affected / exposed	4 / 20 (20.00%)	7 / 20 (35.00%)	
occurrences (all)	5	11	
Fatigue	Additional description: Fatigue, lethargy, malaise		
alternative assessment type: Non-systematic			
subjects affected / exposed	2 / 20 (10.00%)	5 / 20 (25.00%)	
occurrences (all)	2	6	
Insomnia			
alternative assessment type: Non-systematic			
subjects affected / exposed	1 / 20 (5.00%)	1 / 20 (5.00%)	
occurrences (all)	1	1	
Pain	Additional description: Stomach cramps		
alternative assessment type: Non-systematic			
subjects affected / exposed	1 / 20 (5.00%)	0 / 20 (0.00%)	
occurrences (all)	1	0	
Dizziness			
alternative assessment type: Non-systematic			
subjects affected / exposed	0 / 20 (0.00%)	1 / 20 (5.00%)	
occurrences (all)	0	1	
Fever			
subjects affected / exposed	0 / 20 (0.00%)	2 / 20 (10.00%)	
occurrences (all)	0	2	
Rigors	Additional description: Shivers		
alternative assessment type: Non-systematic			
subjects affected / exposed	0 / 20 (0.00%)	1 / 20 (5.00%)	
occurrences (all)	0	1	
Gastrointestinal disorders			
Diarrhoea			
alternative assessment type: Non-systematic			
subjects affected / exposed	1 / 20 (5.00%)	0 / 20 (0.00%)	
occurrences (all)	1	0	

Dysphagia	Additional description: Sore throat, enlarged tonsils		
subjects affected / exposed	2 / 20 (10.00%)	1 / 20 (5.00%)	
occurrences (all)	3	1	
Nausea			
alternative assessment type: Non-systematic			
subjects affected / exposed	1 / 20 (5.00%)	2 / 20 (10.00%)	
occurrences (all)	1	2	
Vomiting			
alternative assessment type: Non-systematic			
subjects affected / exposed	0 / 20 (0.00%)	1 / 20 (5.00%)	
occurrences (all)	0	1	
Anorexia	Additional description: Loss of appetite		
alternative assessment type: Non-systematic			
subjects affected / exposed	0 / 20 (0.00%)	1 / 20 (5.00%)	
occurrences (all)	0	1	
Renal and urinary disorders			
Abnormal odour in urine			
alternative assessment type: Non-systematic			
subjects affected / exposed	0 / 20 (0.00%)	1 / 20 (5.00%)	
occurrences (all)	0	1	
Skin and subcutaneous tissue disorders			
Cutaneous reaction - rash	Additional description: Rash, psoriasis		
subjects affected / exposed	2 / 20 (10.00%)	2 / 20 (10.00%)	
occurrences (all)	2	2	
Musculoskeletal and connective tissue disorders			
Injection site reaction	Additional description: Injection site pain (pain without touching)or tenderness (pain when area is touched)		
alternative assessment type: Non-systematic			
subjects affected / exposed	9 / 20 (45.00%)	12 / 20 (60.00%)	
occurrences (all)	13	17	
Myalgia	Additional description: Generalised aches and pains, muscle pains, 'flu-like symptoms		
alternative assessment type: Non-systematic			
subjects affected / exposed	7 / 20 (35.00%)	8 / 20 (40.00%)	
occurrences (all)	8	9	
Neck stiffness			

alternative assessment type: Non-systematic			
subjects affected / exposed	0 / 20 (0.00%)	1 / 20 (5.00%)	
occurrences (all)	0	1	
Infections and infestations			
Coryzal symptoms	Additional description: Cold, head cold		
alternative assessment type: Non-systematic			
subjects affected / exposed	0 / 20 (0.00%)	2 / 20 (10.00%)	
occurrences (all)	0	2	

More information

Substantial protocol amendments (globally)

Were there any global substantial amendments to the protocol? Yes

Date	Amendment
25 April 2013	Changes made in response to a grounds for non-acceptance letter from the MHRA (regarding the initial application).
04 July 2013	To document that any amendments submitted by GSK to the IMPD referred to in the cross-referral letter (dated 2 Jan 13) submitted in the original application will be applicable for this trial. GSK recently submitted an amendment to that IMPD allowing for an extension to the expiry date of the product.
02 December 2013	To extend the shelf life of ChAdV63.HIVconsv. The substantial amendment includes a revision of the data presented in the IMPD Section 2.1 Quality Data for ChAdV63.HIVconsv, Section 2.1.P.8 (Stability). The amendment also includes a proposal for further shelf life extensions in the future (when additional data become available), without the need to submit further substantial amendments.<br><br>The version and date of section 2.1 of the IMPD for the pSG2.HIVconsv DNA vaccine, and sections 2.2 and 2.3 of the IMPDs for the pSG2.HIVconsv DNA vaccine and the ChAdV63.HIVconsv vaccine have been updated to version 3.0, 28 Nov 2013, for consistency with section 2.1 of the IMPD for ChAdV63.HIVconsv vaccine, although no changes have been made to these sections of the IMPD.
03 September 2014	Transfer of Site Responsibilities from University College London Hospitals NHS Trust to Royal Free Hospital NHS Trust. Also addition of another IMP.
27 October 2014	Response to Notice of Acceptance with Condition for Amendment 7- MA Licensing)<br>Following the submission of Substantial Amendment 7 and Non-substantial amendment 6 to the MHRA, the MHRA responded with a Notice of Acceptance which was conditional. The condition was to provide additional documentation (Manufacturing authorisation) as listed above as another substantial amendment to the study.<br><br>Notification of IMPD changes<br>We have updated the IMPD to reflect that after the immunogenicity/potency tests that have been done on the MVA.HIV.consv vaccine this year (2014) to extend shelf life; we will continue doing these annually to determine whether to extend the shelf life. Shelf life will only be extended if the tests are within the product specifications.

Notes:

Interruptions (globally)

Were there any global interruptions to the trial? Yes

Date	Interruption	Restart date
21 July 2014	To transfer dosing responsibilities from University College London Hospitals NHS Trust to Royal Free Hospitals NHS Trust.	04 December 2014

Notes:

Limitations and caveats

None reported
