# Supplementary material for: Randomized phase I trial HIV-CORE 003: Depletion of serum amyloid P component and immunogenicity of DNA vaccination against HIV-1
Source: PLoS One. 2018 May 17;13(5):e0197299. doi: 10.1371/journal.pone.0197299 (PMC5957335; doi:10.1371/journal.pone.0197299)
Supplement: S4 Text — (PDF) [file pone.0197299.s008.pdf]

HIV-CORE 003: Statistical Report

Title: A randomised double-blind, placebo-controlled phase I/IIa trial to investigate the effect of depletion of serum amyloid P component (SAP) on the immune response to DNA vaccination in healthy male volunteers.

Sponsor: University College London

Chief Investigator at Jenner: Professor Tomas Hanke

Senior Immunologist at Jenner: Dr Nicola J. Borthwick

Version: 1.0

Date: 29 AUG 2017

CONFIDENTIAL

Introduction

HIV-CORE 003 is a proof of concept (PoC) clinical trial to test the impact of serum amyloid P component depletion by (R)-1-[6-[(R)-2-carboxypyrrolidin-1-yl]-6-oxo-hexanoyl]pyrrolidi ne-2-carboxylic acid (CPHPC) on the immune response to DNA vaccination in human volunteers. Although the ultimate goal is to make DNA vaccination a practicable and clinically effective approach in humans, the purpose of the present project is simply to establish PoC that SAP depletion augments the immune response induced by DNA vaccines. This report summarises the fresh ELISpot data from the HIV-CORE 003 study which took place in London, UK between October 2013 and February 2016.

1. Study Design

1.1. Vaccine schedule

Forty healthy, HIV-1-negative adult males of low risk of HIV-1 infection received experimental vaccines expressing HIV-1-derived immunogen HIVconsv delivered using plasmid DNA as pSG2.HIVconsv (D), engineered non-replicating simian adenovirus as ChAdV63.HIVconsv (C) and non-replicating poxvirus modified vaccinia virus Ankara as MVA.HIVconsv (M) combined into heterologous regimens (Table 1). All vaccines were administered intramuscularly using a needle injection. Prior to each DNA vaccine delivery, participants were randomized into one of two respective groups cD or D receiving a 24-hour infusion of either (R)-1-[6-[(R)-2-carboxypyrrolidin-1-yl]-6-oxo-hexanoyl]pyrrolidi ne-2-carboxylic acid (CPHPC) or saline as a placebo. CPHPC depletes the serum amyloid P component, which has the highest affinity for DNA of all the proteins present in human blood. The trial was initially designed to compare the cDcDcDC (Groups 1a; n = 4) and DDDC (Group 2a; n = 3) regimens, however, following recruitment of the first 7 volunteers, it was modified to the cDcDcDCM (Group 1b; n= 16) and DDDCM (Group 2b; n = 17) regimens with the aim to further amplify any obtained differences in immunogenicity by an additional MVA.HIVconsv boost (Table 1). Blood samples were taken at each visit for ELISpot analyses (Table 2)

Table 1: Vaccine schedule

Group	n	Week 0	Week 4	Week 8	Week 12	Week 16
1a	4	CPHPC pSG2.HIVconsv	CPHPC pSG2.HIVconsv	CPHPC pSG2.HIVconsv	ChAdV63.HIVconsv	
1b	16	CPHPC pSG2.HIVconsv	CPHPC pSG2.HIVconsv	CPHPC pSG2.HIVconsv	ChAdV63.HIVconsv	MVA.HIVconsv
2a	3	Placebo pSG2.HIVconsv	Placebo pSG2.HIVconsv	Placebo pSG2.HIVconsv	ChAdV63.HIVconsv	
2b	17	Placebo pSG2.HIVconsv	Placebo pSG2.HIVconsv	Placebo pSG2.HIVconsv	ChAdV63.HIVconsv	MVA.HIVconsv

Table 2: Bleeding schedule

**Group a**		**D**		**D**		**D**		**C**				
**Study week**	Screen	0	2	4	6	8	10	12	14	16	18	20
**Visit Number**	1	2	3	4	5	6	7	8	9	10	11	12
**ELISpot**	X	X	X	X	X	X	X	X	X	X	X	X

**Group b**		**D**		**D**		**D**		**C**			**M**			
**Study week**	Screen	0	2	4	6	8	10	12	13	14	16	17	18	20
**Visit Number**	1	2a	3	4a	5	6a	7	8	9	10	11	12	13	14
**ELISpot**	X	X	X	X	X	X	X	X	X	X	X	X	X	X

1.2. Ex vivo IFN-γ ELISpots

Blood processing and IFN-γ ELISpot analyses were performed at The Jenner Institute, University of Oxford, Oxford, UK. A panel of 199 overlapping 15 mer peptides covering the sequence of the HIVconsv immunogen were split into 6 pools and assayed in triplicate as shown (Fig. 1). Positive controls were wells containing PHA and a pool of commonly reactive Flu', EBV & CMV peptides (FEC). Negative controls (mock) were cells cultured without peptide. There was also an external positive control, NKL, a cell line that releases IFN-γ in response to stimulation with PMA & ionomycin.

Fig.1 Plate layout for the ELISpot assays

	1	2	3	4	5	6	7	8	9	10	11	12
A<br>B<br>C	PHA	P1	P3	P5	PHA	P1	P3	P5	PHA	P1	P3	P5
D<br>E<br>F	FEC	P2	P4	P6	FEC	P2	P4	P6	FEC	P2	P4	P6
G	Mock			NKL	Mock			NKL	Mock			NKL
H	Mock				Mock				Mock			

Sample 1
Sample 2
Sample 3

2. Definition of positive responses

2.1. Exclusion of samples from analysis

Prior to analysis, all samples were checked for extreme outliers (i.e. unusually high or low counts in the wells). Any control or peptide well whose individual replicate value is inconsistent with the median was excluded. For peptide wells and FEC control wells, any minimum or maximum outlier was excluded. For mock wells only maximum outliers were excluded. For example, where one replicate is discarded, the peptide pool response would be the median of the two remaining replicates and the mock response would be the median of the 5 remaining replicates.

2.2. Acceptance Criteria

INTERNAL POSITIVE CONTROL
PHA and FEC are included for each sample as internal positive controls. Wells containing PHA should show obvious staining such that they are "saturated"; indicated by TNTC by the ELISpot plate reader or have at least 100 SFU per well.

FEC (Flu, EBV, CMV) control individual participants responses over time and can be useful if it is suspected that samples have been confused or miss-labelled etc. FEC response do not however influence PASS/FAIL criteria unless there is no PHA response

EXTERNAL POSITIVE CONTROL
NKL cells are included as an external positive control to ensure that none of the steps have been missed during plate development. Plates should be failed if samples show no spots but NKL wells show the expected spots or if there are no spots at all.

INTERNAL NEGATIVE CONTROL

The six mock wells are the negative control. These should normally be median >100 SFU/million (20 spots/well @ 2.0 x10^5cells/well). Some people have a naturally high and consistent mock response and in this trial people are not excluded because of consistently high mock responses.

An ELISpot response is positive if the following criteria are fulfilled:

- There are at least two valid peptide replicates after discarding outliers
- The median number of background-subtracted spots in a set is greater than 38 SFU/10^6 PBMC
- Median SFU is greater than 4 x mock
- One pre-immune response is negative (i.e. there are no cross-reactive responses)

3. Sample size

Table 3 shows the number of samples included/excluded from the analysis at each visit. Of a total of 520 samples received, 11 (2.1%) failed

Table 3: Number of ELISpot assay results included in the analysis

Status	Group	**Total**	**Screen**	**0**	**2**	**4**	**6**	**8**	**10**	**12**	**13**	**14**	**16**	**17**	**18**	**20**
Pass	Group 1	251	17	20	20	19	20	18	17	19	16	19	19	16	15	16
	Group 2	258	20	19	20	19	18	20	20	20	16	20	18	16	16	16
	Total	509	37	39	40	38	38	38	37	39	32	39	37	32	31	32
Fail	Group 1	6	3			1		1	1							
	Group 2	5		1		1	2						1			
	Total	11	3	1	0	2	2	1	1	0	0	0	1	0	0	0
	**Total**	520	40	40	40	40	40	39	38	39	32	39	38	32	31	32

4. Results

4.1. Response Rates and Magnitude

Tables 4a and 4b show positive response rates to any peptide pool and the magnitude of the sum of the 6 peptide pools 1-6 in groups 1 and 2. This data is plotted in Fig. 2 below.

Table 4a: Proportion of positive ELISpot responses and descriptive statistics for Group 1, pools 1-6[a]

Week	N	% +ve	Mean	SD	Median	Min	Max
**Screen**	17	5.88	-11.47	64.63	0	-180	140
**0**	20	0	-16.75	52.8	-2.5	-140	45
**2**	20	0	-10.25	55.5	-10	-195	90
**4**	19	0	-12.63	44.83	0	-110	40
**6**	20	0	-14.5	41.45	-5	-115	60
**8**	18	0	-10.83	53.61	-7.5	-115	115
**10**	17	11.76	22.06	77.28	-5	-65	220
**12**	19	5.26	17.89	49.45	25	-75	130
**13**	16	62.5	1142	1811	317.5	-10	6215
**14**	19	94.73	1445	1271	890	40	4950
**16**	19	89.47	933.2	843.3	660	-5	2765
**17**	16	100	3563	1847	3105	710	7470
**18**	15	100	3865	2561	3670	500	9870
**20**	16	100	3455	2127	3318	190	7210

[a]*The units of analysis are positive response to any pool 1-6 per participant, and for magnitude, the sum of mean background-subtracted responses over pools 1-6 per participant. SD=Standard Deviation*

Table 4b: Proportion of positive ELISpot responses and descriptive statistics for Group 2, pools 1-6[a]

Week	N	% +ve	Mean	SD	Median	Min	Max
**Screen**	20	0	-3.125	58.54	10	-110	105
**0**	19	0	-13.16	48.34	-10	-115	65
**2**	20	0	-31.5	95.97	0	-340	130
**4**	19	0	-30	73.92	-20	-240	75
**6**	18	22.2	55.56	226.6	2.5	-220	810
**8**	20	15	44	105.3	20	-130	345
**10**	20	20	38.75	173.6	20	-230	630
**12**	20	10	21.38	71.67	15	-105	185
**13**	16	81.25	630.6	703.6	417.5	-295	2435
**14**	20	85	808.5	718.1	697.5	50	2385
**16**	18	100	653.6	610.4	465	85	2785
**17**	16	100	3363	3207	2700	830	14410
**18**	16	100	4714	4941	3295	730	20305
**20**	16	100	3457	3733	2630	290	14575

[a]*The units of analysis are positive response to any pool 1-6 per participant, and for magnitude, the sum of mean background-subtracted responses over pools 1-6 per participant. SD=Standard Deviation*

Fig. 2 Kinetics of IFN-γ ELISpot summed responses pools 1-6. The median, range and individual values are plotted for each visit

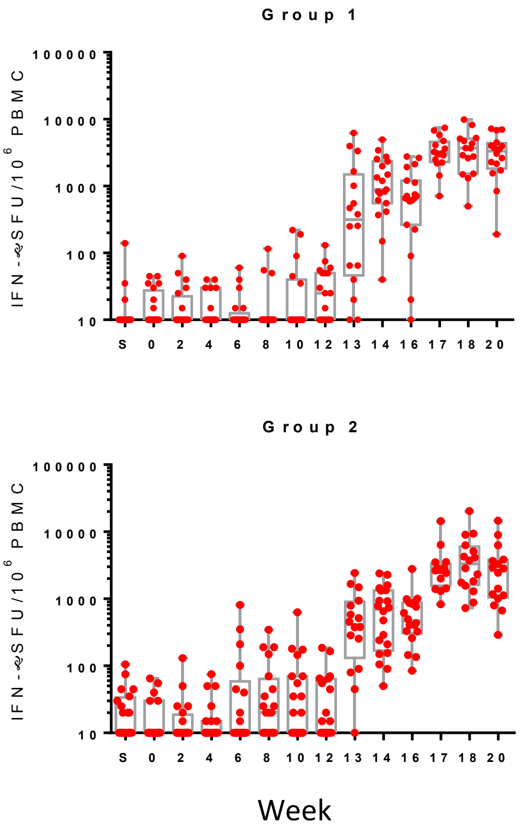

4.2 Comparison of peak responses

Peak responses, the greatest number of IFN-γ SFU/10^6 PBMC following vaccination, were identified for each participant as follows:
DNA.HIVconsv: visits 3-8 groups a & b
ChAd63.HIVconsv: visits 9-11 group a & b
MVA.HIVconsv: visits 12-14 group b
The peak values for each group at these three time points were compared using the Mann Whitney test for nonparametric data, using a significance level P=0.05

Table 4c IFN-γ ELISpot responses at peak response times, group 1, pools 1-6

Peak	N	Mean	SD	Median	Min	Max
**DNA**	19	62.11	59.26	45	5	220
**ChAd63**	19	1849	1703	1130	40	6215
**MVA**	16	4435	2379	4020	1440	9870

Table 4d IFN-γ ELISpot responses at peak response times, group 2, pools 1-6

Peak	N	Mean	SD	Median	Min	Max
**DNA**	20	111.5	190.2	47.5	-65	810
**ChAd63**	20	1003	812.5	835	135	2785
**MVA**	16	4982	4774	3345	830	20305

Table 4e Comparison of data at peak time points using the Mann Whitney test

	Mann Whitney U	P value	Significantly different?
**DNA.HIVconsv**	187	0.939	No
**ChAd63.HIVconsv**	137	0.1415	No
**MVA.HIV.consv**	114.5	0.6221	No

Fig. 3

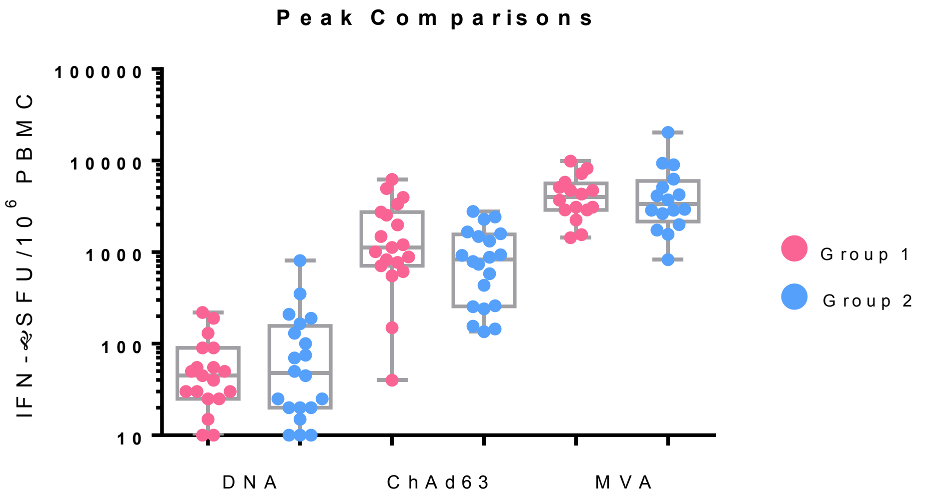

4.3 Responses to individual peptide pools

Peptide pool 1

Table 4f Proportion of positive ELISpot responses and descriptive statistics for Group 1, pool 1[a]

Week	N	% +ve	Mean	SD	Median	Min	Max
**0**	19	0	-4.47	13.76	-2.5	-37.5	10
**2**	19	0	-3.97	13.41	-2.5	-47.5	15
**4**	19	0	-1.58	6.729	0	-12.5	15
**6**	19	0	-1.84	8.243	-2.5	-20	10
**8**	18	0	-1.94	7.696	-1.25	-20	10
**10**	17	0	-2.21	12.65	-2.5	-32.5	20
**12**	19	0	2.90	10.08	2.5	-17.5	25
**13**	16	50	209.80	387.8	42.5	-2.5	1300
**14**	19	78.94	233.70	242	100	12.5	722.5
**16**	19	68.42	162.50	181.5	102.5	-2.5	732.5
**17**	16	100	541.30	509.6	380	60	2150
**18**	15	100	783.30	1022	435	40	4050
**20**	16	87.5	650.00	885	355	15	3430

[a]The units of analysis are positive response to pool 1 per participant, and for magnitude, the background-subtracted responses to pool 1 per participant. SD=Standard Deviation

Table 4g Proportion of positive ELISpot responses and descriptive statistics for Group 2, pool 1[a]

Week	N	% +ve	Mean	SD	Median	Min	Max
**0**	19	0	-3.711	11.19	-5	-28	20
**2**	20	0	-8.9	20.02	-3.75	-57.5	22.5
**4**	19	0	-8.289	17.08	-5	-57.5	12.5
**6**	18	11.1	9.972	52.56	-1.25	-50	197.5
**8**	20	20	14.75	32.1	3.75	-20	90
**10**	20	5	12.23	46.99	8.75	-40	190
**12**	20	10	6.125	19.08	3.75	-17.5	50
**13**	16	56.25	100.9	119	62.5	-32.5	380
**14**	20	70	92.5	77.6	82.5	-20	310
**16**	18	72.22	75.42	79.85	58.75	5	352.5
**17**	16	100	464.7	415.5	385	40	1600
**18**	16	100	570.9	728.2	332.5	80	3005
**20**	16	93.75	396.3	663	232.5	15	2770

[a]The units of analysis are positive response to pool 1 per participant, and for magnitude, the background-subtracted response to pool 1 per participant. SD=Standard Deviation

Fig. 4 Responses to Pool 1

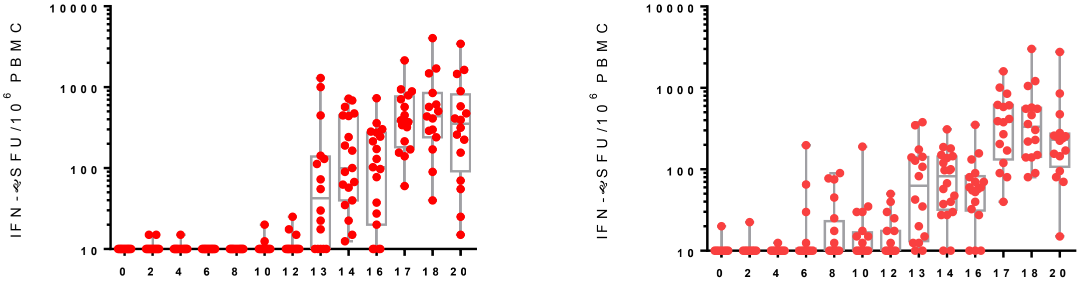

Group 1 Group 2

Peptide Pool 2

Table 4h Proportion of positive ELISpot responses and descriptive statistics for Group 1, pool 2[a]

Week	N	% +ve	Mean	SD	Median	Min	Max
**0**	19	0	-3.026	8.481	0	-22.5	10
**2**	19	0	0.1316	13.63	0	-37.5	30
**4**	19	0	-0.2632	8.287	2.5	-17.5	10
**6**	19	0	0.5263	8.763	0	-20	15
**8**	18	0	-3.611	9.441	-1.25	-25	15
**10**	17	5.88	1.912	11.74	0	-10	40
**12**	19	5.26	1.711	13.2	-2.5	-10	50
**13**	16	43.75	63.28	73.07	30	-2.5	207.5
**14**	19	68.42	165.9	288.9	77.5	2.5	1205
**16**	19	52.63	56.45	60.35	42.5	-12.5	242.5
**17**	16	100	310.6	386.4	217.5	45	1670
**18**	15	100	314	351.2	185	50	1370
**20**	16	81.25	220	223.2	180	5	785

[a]The units of analysis are positive response to pool 2 per participant, and for magnitude, the background-subtracted response to pool 2 per participant. SD=Standard Deviation

Table 4i Proportion of positive ELISpot responses and descriptive statistics for Group 2, pool 2[a]

Week	N	% +ve	Mean	SD	Median	Min	Max
**0**	19	0	-2.368	8.916	-2.5	-25	17.5
**2**	20	0	-7.375	22.59	-2.5	-70	17.5
**4**	19	0	-10.13	22.02	-2.5	-72.5	10
**6**	18	11.1	7.778	26.39	3.75	-35	87.5
**8**	20	5	8.5	16.11	5	-20	42.5
**10**	20	0	-2.5	19.16	-1.25	-47.5	30
**12**	20	0	2.875	9.743	1.25	-15	25
**13**	16	37.5	48.44	70.89	26.25	-37.5	242.5
**14**	20	40	69.25	110.5	18.75	-10	410
**16**	18	38.89	47.08	45.22	33.75	-7.5	155
**17**	16	81.25	255.3	265.3	162.5	10	870
**18**	16	75	290	349.1	197.5	-15	1335
**20**	16	68.75	206.9	353.5	110	0	1470

[a]The units of analysis are positive response to pool 2 per participant, and for magnitude, the background-subtracted response to pool 2 per participant. SD=Standard Deviation

Fig. 5 Responses to Pool 2

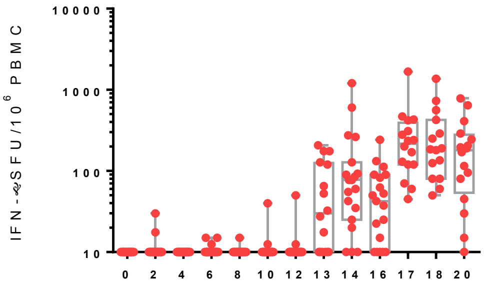

Group 1

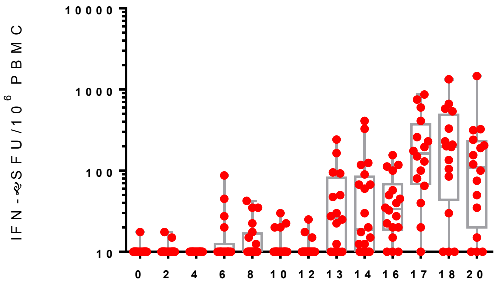

Group 2

Peptide Pool 3

Table 4j Proportion of positive ELISpot responses and descriptive statistics for Group 1, pool 3[a]

Week	N	% +ve	Mean	SD	Median	Min	Max
**0**	**19**	**0**	**-4.34**	**10.03**	**0**	**-22.5**	**12.5**
**2**	**19**	**0**	**-4.08**	**11.25**	**-2.5**	**-37.5**	**17.5**
**4**	**19**	**0**	**-5.79**	**12.42**	**-5**	**-35**	**17.5**
**6**	**19**	**0**	**-3.68**	**9.143**	**0**	**-22.5**	**10**
**8**	**18**	**0**	**-2.22**	**10.88**	**-5**	**-20**	**20**
**10**	**17**	**11.76**	**8.68**	**36.75**	**-2.5**	**-35**	**130**
**12**	**19**	**5.26**	**2.50**	**14.04**	**0**	**-15**	**42.5**
**13**	**16**	**56.25**	**218.90**	**470.4**	**51.25**	**-27.5**	**1910**
**14**	**19**	**89.47**	**334.10**	**508.1**	**187.5**	**12.5**	**2090**
**16**	**19**	**73.68**	**317.80**	**521.6**	**150**	**-7.5**	**2128**
**17**	**16**	**93.75**	**1249.00**	**1133**	**990**	**20**	**3455**
**18**	**15**	**100**	**1513.00**	**1535**	**812.5**	**50**	**4560**
**20**	**16**	**93.75**	**1535.00**	**1554**	**680**	**5**	**4415**

[a]The units of analysis are positive response to pool 3 per participant, and for magnitude, the background-subtracted response to pool 3 per participant. SD=Standard Deviation

Table 4k Proportion of positive ELISpot responses and descriptive statistics for Group 2, pool 3[a]

Week	N	% +ve	Mean	SD	Median	Min	Max
**0**	19	0	0.26	16.73	-5	-30	50
**2**	20	0	-6.63	19.16	-2.5	-65	17.5
**4**	19	0	-9.87	21.09	-7.5	-82.5	12.5
**6**	18	11.11	7.50	32.07	3.75	-67.5	75
**8**	20	10	3.75	23.45	0	-40	50
**10**	20	10	3.75	22.53	0	-47.5	45
**12**	20	0	2.38	11.54	3.75	-20	27.5
**13**	16	56.25	207.80	273.4	96.25	-42.5	797.5
**14**	20	70	285.80	395	122.5	0	1595
**16**	18	88.89	267.60	496.4	112.5	5	2180
**17**	16	100	1503.00	2461	515	250	10290
**18**	16	100	2317.00	3943	817.5	150	16220
**20**	16	100	1738.00	3080	497.5	95	12480

[a]The units of analysis are positive response to pool 3 per participant, and for magnitude, the background-subtracted response to pool 3 per participant. SD=Standard Deviation

Fig. 6 Responses to Pool 3

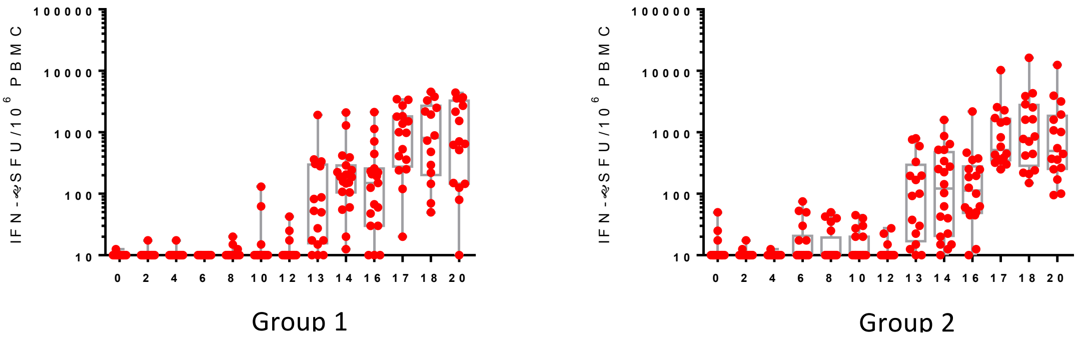

Peptide Pool 4

Table 4l Proportion of positive ELISpot responses and descriptive statistics for Group 1, pool 4[a]

Week	N	% +ve	Mean	SD	Median	Min	Max
0	19	0	-0.92	12.48	-2.5	-22.5	32.5
2	19	5.26	2.76	17.28	-2.5	-17.5	65
4	19	0	-0.53	11.07	2.5	-22.5	20
6	19	0	-4.21	10.34	-5	-20	15
8	18	0	0.83	13.53	0	-20	30
10	17	11.76	9.27	20.21	2.5	-10	52.5
12	19	0	5.92	10.74	5	-15	30
13	16	62.5	321.40	611.9	65	-12.5	2340
14	19	89.47	323.60	311.4	260	7.5	1060
16	19	84.21	205.90	326.5	110	2.5	1465
17	16	100	645.00	405.6	460	110	1490
18	15	100	534.60	482.6	355	80	1685
20	16	100	465.60	433.2	272.5	55	1325

[a]The units of analysis are positive response to pool 4 per participant, and for magnitude, the background-subtracted response to pool 4 per participant. SD=Standard Deviation

Table 4m Proportion of positive ELISpot responses and descriptive statistics for Group 2, pool 4[a]

Week	N	% +ve	Mean	SD	Median	Min	Max
0	19	0.00	-2.11	7.92	-5	-17.5	20
2	20	0.00	2.13	13.46	5	-20	37.5
4	19	5.26	1.71	17.87	-2.5	-32.5	57.5
6	18	11.10	20.00	60.62	6.25	-27.5	242.5
8	20	5.00	7.00	21.05	3.75	-35	67.5
10	20	10.00	10.00	36.80	3.75	-37.5	135
12	20	5.00	5.00	16.32	5	-17.5	55
13	16	62.50	139.40	175.60	80	-77.5	530
14	20	75.00	107.00	87.61	90	-7.5	282.5
16	18	66.67	74.86	53.63	66.25	-2.5	175
17	16	100.00	437.80	325.00	372.5	85	1150
18	16	100.00	600.90	800.10	195	65	3150
20	16	81.25	404.40	560.10	247.5	0	2245

[a]The units of analysis are positive response to pool 4 per participant, and for magnitude, the background-subtracted response to pool 4 per participant. SD=Standard Deviation

Fig. 7 Responses to Pool 4

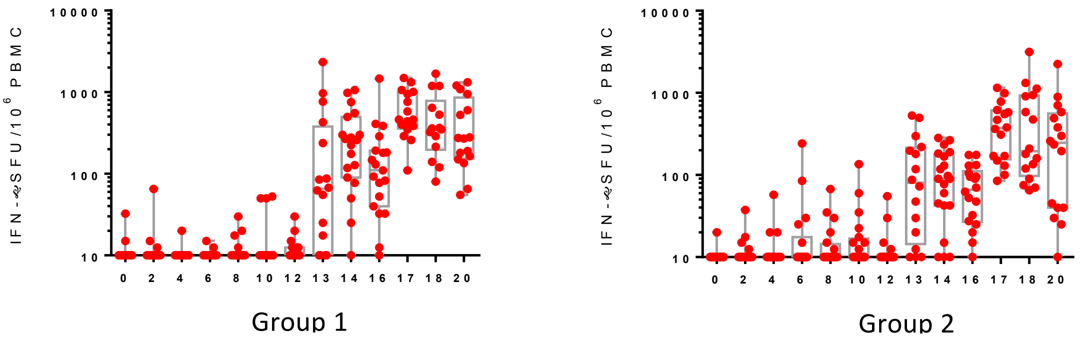

Peptide pool 5

Table 4n Proportion of positive ELISpot responses and descriptive statistics for Group 1, pool 5[a]

Week	N	% +ve	Mean	SD	Median	Min	Max
0	19	0	-1.45	12.43	0	-30	25
2	19	0	-4.87	8.719	-5	-27.5	12.5
4	19	0	-4.47	9.667	-2.5	-30	10
6	19	0	-4.74	9.387	-5	-25	12.5
8	18	0	-1.94	8.25	-1.25	-15	15
10	17	0	-0.44	9.406	0	-17.5	15
12	19	0	-1.71	9.393	0	-20	20
13	16	43.75	237.30	504.4	52.5	0	2010
14	19	84.2	216.70	269.2	115	-2.5	1060
16	19	68.42	100.70	166.3	65	-2.5	760
17	16	93.75	350.60	225.1	345	20	730
18	15	92.86	273.90	182.3	255	-5	635
20	16	87.5	236.90	210.5	187.5	-25	855

[a]The units of analysis are positive response to pool 5 per participant, and for magnitude, the background-subtracted response to pool 5 per participant. SD=Standard Deviation

Table 4o Proportion of positive ELISpot responses and descriptive statistics for Group 2, pool 5[a]

Week	N	% +ve	Mean	SD	Median	Min	Max
0	19	5.26	-3.684	18.75	-5	-37.5	45
2	20	0	-6.125	17.89	0	-55	17.5
4	19	0	-5.132	18.68	-2.5	-62.5	17.5
6	18	5.88	-1.389	26.03	-2.5	-82.5	45
8	20	0	3.5	18.5	6.25	-40	32.5
10	20	0	0.5	19.29	1.25	-42.5	50
12	20	0	0.375	9.976	-1.25	-25	20
13	16	43.75	57.19	101.3	30	-57.5	402.5
14	20	50	109.3	197.3	38.75	-10	670
16	18	27.78	72.92	117.8	30	2.5	417.5
17	16	100	327.8	342.4	157.5	40	1260
18	16	93.75	413.4	616.3	137.5	25	2175
20	16	87.5	313.8	551.2	140	10	2230

[a]The units of analysis are positive response to pool 5 per participant, and for magnitude, the background-subtracted response to pool 5 per participant. SD=Standard Deviation

Fig. 8 Responses to Pool 5

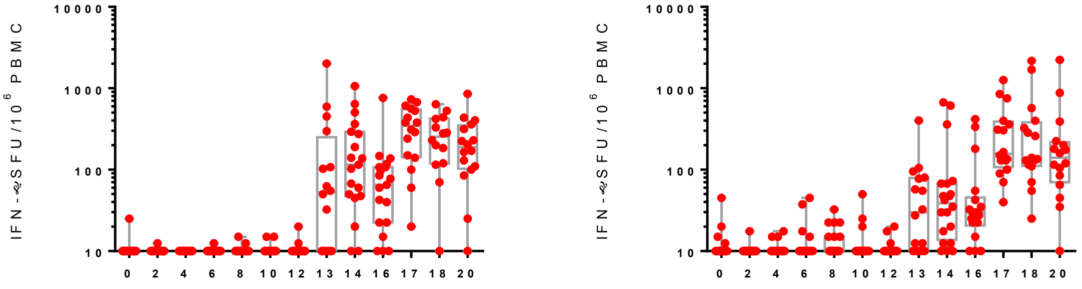

Group 1 Group 2

Peptide pool 6

Table 4p Proportion of positive ELISpot responses and descriptive statistics for Group 1, pool 6[a]

Week	N	% +ve	Mean	SD	Median	Min	Max
0	19	0	-1.97	11.77	0	-32.5	15
2	19	0	-1.97	9.26	-2.5	-27.5	10
4	19	0	-0.53	10.82	0	-22.5	20
6	19	0	-0.26	7.50	0	-15	12.5
8	18	0	-1.39	11.67	-1.25	-25	30
10	17	5.56	3.47	20.65	-2.5	-22.5	72.5
12	19	5.26	5.40	13.05	2.5	-12.5	40
13	16	37.5	91.09	161.10	23.75	-17.5	615
14	19	89.47	171.20	158.50	155	7.5	670
16	19	73.68	89.87	113.80	55	-7.5	507.5
17	16	100	466.90	370.70	407.5	60	1450
18	15	100	280.70	245.50	185	70	905
20	16	93.75	348.10	453.90	162.5	30	1760

[a]The units of analysis are positive response to pool 6 per participant, and for magnitude, the background-subtracted response to pool 6 per participant. SD=Standard Deviation

Table 4q Proportion of positive ELISpot responses and descriptive statistics for Group 2, pool 6[a]

Week	N	% +ve	Mean	SD	Median	Min	Max
0	19	0	-1.579	10.58	0	-20	25
2	20	0	-4.625	23.37	1.25	-85	22.5
4	19	0	1.711	12.53	0	-22.5	32.5
6	18	11.1	11.67	55.68	0	-77.5	192.5
8	20	5	6.5	25.58	5	-50	82.5
10	20	37.5	14.75	49.76	3.75	-32.5	210
12	20	5	4.625	18.91	0	-20	70
13	16	56.25	76.88	95.85	56.25	-47.5	322.5
14	20	70	144.8	157.1	80	-12.5	582.5
16	18	72.22	115.7	113.7	61.25	7.5	437.5
17	16	100	374.1	313.3	235	45	1160
18	16	93.75	522.2	554.8	282.5	25	1920
20	16	87.5	398.1	538.5	217.5	20	1905

[a]The units of analysis are positive response to pool 6 per participant, and for magnitude, the background-subtracted response to pool 6 per participant. SD=Standard Deviation

Fig. 9 Responses to Pool 6

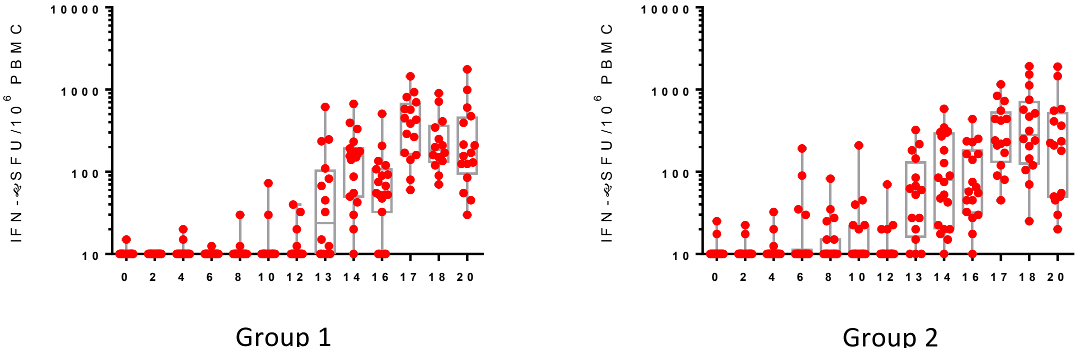

4.4 Breadth of response (number of pools recognised)

The number of pools recognised(SFU/million >38) at the peak following ChAd63.HIVconsv vaccination, visits 9-11, was used to compare the breadth of the response in each group. These were compared using the Mann Whitney test for nonparametric data, using a significance level P=0.05

Table 4r Comparison of the breadth of the response following ChAd63.HIVconsv vaccination

ChAd63.HIVconsv	N	Median	Min	Max	Mann Whitney U	P value	Significantly different?
Group 1	19	6	0	6	123.5	0.0444	Yes
Group 2	20	4.5	1	6			
